# The first intermolecular interrupted imino-Nazarov reaction: expeditious access to carbocyclic nucleoside analogues†
†Electronic supplementary information (ESI) available. See DOI: 10.1039/c5sc03559g


**DOI:** 10.1039/c5sc03559g

**Published:** 2015-10-27

**Authors:** Ronny William, Wei Lin Leng, Siming Wang, Xue-Wei Liu

**Affiliations:** a Division of Chemistry and Biological Chemistry , School of Physical and Mathematical Sciences , Nanyang Technological University , 21 Nanyang Link , Singapore 637371 . Email: xuewei@ntu.edu.sg

## Abstract

The analogous imino-variant of the Nazarov reaction suffers from unfavorable energetics associated with the ring closure process, thereby limiting the utility of this class of reactions. In this report, the realization of the first intermolecular interrupted imino-Nazarov reaction utilizing silylated pyrimidine derivatives as nucleophilic trapping partners culminated in an expedient synthetic route to carbocyclic nucleoside analogues. The potential application of silylated nucleobases to intercept the oxyallyl cation in other variants of the Nazarov reaction provides vast opportunities to develop new strategies for the formation of carbocyclic nucleoside analogues.

## Abstract 2

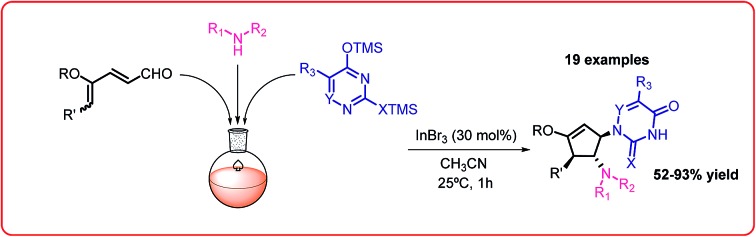
The first intermolecular interrupted imino-Nazarov reaction with silylated pyrimidine derivatives as nucleophile has been developed, furnishing carbocyclic nucleoside analogues in a one-pot operation.

## 

Carbocyclic nucleosides represent an emerging class of privileged therapeutic compounds which exhibit interesting biological properties.^1^,^2^ Substitution of endocyclic oxygen by a methylene unit in a carbocyclic nucleoside derivative imparts greater stability in the C–N pseudoglycosidic bond towards both chemical and enzymatic processes as compared to the parent nucleoside counterpart.^2^ Some examples of naturally-occurring and synthetic carbocyclic nucleosides with potent pharmacological activity, such as aristeromycin,^3^ neplanocin A,^4^ carbovir^5^ and abacavir,^6^ are depicted in Fig. 1.

**Fig. 1:**
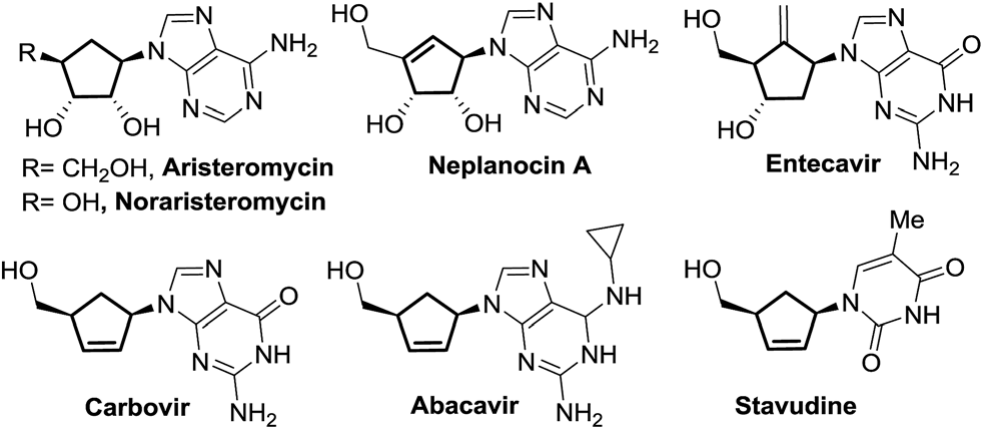
Biologically active carbocyclic nucleoside analogues.

Nazarov cyclization involving 4π electrocyclic ring closure of the pentadienyl cation has long been regarded as a versatile method to construct five-membered carbocycles which are ubiquitous in nature.^7^ In the classical Nazarov reaction, a variety of nucleophiles have been utilized to ingeniously intercept the cyclopentenyl cation formed following cyclization in inter- or intramolecular fashion, allowing rapid access to more elaborate cyclopentanoid compounds.^8^ The imino variant of the Nazarov reaction^9^ and the related intercepted process,9*f* in contrast, have not been exploited to a great extent due to unfavourable energetics of the ring closure reaction.^10^

Our previous success in the development of efficient domino processes involving intramolecular imino-Nazarov cyclization of the 1-aminopentadienyl cation prompted us to explore the fundamentally similar intermolecular capture of the transient oxyallyl cation intermediate.9*h* In particular, we became interested in the prospect of employing a silylated nucleobase as the intermolecular trapping partner to render formation of the nucleobase-containing cyclopentanone, which can be potentially transformed into carbocyclic nucleoside analogues (Scheme 1). It is notable that nitrogen nucleophiles have not been widely used in interrupted Nazarov reactions, presumably a consequence of the incompatibility of basic nitrogen-based nucleophiles with a highly acidic promoter, which is often necessary to effect cyclization. Tius reported a silica gel-/florisil-promoted amine intercepted Nazarov reaction,^11^ whereas West utilized organoazides as viable N-nucleophiles to trap the oxyallyl cation^12^ (Scheme 1). The present work described herein represents the first example in which a silylated nucleobase serves as an effective trapping agent for the oxyallyl cation in Nazarov-type cyclization.

**Scheme 1:**
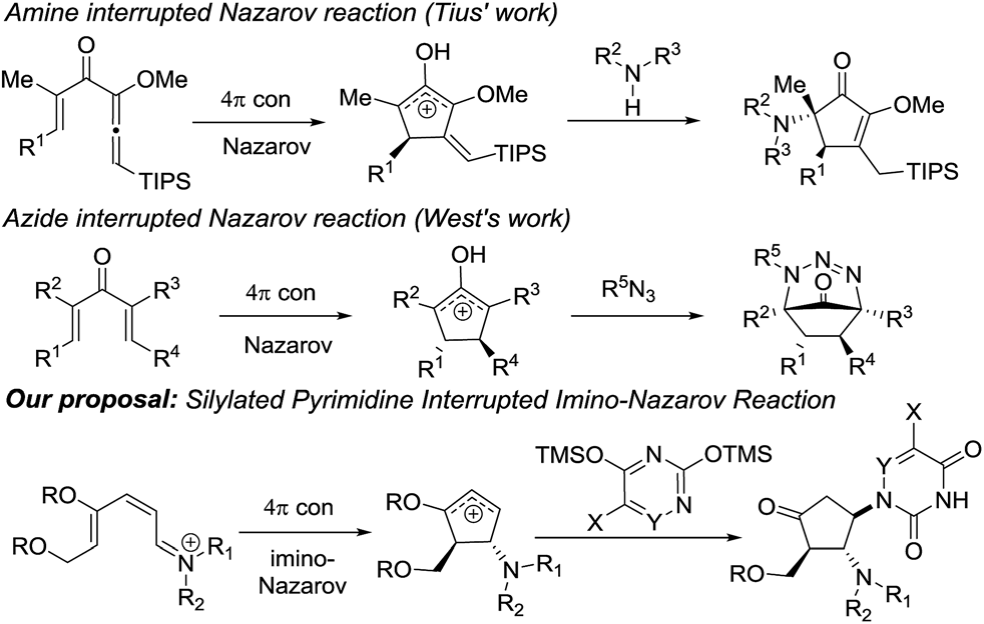
N-nucleophiles in interrupted Nazarov reactions.

Our initial efforts focused on the treatment of 4,6-dimethoxyhexa-2,4-dienal **1a**, *N*-benzyl-4-methoxyaniline **2a** and *O*,*O*′-bis(trimethylsilyl)thymine **3a** in the presence of a Lewis acid catalyst. The requisite 4,6-dimethoxyhexa-2,4-dienal **1a** could be obtained readily from 3,4,5-tri-*O*-methyl-d-glucal as an inseparable mixture of 2*Z*, 4*Z* and 2*Z*, 4*E* isomers in a 5 : 4 ratio, as described previously.9*h* We began our investigation with SnCl_4_ as the choice of Lewis acid catalyst as it is commonly used to activate silylated nucleobases in the Vorbrüggen method for preparation of nucleosides.^13^ To our delight, when the reaction was carried out in the presence of 30 mol% of SnCl_4_ in acetonitrile at 25 °C, dienal **1a** was fully consumed within an hour. Instead of the expected cyclopentanone product, however, the corresponding highly functionalized cyclic enol ether **4a** was isolated in 63% yield (Table 1, entry 1). Structural elucidation and stereochemical determination of compound **4a** was accomplished based on extensive 2D NMR (COSY and NOESY) studies. On the basis of the NOESY experiment, the absence of an observed correlation between protons at C-4 and C-5 indicates a *trans* configuration between the substituents. Additionally, a strong correlation between the protons of benzylic methylene and the proton at C-3 suggests that nucleophilic attack of silylated thymine occurs *trans* to the amino group at C-4. As compound **4a** retained the enol ether functionality, possibly allowing introduction of an electrophile at the α carbon, we decided to pursue formation of this type of carbocyclic nucleoside.^14^

**Table 1:** Optimization of reaction conditions^*a*^

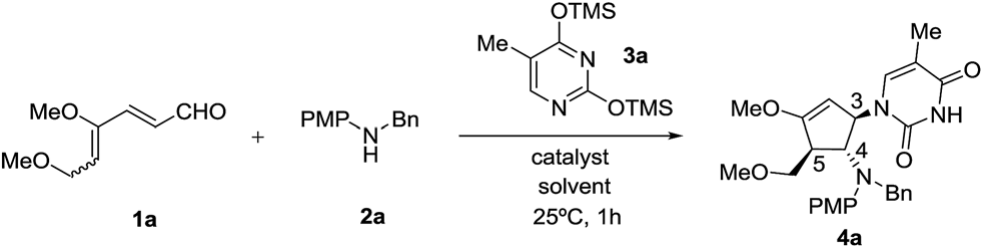			
Entry	Catalyst	Solvent	Yield^*b*^ (%)
1	SnCl_4_	CH_3_CN	63
2	TiCl_4_	CH_3_CN	57
3	Cu(OTf)_2_	CH_3_CN	52
4	FeBr_3_	CH_3_CN	58
5	InBr_3_	CH_3_CN	93
6	In(OTf)_3_	CH_3_CN	71
7	InBr_3_	CH_2_Cl_2_	79
8	InBr_3_	THF	65
9	InBr_3_	DMF	—
10^*c*^	InBr_3_	CH_3_CN	61

^*a*^Unless otherwise noted, reactions were performed using dienal **1a** (0.1 mmol, 1 equiv.), aniline **2a** (0.1 mmol, 1 equiv.) and silylated thymine **3a** (0.2 mmol, 2 equiv.) with 30 mol% of catalyst in 1 mL of solvent.

^*b*^NMR yield determined by ^1^H NMR analysis of the crude reaction against CH_2_Br_2_ as an internal standard.

^*c*^Reaction was carried out in the presence of 20 mol% of InBr_3_. PMP = *p*-methoxyphenyl.

Encouraged by this satisfactory initial result, we directed our attention to the investigation of a series of Lewis acids including TiCl_4_, Cu(OTf)_2_, FeBr_3_, InBr_3_ and In(OTf)_3_ in acetonitrile as the solvent. Although all tested Lewis acids were able to promote the desired intermolecular interrupted imino-Nazarov reaction, InBr_3_ emerged as the best catalyst, affording **4a** in 93% yield diastereoselectively (Table 1, entries 2–6). The reaction gave a lower yield when carried out in CH_2_Cl_2_ or THF (79% and 65% respectively), whereas no desired product **4a** was observed in DMF (Table 1, entries 7–9). An attempt to perform the present domino reaction using a lower loading of InBr_3_ (20 mol%) led to a diminished yield of **4a** (Table 1, entry 10).

With the optimal conditions in hand, we set out to demonstrate the general utility of this transformation by varying the silylated pyrimidine derivatives employed to furnish a library of carbocyclic nucleoside analogues **4b–4k** (Scheme 2). Simple unsubstituted silylated uracil furnished the cyclopentenol scaffold bearing a natural uracil group at C-3 **4b** in 91% yield. In general, substitution at the 5-position of silylated pyrimidine nucleophiles is tolerated in the present intermolecular interrupted imino-Nazarov reaction. Electron-withdrawing halogen substituents did not significantly influence the reactivity of silylated pyrimidine in trapping the oxyallyl cation, affording the desired product **4d–4g** in yields ranging from 87–91%. When silylated uracil derivatives with strongly electron-withdrawing groups such as –CF_3_ and –NO_2_ at the 5-position were used, the efficiency of the trapping process diminished slightly, giving 79% and 81% yield of **4h** and **4i**, respectively. Finally, modified uracil derivatives with more distinct variation in the core skeleton, including silylated 2-thiouracil and 5-azauracil, successfully participate in the reactions to provide **4j** and **4k** in good yields.

**Scheme 2:**
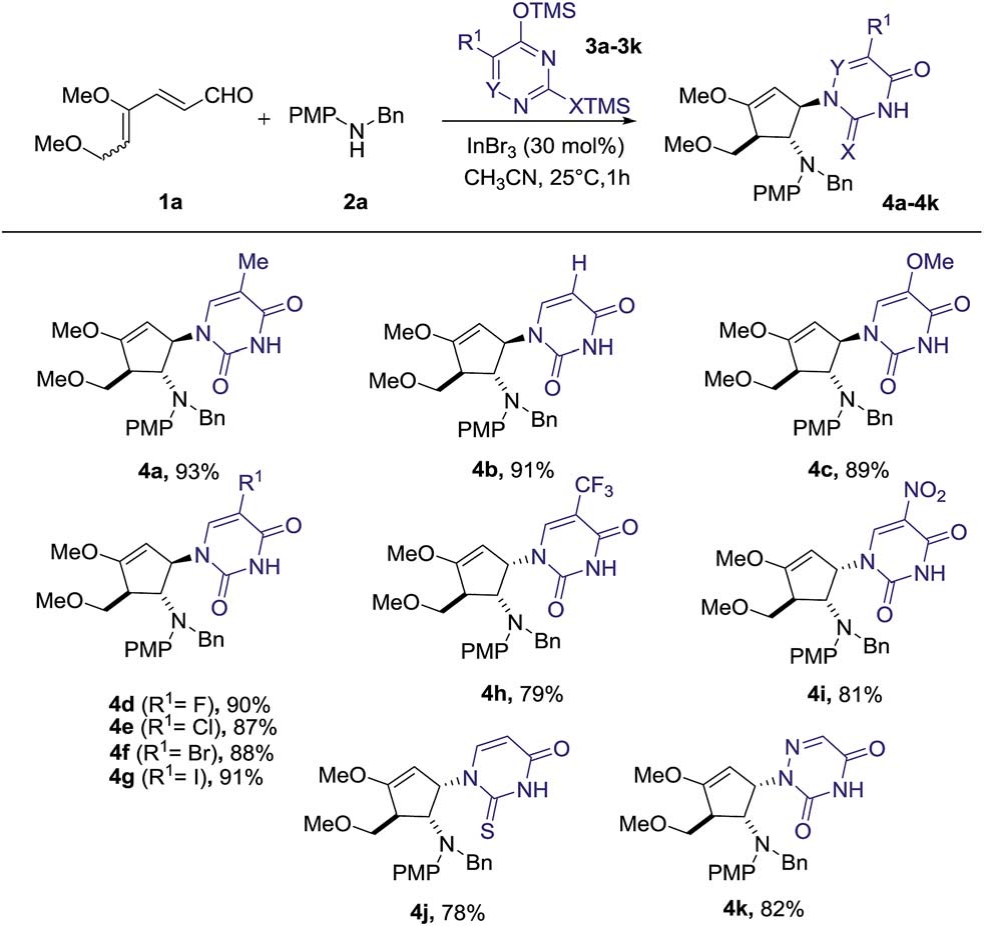
Scope of silylated uracil derivatives.

Having established the scope of silylated pyrimidine, we then directed our attention to examine the applicability of this reaction to a number of secondary anilines (Scheme 3). Various substituents including methyl, allyl, propargyl and 4-methoxybenzyl groups on the aniline nitrogen were well-tolerated, allowing formation of the carbocyclic nucleoside-like compounds **4l–4o**. A more electron-deficient aniline with a –Cl group at the *para* position of the aniline ring resulted in a moderate yield of 56% of the intercepted product **4p**.

**Scheme 3:**
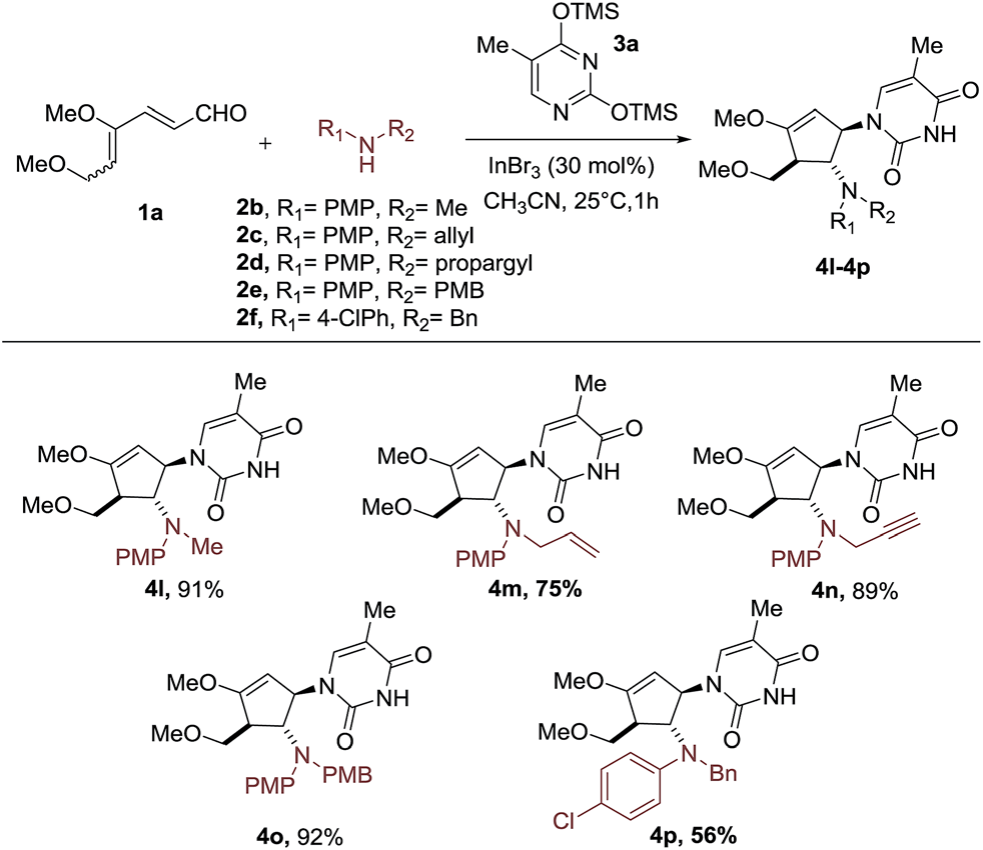
Scope of secondary anilines.

Subsequently, we proceeded to perform the reaction starting with different dienals which can similarly be derived from the corresponding glycals (Scheme 4). During the preparation of benzyl- and MOM-protected dienal **1b** and **1c**, as well as dienal **1d** derived from l-rhamnal, we found that the geometrical isomers (2*Z*, 4*Z* and 2*Z*, 4*E*) turn out to be separable, in contrast to the methyl protected counterpart **1a**. The reactions with these dienals were thus carried out using the pure *Z*-isomers instead of a mixture of isomers. When the benzyl protected dienal **1b** was subjected to the same reaction conditions, the interruption of the imino-Nazarov reaction proceeded smoothly to give an excellent yield of **4q** (Scheme 4). In the case of the more labile methoxymethyl protected dienal **1c**, the reaction still led to the formation of **4r**, albeit in a much lower yield of 52%, along with a substantial amount of 4-aminocyclopentenone (Scheme 4).^15^ It is notable that the presence of alkoxy (–OMe/–OBn) or methoxymethyl (–OMOM) groups is imperative for the success of this transformation, due to stabilization of the allyl cation intermediate.

**Scheme 4:**
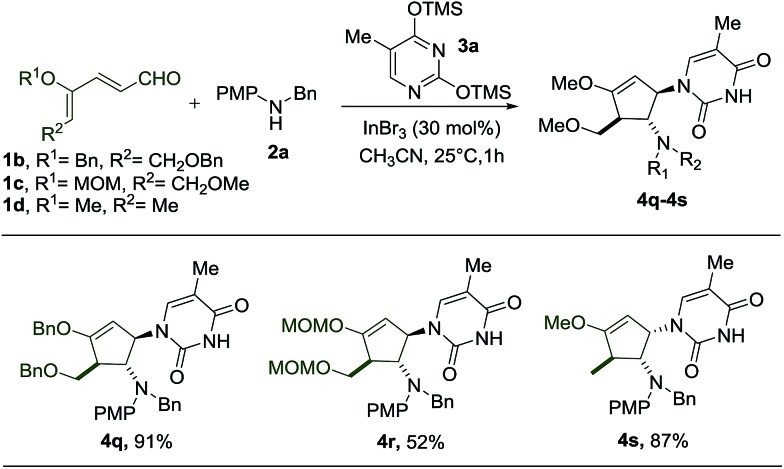
Scope of glycal-derived dienals.

To test our hypothesis that isomerization occurs prior to cyclization, we treated the 4*Z* isomer **1b** and 4*E* isomer **1b′** separately with aniline **2a** and silylated thymine **3a** under the same reaction conditions (Scheme 5). As expected, both isomers led to the formation of the same diastereomeric product **4q**, indicating that the initial cyclization provided only the *trans* oxyallyl cation intermediate, a consequence of alkene isomerization.^16^

**Scheme 5:**
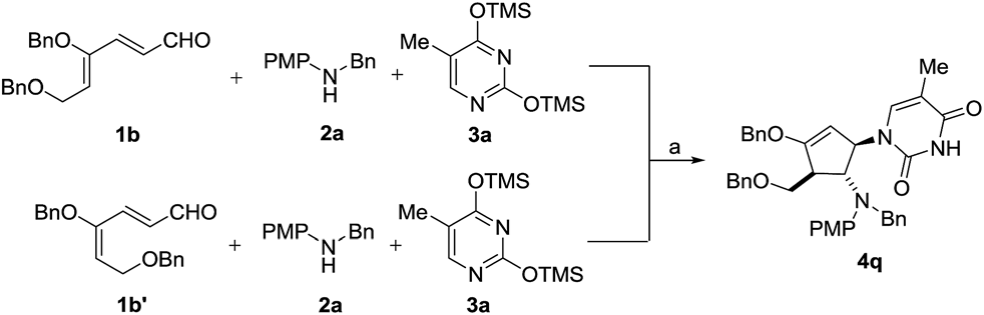
Stereoconvergence cyclization of **1b** and **1b′**. Reaction conditions: (a) 30 mol% InBr_3_, CH_3_CN, 25 °C. Yield of **4q**: 91% from **1b** and 85% from **1b′**.

It is noteworthy that, in all cases, the reactions exhibited excellent diastereoselectivity with regard to the nucleophilic addition of silylated pyrimidine to the oxyallyl cation species. To confirm that the diastereofacial selectivity arises due to steric hindrance, a simple theoretical DFT calculation for the possible trajectory of addition to the oxyallyl cation was conducted (see Fig. S1 in ESI†). The results of our theoretical studies correlate well with the experimental observation of complete diastereoselectivity.

Besides silylated pyrimidine, trimethylsilyl azide proved to be a competent nucleophile in trapping the oxyallyl cation, giving adduct **6** as the only diastereomer in which the azido group was incorporated into the cyclopentanoid framework. The instability of compound **6** in silica gel prevented us from isolating it in its pure form. The ESI mass spectrum of the crude product with molecular ion at *m*/*z* (M^+^ + H) coupled with its ^1^H NMR analysis provided strong evidence for the formation of **6**. Sequential hydrogenation on Pd/C in methanol resulted in the reduction of azide and alkene, along with removal of the benzyl group, rendering conversion of compound **6** to diaminocyclopentane **7** with an overall yield of 84% for 2 steps (Scheme 6). The structure and stereochemical assignment were determined based on a combination of 1D and 2D (COSY and NOESY) NMR studies. To date, there is only a single documented example of azidation of the oxyallyl cation, reported by West, using Me_2_AlN_3_ as the azide source.8*e*

**Scheme 6:**
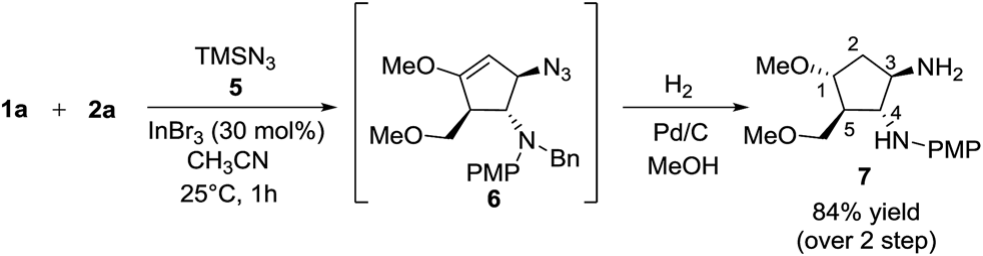
One-pot azide trapping/hydrogenation.

## Conclusions

In conclusion, we have developed the first examples of intermolecular interrupted imino-Nazarov reactions using silylated pyrimidine to provide convenient access to carbocyclic nucleoside analogues. The novel utility of using a silylated nucleobase as the nucleophilic trapping partner to intercept the fleeting oxyallyl cation from the imino-Nazarov reaction can potentially be extended to other variants of the Nazarov reaction.

## Supplementary Material

Supplementary information
