# Supplementary material for: The first intermolecular interrupted imino-Nazarov reaction: expeditious access to carbocyclic nucleoside analogues
Source: Chem Sci. 2015 Oct 27;7(2):1100–3. doi: 10.1039/c5sc03559g (PMC5954973; doi:10.1039/c5sc03559g)
Supplement: Supplementary file 1 [file SC-007-C5SC03559G-s001.pdf]

Electronic Supplementary Material (ESI) for Chemical Science.
This journal is © The Royal Society of Chemistry 2015

Supporting Information

Table of Contents

Table of Contents	Page
General Information	S2
Preparation of Dienal **1b (1b')**, **1c** and **1d**	S3
General Procedure for Preparation of *O*,*O*-Bis(trimethylsilyl)uracil Derivatives	S8
Representative Procedure for Silylated Pyrimidine Interrupted Imino-Nazarov	S9
Procedure for Synthesis of Diaminocyclopentane **7**	S9
Characterization Data of **4a-4s**	S11
Characterization Data of **7**	S22
1H, 13C and 2D (COSY and NOESY) NMR Spectra	S23
Computational Section	S58

General Information

Unless otherwise noted, all reactions were carried out in oven-dried glassware equipped with a magnetic stir bar under positive pressure of nitrogen. Anhydrous solvents were purchased from commercial suppliers and used without prior distillation with the exception of tetrahydrofuran (THF) which were distilled in the presence of sodium benzophenoneketyl. Analytical thin layer chromatography performed on Merck 60 F254 precoated silica gel (0.2 mm thickness). TLC plate visualization was achieved through irradiation with UV light at 254 nm and/or by staining the plate with either ceric ammonium molybdate solution or basic solution of potassium permanganate. Purification by flash column chromatography was carried out using silica gel 60 (0.010–0.063 mm) with eluents as noted in experimental data section for respective compounds. High Resolution Mass Spectroscopy (HRMS) spectra were recorded on a Waters Q-Tof premier™ mass Spectrometer. Proton and carbon NMR spectra were recorded at room temperature on Bruker DPX 400, and Bruker AMX 500 nuclear magnetic spectrometers. Chemical shifts for ^{1}H NMR spectra are reported as δ in units of parts per million (ppm) with reference to residual solvent peak of acetonirile (δ 7.26, singlet) or methanol (δ 3.34, singlet) and coupling constant (*J*) are reported in Hz. Multiplicities are reported as follows: s (singlet); brs (broad singlet); d (doublet); t (triplet); q (quartet); dd (doublet of doublet); ddd (doublet of doublet of doublet); dt (doublet of triplet); m (multiplet) and etc. Proton-decoupled carbon nuclear magnetic resonance spectra (^{13}C NMR) are reported as δ in units of parts per million as referenced to residual solvent peaks (acetonitrile: δ 1.79 and δ 118.26; methanol: δ 49.86). 4,6-dimethoxyhexa-2,4-dienal **1a** was prepared from glycal following literature reported procedure.[1] *N*-benzyl aniline derivatives **2a, 2e and 2f** were prepared via condensation of corresponding benzaldehyde and primary aniline followed by $NaBH_4$ reduction in methanol.[1] Secondary aniline **2b**,[2] **2c**,[3] and **2d**[4] were prepared following reported procedures.

Preparation of dienal 1b (1b'), 1c and 1d

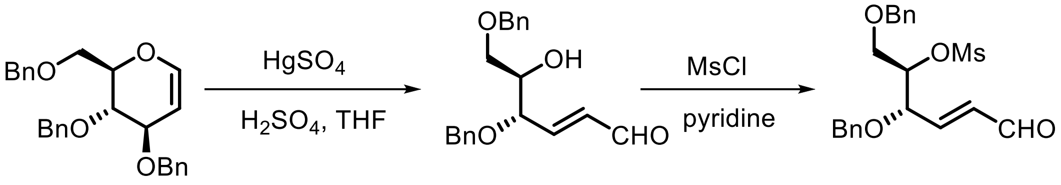

(2R,3S,E)-1,3-bis(benzyloxy)-6-oxohex-4-en-2-yl methanesulfonate:

To a solution of 3,4,6-tri-*O*-benzyl glucal (10.8g, 0.026 mol, 1 equiv) in THF (110 mL) was added 0.02N H_2SO_4 (220 mL) and catalytic amount of $HgSO_4$ (0.73 g, 266 mmol, 0.1 equiv). The reaction mixture was stirred at room temperature for 10 hours. Upon completion of the reaction, the reaction mixture was neutralized with excess barium carbonate and the resulting suspension was passed through a pad of Celite. The filtrate was extracted with CH_2Cl_2 (3 × 100 mL) and the combined organic layers was washed with brine (50 mL) and water (50 mL) successively, dried over Na_2SO_4 and evaporated under reduced pressure to give a colourless crude residue. Pyridine (50 mL) was added to the crude residue followed by MsCl (5.93 g, 0.052 mol, 2 equiv).The reaction mixture was stirred at ambient temperature for 30 min. The reaction mixture was diluted with ethyl acetate (200 mL) and washed with saturated aq. $CuSO_4$ solution until the blue $CuSO_4$ solution added stopped from turning into purple. The organic layer was then washed with water (2 × 50 mL), followed by brine (50 mL), dried over Na_2SO_4 and evaporated under reduced pressure. The crude residue was purified by flash column chromatography on silica gel (hexane/EtOAc 2:1) to afford mesylated enal (7.67 g, 73%) as colourless solid: ^1H NMR ($CDCl_3$, 400 MHz): δ 9.61 (d, *J*= 8.0 Hz, 1H), 7.34-7.45(m, 10H), 7.25 (d, *J*= 15.2 Hz, 1H), 6.67 (dd, *J*= 15.6, 8.0 Hz, 1H), 5.41 (t, *J*= 7.6 Hz, 1H), 4.93 (s, 2H), 4.59 (s, 2H), 4.28 (d, *J*= 7.6 Hz, 2H); ^{13}C NMR ($CDCl_3$, 100 MHz): δ 192.7, 150.2, 137.1, 136.7, 134.9, 128.6, 128.5, 128.3, 128.2, 128.0, 127.9, 81.2, 77.2, 73.6, 72.3, 68.1, 38.7; HRMS (ESI): *m/z* $[M+H]^+$ calcd for $C_{21}H_{25}O_6S$ 405.1372, found 405.1372.

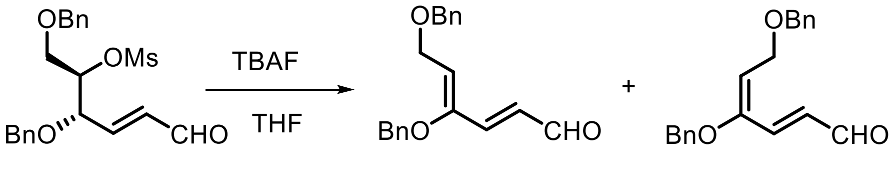

To a solution of mesylated benzyl-protected Perlin aldehyde (4.04 g, 0.010 mol, 1 equiv) in THF (50 mL) was added 1M TBAF in THF (20 mL, 0.020 mol, 2 equiv). The reaction mixture was stirred at room temperature. Upon completion of the reaction as monitored by TLC, the reaction was quenched by addition of water (50 mL). The resulting mixture was extracted with ethyl acetate (3 × 100 mL). The combined organic layers was washed with brine (50 mL), dried over Na_2SO_4 and evaporated under reduced pressure. The crude residue was purified by flash column chromatography on silica gel (hexane/EtOAc 4:1) to afford dienal (1.43 g, 88%) as mixture of 2E,4Z **1b** and 2E,4E **1b'** isomers in a 3:2 ratio**.**

(2E,4Z)-4,6-bis(benzyloxy)hexa-2,4-dienal (1b):

^{1}H NMR ($CDCl_3$, 400 MHz): δ 9.67 (d, *J*= 7.6 Hz, 1H), 7.33-7.41 (m, 10H), 6.95 (dd, *J*= 16.0, 5.6 Hz, 1H), 6.42 (dd, *J*= 15.6, 8.0 Hz, 1H), 5.80 (t, *J*= 6.8 Hz, 1H), 4.79 (s, 2H), 4.49 (s, 2H), 4.16 (d, *J*= 6.8 Hz, 2H); ^{13}C NMR ($CDCl_3$, 100 MHz): δ 193.2, 153.1, 147.5, 137.8, 136.4, 129.1, 128.6, 128.5, 128.2, 127.9, 127.8, 127.0, 126.2, 74.7, 72.8, 64.6; HRMS (ESI): *m/z* $[M+H]^+$ calcd for $C_{20}H_{21}O_3$ 309.1491, found 309.1491.

(2E,4E)-4,6-bis(benzyloxy)hexa-2,4-dienal (1b'):

^{1}H NMR ($CDCl_3$, 400 MHz): δ 961 (d, *J*= 7.6 Hz, 1H), 7.28-7.39 (m, 10H), 6.75 (dd, *J*= 16.0, 5.6 Hz, 1H), 6.35 (dd, *J*= 16.0, 8.0 Hz, 1H), 4.96 (dd, *J*= 10.8, 8.0 Hz, 1H), 4.64 (d, *J*= 11.6 Hz, 1H), 4.53-4.56 (m, 3H), 4.43-4.46 (m, 1H), 3.77 (dd, *J*= 10.8, 2.8 Hz, 1H), 3.67 (dd, *J*= 10.8, 4.8 Hz, 1H), 3.03 (s, 3H); ^{13}C NMR ($CDCl_3$, 100 MHz): δ 193.7, 153.0, 141.7, 137.9, 136.3, 130.0, 128.6, 128.5, 128.2, 128.1, 128.0, 127.9, 127.3, 107.4, 72.1, 69.5, 64.4; HRMS (ESI): *m/z* $[M+H]^+$ calcd for $C_{20}H_{21}O_3$ 309.1491, found 309.1491.

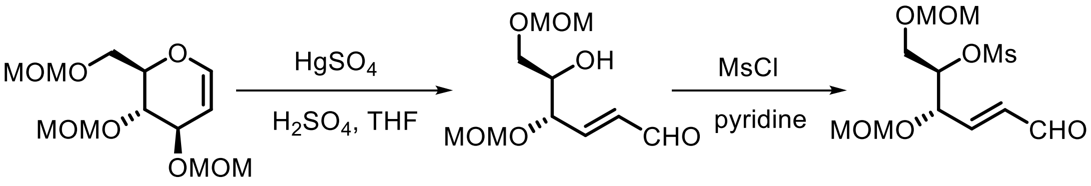

(5S,6R)-5-((E)-3-oxoprop-1-enyl)-2,4,8,10-tetraoxaundecan-6-yl methanesulfonate:

To a solution of 3,4,6-tri-*O*-methoxymethyl glucal (7.23 g, 0.026 mol, 1 equiv) in THF (110 mL) was added 0.02N H_2SO_4 (220 mL) and catalytic amount of $HgSO_4$ (0.73 g, 266 mmol, 0.1 equiv). The reaction mixture was stirred at room temperature for 10 hours. Upon completion of the reaction, the reaction mixture was neutralized with excess barium carbonate and the resulting suspension was passed through a pad of Celite. The filtrate was extracted with CH_2Cl_2 (3 × 100 mL) and the combined organic layers was washed with brine (50 mL) and water (50 mL) successively, dried over Na_2SO_4 and evaporated under reduced pressure to give a colourless crude residue. Pyridine (50 mL) was added to the crude residue followed by MsCl (5.93 g, 0.052 mol, 2 equiv).The reaction mixture was stirred at ambient temperature for 30 min. The reaction mixture was diluted with ethyl acetate (200 mL) and washed with saturated aq. $CuSO_4$ solution until the blue $CuSO_4$ solution added stopped from turning into purple. The organic layer was then washed with water (2 × 50 mL), followed by brine (50 mL), dried over Na_2SO_4 and evaporated under reduced pressure. The crude residue was purified by flash column chromatography on silica gel (hexane/EtOAc 3:1) to afford mesylated enal (4.21 g, 52%) as colourless solid: ^{1}H NMR ($CDCl_3$, 500 MHz): δ 9.64 (dd, *J*= 7.6, 1.2 Hz, 1H), 6.77 (dd, *J*= 16.0, 6.0 Hz, 1H), 6.37 (dd, *J*= 15.6, 5.6 Hz, 1H), 4.90-4.94 (m, 1H), 4.65-4.72 (m, 5H), 3.41-3.82 (m, 2H), 3.39 (s, 3H), 3.38 (s, 3H), 3.12 (s, 3H); ^{13}C NMR ($CDCl_3$, 125 MHz): δ 192.6, 149.7, 34.8, 96.8, 95.3, 81.3, 74.5, 65.7, 56.2, 55.7, 38.7; HRMS (ESI): *m/z* $[M+H]^+$ calcd for $C_{11}H_{21}O_8S$ 313.0957, found 313.0958.

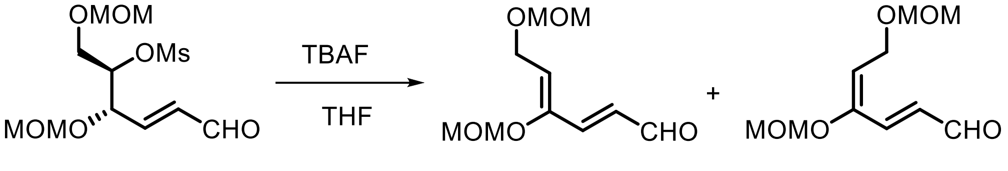

1c **1c'**

To a solution of mesylated MOM-protected Perlin aldehyde (3.12 g, 0.010 mol, 1 equiv) in THF (50 mL) was added 1M TBAF in THF (20 mL, 0.020 mol, 2 equiv). The reaction mixture was stirred at room temperature. Upon completion of the reaction as monitored by TLC, the reaction was quenched by addition of water (50 mL). The resulting mixture was extracted with ethyl acetate (3 × 100 mL). The combined organic layers was washed with brine (50 mL), dried over Na_2SO_4 and evaporated under reduced pressure. The crude residue was purified by flash column chromatography on silica gel (hexane/EtOAc 5:1) to afford dienal (1.87 g, 80%) as mixture of 2E,4Z **1c** and 2E,4E **1c’** isomers in a 4:3 ratio

(2E,4Z)-4,6-bis(methoxymethoxy)hexa-2,4-dienal (1c):

^{1}H NMR ($CDCl_3$, 400 MHz): δ 9.62-9.65 (m, 1H), 6.95 (d, *J*= 15.6 Hz, 1H), 6.37 (dd, *J*= 15.2, 7.6 Hz, 1H), 5.75 (t, *J*= 6.4 Hz, 1H), 4.89 (s, 2H), 4.67 (s, 2H), 4.34 (d, *J*= 6.4 Hz, 2H), 3.55 (s, 3H), 3.40 (s, 3H); ^{13}C NMR ($CDCl_3$, 100 MHz): δ 193.1, 152.0, 147.9, 129.1, 125.4, 98.1, 96.2, 62.2, 57.4, 55.4; HRMS (ESI): *m/z* $[M+H]^+$ calcd for $C_{10}H_{17}O_5$ 217.1076, found 217.1076.

(2E,4E)-4,6-bis(methoxymethoxy)hexa-2,4-dienal (1c’):

^{1}H NMR ($CDCl_3$, 400 MHz): δ 9.71 (d, *J*= 8.0 Hz, 1H), 7.33 (dd, *J*= 15.5, 6.0 Hz, 1H), 6.59 (dd, *J*= 15.0, 8.0 Hz, 1H), 5.60 (t, *J*= 8.0 Hz, 1H), 5.09 (s, 2H), 4.69 (s, 2H), 4.31 (d, *J*= 8.0 Hz, 2H), 3.47 (s, 3H), 3.42 (s, 3H); ^{13}C NMR ($CDCl_3$, 100 MHz): δ 193.6, 151.7, 141.4, 129.9, 110.4, 95.4, 94.1, 61.7, 56.3, 55.5; HRMS (ESI): *m/z* $[M+H]^+$ calcd for $C_{10}H_{17}O_5$ 217.1076, found 217.1076.

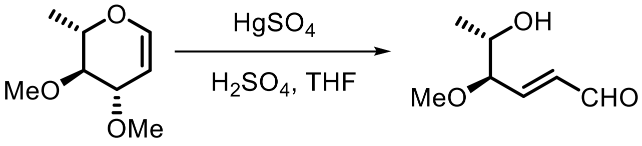

(4R,5S,E)-5-hydroxy-4-methoxyhex-2-enal:

To a solution of 3,4,6-tri-*O*-benzyl glucal (410 mg, 2.6 mmol, 1 equiv) in THF (11 mL) was added 0.02N H_2SO_4 (22 mL) and catalytic amount of $HgSO_4$ (73 mg, 2.6 mmol, 0.1 equiv). The reaction mixture was stirred at room temperature for 10 hours. Upon completion of the reaction, the reaction

mixture was neutralized with excess barium carbonate and the resulting suspension was passed through a pad of Celite. The filtrate was extracted with CH_2Cl_2 (3 × 10 mL) and the combined organic layers was washed with brine (5 mL) and water (5 mL) successively, dried over Na_2SO_4 and evaporated under reduced pressure to give a colourless crude residue. The crude residue was purified by flash column chromatography on silica gel (hexane/EtOAc 1:2) to afford hydroxyl enal (258 mg, 69%) as colourless liquid: ^{1}H NMR ($CDCl_3$, 500 MHz): δ 9.61 (d, *J*= 7.5 Hz, 1H), 6.78 (dd, *J*= 16.0, 6.5 Hz, 1H), 6.30 (ddd, *J*= 16.0, 8.0, 1.0 Hz, 1H), 3.99-4.02 (m, 1H), 3.39 (s, 3H), 2.48 (d, *J*= 5.0 Hz, 1H), 1.16 (d, *J*= 6.5 Hz, 3H); ^{13}C NMR ($CDCl_3$,125 MHz): δ 193.1, 152.5, 134.7, 84.5, 68.9, 57.7, 18.0; HRMS (ESI): *m/z* $[M+H]^+$ calcd for $C_7H_{13}O_3$ 145.0865, found 145.0863.

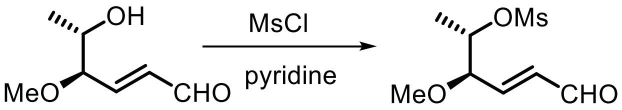

(2S,3R,E)-3-methoxy-6-oxohex-4-en-2-yl methanesulfonate:

To a solution of Perlin aldehyde **7** (172 mg, 1.2 mmol, 1 equiv) in pyridine (2 mL) was added MsCl (185 µL, 2.4 mmol, 2 equiv). The reaction mixture was stirred at ambient temperature for 30 min. The reaction mixture was diluted with ethyl acetate (20 mL) and washed with saturated aq. $CuSO_4$ solution until the blue $CuSO_4$ solution added stopped from turning into purple. The organic layer was then washed with water (2 × 5 mL), followed by brine (5 mL), dried over Na_2SO_4 and evaporated under reduced pressure. The crude residue was purified by flash column chromatography on silica gel (hexane/EtOAc 2:1) to afford mesylated enal (266 mg, 98%) as colourless solid: ^{1}H NMR ($CDCl_3$, 500 MHz): δ 9.64 (d, *J*= 7.5 Hz, 1H), 6.71 (dd, *J*= 16.0, 6.5 Hz, 1H), 6.35 (ddd, *J*= 16.0, 8.0, 1.0 Hz, 1H), 4.91-4.96 (m, 1H), 3.98 (ddd, *J*= 6.5, 3.5, 1.0 Hz, 1H), 3.42 (s, 3H), 3.07 (s, 3H), 1.39 (d, *J*= 6.5 Hz, 3H); ^{13}C NMR ($CDCl_3$, 125 MHz): δ 192.7, 149.7, 135.5, 82.5, 79.0, 58.0, 38.7, 17.0; HRMS (ESI): *m/z* $[M+H]^+$ calcd for $C_8H_{15}O_5S$ 223.0640, found 223.0640.

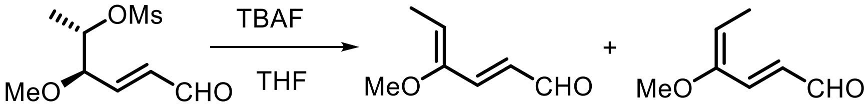

1d **1d'**

To a solution of mesyl-protected Perlin aldehyde **8** (222 mg, 1.0 mmol, 1 equiv) in THF (5 mL) was added 1M TBAF in THF (2 mL, 2.0 mol, 2 equiv). The reaction mixture was stirred at room temperature. Upon completion of the reaction as monitored by TLC, the reaction was quenched by addition of water (5 mL). The resulting mixture was extracted with ethyl acetate (3 × 10 mL). The combined organic layers was washed with brine (5 mL), dried over Na_2SO_4 and evaporated under reduced pressure. The crude residue was purified by flash column chromatography on silica gel (hexane/EtOAc 4:1) to afford dienal (113 mg, 90%) as mixture of 2E,4Z **1d** and 2E,4E **1d'** isomers in a 5:4 ratio.

(2E,4Z)-4-methoxyhexa-2,4-dienal (1d):

^{1}H NMR ($CDCl_3$, 400 MHz): δ 9.61 (d, *J*= 8.4 Hz, 1H), 6.86 (d, *J*= 15.6 Hz, 1H), 6.29 (dd, *J*= 15.6, 8.0 Hz, 1H), 5.64 (dd, *J*= 15.6, 7.2 Hz, 1H), 3.65 (s, 3H), 1.86 (d, *J*= 7.6 Hz, 3H); ^{13}C NMR ($CDCl_3$, 100 MHz): δ 193.5, 154.5, 148.4, 126.9, 125.8, 59.7, 12.0; HRMS (ESI): *m/z* $[M+H]^+$ calcd for $C_7H_{11}O_2$ 127.0759, found 127.0759.

(2E,4E)-4-methoxyhexa-2,4-dienal (1d'):

^{1}H NMR ($CDCl_3$, 400 MHz): δ 9.67 (d, *J*= 8.0 Hz, 1H), 7.33 (d, *J*= 15.6 Hz, 1H), 6.49 (dd, *J*= 15.2, 8.0 Hz, 1H), 5.19 (dd, *J*= 14.8, 7.2 Hz, 1H), 3.63 (s, 3H), 1.89 (d, *J*= 3.2 Hz, 3H); ^{13}C NMR ($CDCl_3$, 100 MHz): δ 193.9, 151.3, 142.0, 128.3, 106.5, 54.7, 12.1; HRMS (ESI): *m/z* $[M+H]^+$ calcd for $C_7H_{11}O_2$ 127.0759, found 127.0759.

General Procedure for Preparation of *O*,*O*-Bis(trimethylsilyl)uracil Derivatives[5]

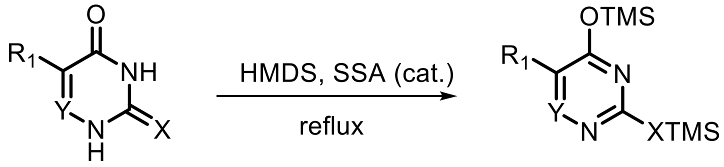

3b-3k

In an oven-dried round-bottom flask, a mixture of pyrimidine derivatives (10 mmol, 1 equiv), SSA (0.05 g), and HMDS (100 mL) was stirred at reflux until the solution became clear, typically after 0.5-2h. The catalyst was then filtered off and the filtrate was subjected to rotary evaporator under vacuum

to remove HMDS. The resulting crude product was sufficiently pure to be used in interrupted imino-Nazarov reaction without further purification.

Representative Procedure for Silylated Pyrimidine Interrupted Imino-Nazarov

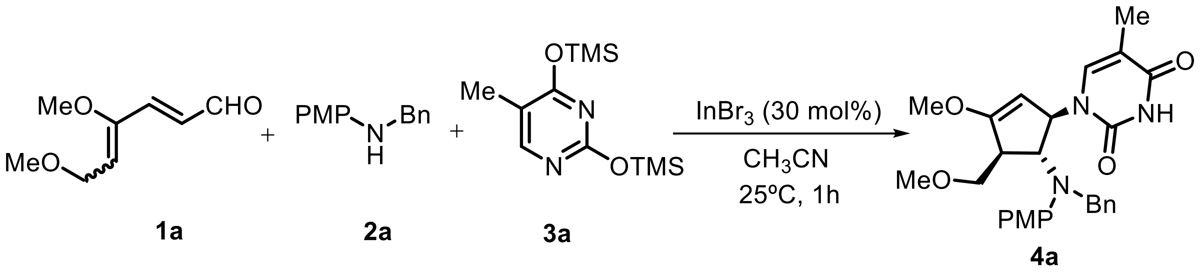

In an oven-dried round-bottomed flask, 4,6-dimethoxyhexa-2,4-dienal **1a** (15.6 mg, 0.1 mmol, 1 equiv) was dissolved in anhydrous MeCN (1 mL). To the resulting solution was added *N*-benzyl 4-methoxyaniline **2a** (21.3 mg, 0.1 mmol, 1 equiv) and O,O-bis(trimethylsilyl)thymine **3a** (51.1 mg, 0.2 mmol, 2 equiv) followed by $InBr_3$ (10.6 mg, 0.03 mmol, 0.3 equiv). The reaction mixture was then allowed to stir at ambient temperature for 1 hour. Upon complete consumption of starting materials, the reaction was subsequently quenched by addition of $NaHCO_3$ (2 mL). The resulting mixture was extracted with EtOAc (3 × 5 mL). The combined organic layers was washed with brine (5 mL), dried over Na_2SO_4, filtered and concentrated under reduced pressure to afford the crude residue. The crude residue is subjected to flash column chromatography on silica gel (CH_2Cl_2/MeOH) to give pure compound **4a**.

Procedure for Synthesis of Diaminocyclopentane 7

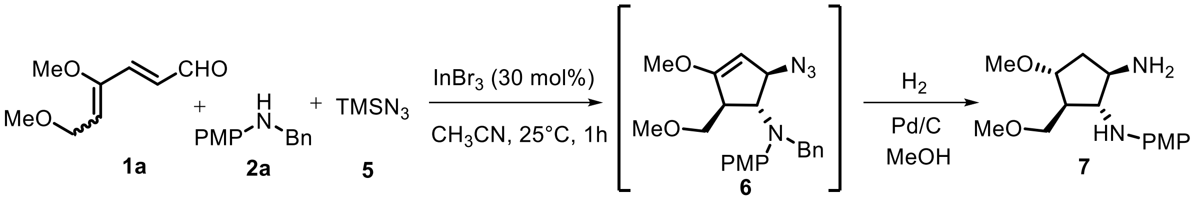

In an oven-dried round-bottomed flask, 4,6-dimethoxyhexa-2,4-dienal **1a** (15.6 mg, 0.1 mmol, 1 equiv) was dissolved in anhydrous MeCN (1 mL). To the resulting solution was added *N*-substituted aniline **2a** (21.3 mg, 0.1 mmol, 1 equiv) and trimethylsilyl azide **5** (23.0 mg, 0.12 mmol, 2 equiv) followed by

$InBr_3$ (10.6 mg, 0.03 mmol, 0.3 equiv). The reaction mixture was then allowed to stir at ambient temperature for 1 hour. Upon complete consumption of starting materials, the reaction was subsequently quenched by addition of $NaHCO_3$ (2 mL). The resulting mixture was extracted with EtOAc (3 × 5 mL). The combined organic layers was washed with brine (5 mL), dried over Na_2SO_4, filtered and concentrated under reduced pressure to afford the crude residue. The crude residue was treated with 10% Pd on carbon (10 mg) in MeOH at room temperature under H_2 atmosphere. Upon completion of reaction as monitor by TLC, the reaction mixture was filtered over a short pad of celite. The filtrate was concentrated under reduced pressure and purified by column chromatography on silica gel (hexane/EtOAc) affording pure compound **7**.

References:

1) R. William, S. Wang, F. Ding, E. N. Arviana, X.-W. Liu, *Angew. Chem. Int. Ed.* **2014**, *53*, 10742-10746.

2) R. J. Lundgren, A. Sappong-Kumankumah, M. Stradiotto, *Chem. Eur. J.*, **2010**, *16,* 1983-1991.

3) L. Adak, K. Chattopadhyay, B. C. Ranu, *J. Org. Chem*. **2009**, *74*, 3982-3985.

4) K. C. Majumdar, S. Ganai, *Tetrahedron Lett.* **2013**, *54,* 6192-6195.

5) M. N. S. Rad, A. Khalafi-Nezhad, M. Divar, S. Behrouz, *Phosphorus, Sulfur Silicon Relat. Elem.,* **2010**, *185*, 1943-1954.

Characterization Data of 4a-4s

1-(5-(benzyl(4-methoxyphenyl)amino)-3-methoxy-4-(methoxymethyl)cyclopent-2-enyl)-5-methylpyrimidine-2,4(1H,3H)-dione (4a):

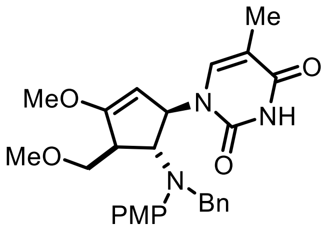

4a

Reaction was carried out according to representative procedure described above. Flash column chromatography (hexane/EtOAc 1:1) gave **4a** as yellow oil (44.4 mg, 93%): ^{1}H NMR (CD_3CN, 400 MHz): δ 9.47 (s, 1H), 7.35 (s, 1H), 7.33-7.35 (m, 2H), 7.24-7.32 (m, 2H), 7.17-7.21 (m, 1H), 6.79-6.82 (m, 2H), 6.71-6.75 (m, 2H), 5.85-5.87 (m, 1H), 4.51 (s, 1H), 4.52 (s, 2H), 4.14 (dd, *J*= 6.4, 5.6 Hz, 1H), 3.70 (dd, *J*= 9.6, 3.2 Hz, 1H), 3.68 (s, 3H), 3.67 (s, 3H), 3.43 (dd, *J*= 9.6, 2.8 Hz, 1H), 3.33 (s, 3H), 2.94-2.96 (m, 1H), 1.80 (d, *J*= 0.8 Hz, 3H); ^{13}C NMR (CD_3CN, 100 MHz): δ 163.9, 162.2, 153.2, 151.3, 142.2, 139.6, 137.3, 128.3, 127.6, 126.8, 118.9, 114.3, 110.0, 94.1, 69.9, 68.6, 59.6, 58.4, 56.6, 55.0, 52.9, 46.5, 11.6; HRMS (ESI): *m/z* $[M+H]^+$ calcd for $C_{27}H_{32}N_3O_5$ 478.2342, found 478.2341.

1-(5-(benzyl(4-methoxyphenyl)amino)-3-methoxy-4-(methoxymethyl)cyclopent-2-enyl)pyrimidine-2,4(1H,3H)-dione (4b):

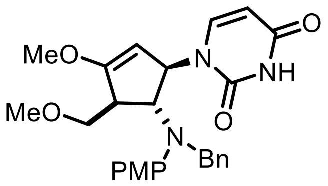

4b

Reaction was carried out according to general procedure described above. Flash column chromatography (hexane/EtOAc 1:1) gave **4b** as colourless oil (42.2 mg, 91%): ^{1}H NMR (CD_3CN , 400 MHz): δ 9.21 (s, 1H), 7.52 (d, *J*= 8.0 Hz, 1H), 7.34 (d, *J*= 7.2 Hz, 2H), 7.27 (t, *J*= 7.6 Hz, 2H), 7.20 (d, *J*= 7.2 Hz, 1H), 6.78-6.82 (m, 2H), 6.71-6.75 (m, 2H), 5.85-5.86 (m, 1H), 5.57 (d, *J*= 8.0 Hz, 1H), 4.51 (s, 1H), 4.43 (s, 2H), 4.15 (dd, *J*= 6.8, 5.6 Hz, 1H), 3.67-3.70 (m, 7H), 3.42 (dd, *J*= 9.6, 2.8 Hz,

1H), 3.31 (s, 3H), 2.94-2.96 (m, 1H); ^{13}C NMR (CD_3CN, 100 MHz): δ 163.2, 162.5, 153.2, 151.2, 142.2, 141.5, 139.7, 128.3, 127.5, 126.8, 118.9, 114.2, 101.6, 93.8, 69.7, 69.0, 59.9, 58.4, 56.7, 55.0, 52.5, 46.6; HRMS (ESI): *m/z* [M+H]$^+$ calcd for $C_{26}H_{30}N_3O_5$ 464.2185, found 464.2183.

1-((1S,4R,5S)-5-(benzyl(4-methoxyphenyl)amino)-3-methoxy-4-(methoxymethyl)cyclopent-2-enyl)-5-methoxypyrimidine-2,4(1H,3H)-dione (4c):

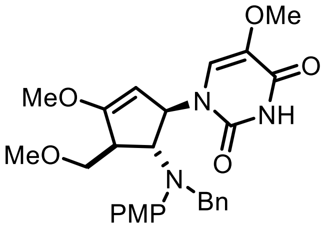

4c

Reaction was carried out according to general procedure described above. Flash column chromatography (hexane/EtOAc 1:1) gave **4c** as brown oil (43.9 mg, 89%): ^{1}H NMR (CD_3CN , 400 MHz): δ 9.29 (s, 1H), 7.32 (d, *J*= 6.8 Hz, 2H), 7.25-7.29 (m, 2H), 7.20-7.22 (m, 1H), 7.04 (s, 1H), 6.80-6.83 (m, 2H), 6.74-6.77 (m, 2H), 5.88-5.90 (m, 1H), 4.53 (s, 1H), 4.41 (s, 2H), 4.20 (dd, *J*= 6.4, 5.2 Hz, 1H), 3.69-3.72 (m, 7H), 3.62 (s, 3H), 3.41 (dd, *J*= 9.6, 2.4 Hz, 1H), 3.31 (s, 3H), 2.95-2.97 (m, 1H); ^{13}C NMR (CD_3CN, 100 MHz): δ 162.2, 159.1, 153.2, 149.6, 142.1, 139.6, 136.5, 128.3, 127.6, 126.8, 120.8, 118.8, 114.3, 94.1, 69.9, 67.5, 60.0, 58.6, 57.0, 56.7, 55.0, 53.4, 46.4; HRMS (ESI): *m/z* [M+H]$^+$ calcd for $C_{27}H_{32}N_3O_6$ 494.2291, found 494.2291.

1-(5-(benzyl(4-methoxyphenyl)amino)-3-methoxy-4-(methoxymethyl)cyclopent-2-enyl)-5-fluoropyrimidine-2,4(1H,3H)-dione (4d):

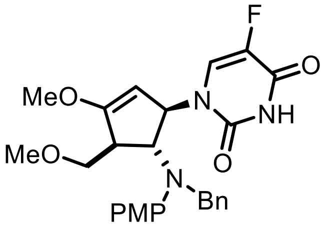

4d

Reaction was carried out according to general procedure described above. Flash column chromatography (hexane/EtOAc 1:1) gave **4d** as brown solid (43.3 mg, 90%): ^{1}H NMR (CD_3CN , 400 MHz): δ 9.48 (s, 1H), 7.68 (dd, *J*= 7.2, 0.8 Hz, 1H), 7.35 (d, *J*= 7.2 Hz, 2H), 7.27-7.31 (m, 2H), 7.19-7.23 (m,1H), 6.81-6.84 (m, 2H), 6.74-6.78 (m, 2H), 5.83-5.85 (m, 1H), 4.52 (s, 1H), 4.42 (s, 2H), 4.13 (t, *J*= 5.6 Hz, 1H), 3.70-3.73 (m, 7H), 3.41 (dd, *J*= 9.6, 2.8 Hz, 1H), 3.33 (s, 3H), 2.93-2.96 (m, 1H); ^{13}C NMR (CD_3CN, 100 MHz): δ 163.0, 157.1, 156.8, 153.3, 149.7, 142.1, 141.6, 139.6, 139.3, 128.3, 127.6, 126.8, 125.8, 125.5, 118.9, 114.3, 93.6, 70.0, 68.6, 60.9, 58.3, 56.8, 55.0, 52.9, 46.7; HRMS (ESI): *m/z* $[M+H]^+$ calcd for $C_{26}H_{29}FN_3O_5$ 482.2091, found 482.2091.

1-(5-(benzyl(4-methoxyphenyl)amino)-3-methoxy-4-(methoxymethyl)cyclopent-2-enyl)-5-chloropyrimidine-2,4(1H,3H)-dione (4e):

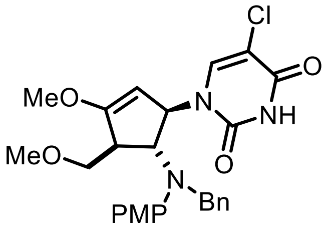

4e

Reaction was carried out according to general procedure described above. Flash column chromatography (hexane/EtOAc 1:1) gave **4e** as brown liquid (43.2 mg, 87%): ^{1}H NMR (CD_3CN , 400 MHz): δ 9.48 (s, 1H), 7.86 (s, 1H), 7.34 (d, *J*= 7.2 Hz, 2H), 7.27-7.31 (m, 2H), 7.22 (d, *J*= 7.2 Hz, 1H), 6.81-6.84 (m, 2H), 6.75-6.78 (m, 2H), 5.82-5.83 (m, 1H), 4.54 (s, 1H), 4.40 (s, 2H), 4.10 (dd, *J*= 5.6, 4.4 Hz, 1H), 3.70-3.74 (m, 7H), 3.38 (dd, *J*= 9.6, 2.8 Hz, 1H), 3.34 (s, 3H), 2.92-2.94 (m, 1H); ^{13}C NMR (CD_3CN, 100 MHz): δ 163.4, 159.0, 153.4, 150.2, 142.0, 139.6, 139.2, 128.3, 127.6, 126.9, 119.0, 114.3, 107.5, 93.9, 70.1, 68.7, 61.3, 58.4, 56.9, 55.1, 53.1, 46.9; HRMS (ESI): *m/z* $[M+H]^+$ calcd for $C_{26}H_{29}ClN_3O_5$ 498.1796, found 498.1794.

1-(5-(benzyl(4-methoxyphenyl)amino)-3-methoxy-4-(methoxymethyl)cyclopent-2-enyl)-5-bromopyrimidine-2,4(1H,3H)-dione (4f):

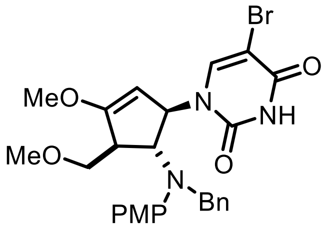

4f

Reaction was carried out according to general procedure described above. Flash column chromatography (hexane/EtOAc 1:1) gave **4f** as brown oil (47.7 mg, 88%): ^{1}H NMR (CD_3CN , 400 MHz): δ 9.54 (s, 1H), 7.97 (s, 1H), 7.34 (d, *J*= 7.2 Hz, 2H), 7.27-7.31 (m, 2H), 7.20-7.21 (m, 2H), 6.82-6.84 (m, 2H), 6.75-6.77 (m, 2H), 5.81-5.83 (m, 1H), 4.55 (s, 1H), 4.40 (s, 2H), 4.09 (t, *J*= 4.8 Hz, 1H), 3.73 (dd, *J*= 9.6, 2.4 Hz, 1H), 3.71 (s, 3H), 3. 70 (s, 3H), 3.37 (dd, *J*= 9.6, 2.4 Hz, 1H), 3.35 (s, 3H), 2.91-2.93 (m, 1H); ^{13}C NMR (CD_3CN, 100 MHz): δ 163.4, 159.2, 153.4, 150.5, 142.0, 139.6, 128.3, 127.6, 127.0, 118.9, 114.4, 95.4, 94.0, 70.1, 68.6, 61.3, 58.4, 56.9, 55.1, 53.2, 47.0; HRMS (ESI): *m/z* $[M+H]^+$ calcd for $C_{26}H_{29}BrN_3O_5$ 542.1291, found 542.1291.

1-(5-(benzyl(4-methoxyphenyl)amino)-3-methoxy-4-(methoxymethyl)cyclopent-2-enyl)-5-iodopyrimidine-2,4(1H,3H)-dione (4g):

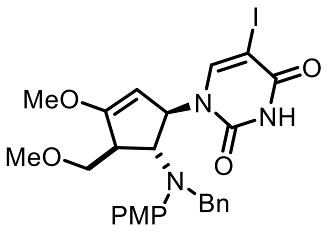

4g

Reaction was carried out according to general procedure described above. Flash column chromatography (hexane/EtOAc 1:1) gave **4g** as white solid (53.6 mg, 91%): ^{1}H NMR ($CDCl_3$, 400 MHz): δ 8.40 (s, 1H), 8.07 (s, 1H), 7.22-7.32 (m, 5H), 6.77-6.83 (m, 4H), 5.88 (t, *J*= 2.8 Hz, 1H), 4.44 (s, 1H), 4.40 (d, *J*= 15.6 Hz, 1H), 4.34 (d, *J*= 15.6 Hz, 1H), 4.05-4.08 (m, 1H), 3.73-3.77 (m, 7H), 3.42 (dd, *J*= 9.6, 2.8 Hz, 1H), 2.87-2.89 (m, 1H); ^{13}C NMR ($CDCl_3$, 100 MHz): δ 158.4, 154.6, 48.3, 145.0,

141.8, 133.6, 123.2, 122.2, 121.9, 113.9, 109.4, 88.6, 64.6, 62.2, 62.0, 56.2, 54.0, 51.9, 50.2, 49.3, 42.0; HRMS (ESI): *m/z* $[M+H]^+$ calcd for $C_{26}H_{29}IN_3O_5$ 590.1152, found 590.1151.

1-(5-(benzyl(4-methoxyphenyl)amino)-3-methoxy-4-(methoxymethyl)cyclopent-2-enyl)-5-(trifluoromethyl)pyrimidine-2,4(1H,3H)-dione (4h):

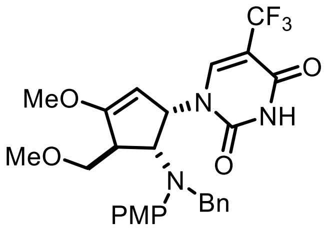

4h

Reaction was carried out according to general procedure described above. Flash column chromatography (hexane/EtOAc 1:1) gave **4h** as yellow oil (41.9 mg, 79%): ^{1}H NMR (CD_3CN , 400 MHz): δ 10.27 (s, 1H), 8.10 (s, 1H), 7.25 (d, *J*= 7.2 Hz, 2H), 7.16 (t, *J*= 7.2 Hz, 2H), 7.08-7.11 (m, 1H), 6.79 (d, *J*= 8.8 Hz, 2H), 6.66 (d, *J*= 9.2 Hz, 2H), 5.89 (s, 1H), 4.59 (s, 1H), 4.33 (s, 2H), 4.05 (t, *J*= 4.4 Hz, 1H), 3.62-3.63 (m, 4H), 3.59 (s, 3H), 3.27 (dd, *J*= 9.6, 2.0 Hz, 1H), 3.19 (s, 3H), 2.85 (s, 1H); ^{13}C NMR (CD_3CN, 100 MHz): δ 163.8, 158.3, 153.6, 150.1, 143.1, 143.0, 143.0, 141.7, 139.4, 128.2, 127.5, 126.7, 119.2, 114.2, 93.9, 70.0, 68.0, 61.5, 58.1, 56.7, 54.6, 53.9, 47.2; HRMS (ESI): *m/z* $[M+H]^+$ calcd for $C_{27}H_{29}F_3N_3O_5$ 532.2059, found 532.2061.

1-(5-(benzyl(4-methoxyphenyl)amino)-3-methoxy-4-(methoxymethyl)cyclopent-2-enyl)-5-nitropyrimidine-2,4(1H,3H)-dione (4i):

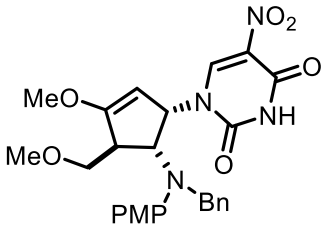

4i

Reaction was carried out according to general procedure described above. Flash column chromatography (hexane/EtOAc 1:1) gave **4i** as brown oil (41.2 mg, 81%): ^{1}H NMR (CD_3CN , 400

MHz): δ 9.38 (s, 1H), 9.12 (s, 1H), 7.36 (d, *J*= 7.2 Hz, 2H), 7.27-7.31 (m, 2H), 7.22 (t, *J*= 7.2 Hz, 2H), 6.82-6.86 (m, 2H), 6.75-6.80 (m, 2H), 5.86 (t, *J*= 2.4 Hz, 1H), 4.60 (d, *J*= 1.6 Hz, 1H), 4.40 (s, 2H), 4.17 (dd, *J*= 8.8, 4.0 Hz, 1H), 3.73-3.76 (m, 4H), 3.71 (s, 3H), 3.31-3.33 (m, 4H), 2.93-2.95 (s, 1H); ^{13}C NMR (CD_3CN, 100 MHz): δ 164.8, 154.2, 153.5, 149.3, 147.1, 141.7, 139.6, 128.4, 127.6, 126.9, 118.9, 114.4, 93.7, 70.1, 68.8, 63.3, 58.5, 57.1, 55.0, 52.9, 47.4; HRMS (ESI): *m/z* $[M+H]^+$ calcd for $C_{26}H_{29}N_4O_7$ 509.2036, found 509.2036.

1-(5-(benzyl(4-methoxyphenyl)amino)-3-methoxy-4-(methoxymethyl)cyclopent-2-enyl)-2-thioxo-2,3-dihydropyrimidin-4(1H)-one (4j):

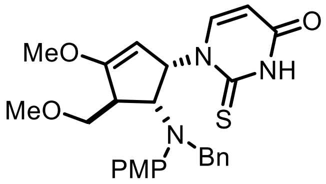

4j

Reaction was carried out according to general procedure described above. Flash column chromatography (hexane/EtOAc 1:1) gave **4j** as brown oil (37.4 mg, 78%): ^{1}H NMR (CD_3CN , 400 MHz): δ 10.xx (s, 1H), 7.65 (d, *J*= 8.4 Hz, 1H), 7.34 (d, *J*= 7.2 Hz, 2H), 7.27-7.30 (m, 2H), 7.21 (t, *J*= 7.2 Hz, 1H), 6.89-6.90 (m, 1H), 6.82-6.85 (m, 2H), 6.73-6.77 (m, 2H), 5.88 (d, *J*= 8.0 Hz, 1H), 4.57 (s, 1H), 4.52 (d, *J*= 16.0 Hz, 1H), 4.44 (d, *J*= 16.0 Hz, 1H), 4.17 (dd, *J*= 10.6, 6.4 Hz, 1H), 3.70-3.73 (m, 4H), 3.69 (s, 3H), 3.41 (dd, *J*= 9.6, 2.8 Hz, 1H), 3.32 (s, 3H), 2.97-2.99 (m, 1H); ^{13}C NMR (CD_3CN, 100 MHz): δ 176.8, 163.1, 153.3, 142.1, 141.8, 139.5, 128.3, 127.6, 126.8, 118.9, 114.2, 107.1, 94.1, 69.6, 68.3, 66.5, 58.4, 56.8, 55.0, 53.4, 47.0; HRMS (ESI): *m/z* $[M+H]^+$ calcd for $C_{26}H_{30}N_3O_4S$ 480.1957, found 480.1954.

2-(5-(benzyl(4-methoxyphenyl)amino)-3-methoxy-4-(methoxymethyl)cyclopent-2-enyl)-1,2,4-triazine-3,5(2H,4H)-dione (4k):

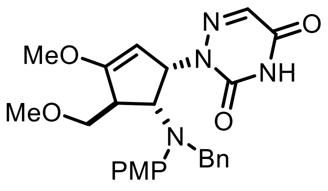

4k

Reaction was carried out according to general procedure described above. Flash column chromatography (hexane/EtOAc 1:1) gave **4k** as brown oil (38.1 mg, 82%): ^{1}H NMR ($CDCl_3$, 400 MHz): δ 9.34 (s, 1H), 7.17-7.30 (m, 6H), 6.80-6.83 (m, 2H), 6.72-6.75 (m, 2H), 5.97-5.99 (m, 1H), 4.63 (dd, *J*= 7.6, 6.8 Hz, 1H), 4.49 (d, *J*= 16.0 Hz, 1H), 4.38 (d, *J*= 16.0 Hz, 1H), 3.67-3.73 (m, 7H), 3.61 (dd, *J*= 9.6, 3.6 Hz, 1H), 3.43 (s, 3H), 3.01-3.05 (m, 1H); ^{13}C NMR (CD_3CN, 100 MHz): δ 161.1, 155.8, 153.0, 148.1, 142.4, 139.4, 135.3, 128.4, 127.4, 126.8, 118.5, 114.5, 93.3, 70.3, 66.0, 62.9, 59.1, 56.7, 55.5, 52.9, 45.6; HRMS (ESI): *m/z* $[M+H]^+$ calcd for $C_{25}H_{29}N_4O_5$ 465.2138, found 465.2139.

1-(3-methoxy-4-(methoxymethyl)-5-((4-methoxyphenyl)(methyl)amino)cyclopent-2-enyl)-5-methylpyrimidine-2,4(1H,3H)-dione (4l):

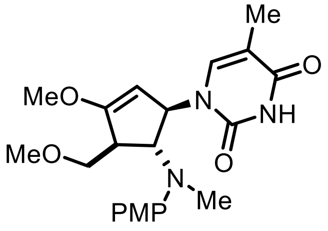

4l

Reaction was carried out according to general procedure described above. Flash column chromatography (hexane/EtOAc 1:1) gave **4l** as colourless oil (36.5 mg, 91%): ^{1}H NMR (CD_3CN, 400 MHz): δ 8.90 (s, 1H), 7.44 (s, 1H), 6.77-6.79 (m, 2H), 6.72-6.75 (m, 2H), 5.71 (d, *J*= 5.2 Hz, 1H), 4.51 (s, 1H), 4.25 (t, *J*= 6.4 Hz, 1H), 3.72 (s, 6H), 3.69 (dd, *J*= 9.6, 3.2 Hz, 1H), 3.32-3.35 (m, 4H), 2.90-2.92 (m, 1H), 2.81 (s, 3H), 1.85 (s, 3H); ^{13}C NMR (CD_3CN, 100 MHz): δ 163.7, 162.1, 152.4, 150.9, 144.6, 137.2, 116.2, 114.3, 109.9, 93.8, 69.5, 68.5, 58.4, 58.3, 56.6, 55.1, 45.8, 33.3, 11.6; HRMS (ESI): *m/z* $[M+H]^+$ calcd for $C_{21}H_{28}N_3O_5$ 402.2029, found 402.2029.

1-(5-(allyl(4-methoxyphenyl)amino)-3-methoxy-4-(methoxymethyl)cyclopent-2-enyl)-5-methylpyrimidine-2,4(1H,3H)-dione (4m):

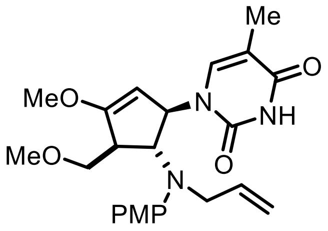

4m

Reaction was carried out according to general procedure described above. Flash column chromatography (hexane/EtOAc 1:1) gave **4m** as white solid (32.1 mg, 75%): ^{1}H NMR (CD_3CN, 400 MHz): δ 9.32 (s, 1H), 7.48 (d, *J*= 1.2 Hz, 1H), 6.76-6.79 (m, 2H), 6.71-6.74 (m, 2H), 5.82-5.89 (m, 1H), 5.76-5.78 (m, 1H), 5.18 (dd, *J*= 17.2, 2.0 Hz, 1H), 5.08 (dd, *J*= 10.4, 1.6 Hz, 1H), 4.53 (s, 1H), 4.13 (dd, *J*= 6.4, 5.2 Hz, 1H), 3.85-3.87 (m, 2H), 3.72 (s, 3H), 3.68-3.71 (m, 4H), 3.41 (dd, *J*= 9.6, 2.8 Hz, 1H), 3.37 (s, 3H), 2.90-2.92 (m, 1H), 1.86 (d, *J*= 0.8 Hz, 3H); ^{13}C NMR (CD_3CN, 100 MHz): δ 163.9, 162.3, 152.6, 151.2, 142.2, 137.5, 136.1, 115.5, 114.3, 114.1, 110.0, 94.2, 69.8, 68.0, 59.6, 58.4, 56.6, 55.1, 51.9, 46.9, 11.7; HRMS (ESI): *m/z* $[M+H]^+$ calcd for $C_{23}H_{30}N_3O_5$ 428.2185, found 428.2185.

1-(3-methoxy-4-(methoxymethyl)-5-((4-methoxyphenyl)(prop-2-ynyl)amino)cyclopent-2-enyl)-5-methylpyrimidine-2,4(1H,3H)-dione (4n):

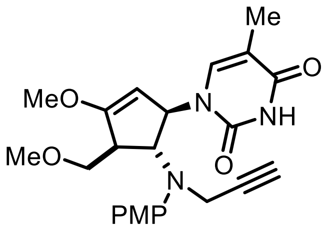

4n

Reaction was carried out according to general procedure described above. Flash column chromatography (hexane/EtOAc 1:1) gave **4n** as white solid (37.9 mg, 89%): ^{1}H NMR (CD_3CN, 400 MHz): δ 9.25 (s, 1H), 7.50 (d, *J*= 1.2 Hz, 1H), 6.90 (d, *J*= 9.2 Hz, 1H), 6.83 (d, *J*= 9.2 Hz, 1H), 5.72-5.74 (m, 1H), 4.52 (s, 1H), 4.16 (t, *J*= 6.0 Hz, 2H), 4.03 (t, *J*= 2.0 Hz, 1H), 3.74 (s, 3H), 4.67-3.71 (m,

4H), 3.36-3.40 (m, 4H), 2.95-2.96 (m, 1H), 1.86 (d, *J*= 0.8 Hz, 3H); ^{13}C NMR (CD_3CN, 100 MHz): δ 163.8, 162.1 ,154.1, 151.1, 141.5, 137.5, 119.5, 114.3, 109.9, 93.9, 80.9, 72.6, 69.7, 67.8, 59.6, 58.4, 56.7, 55.0, 47.1, 39.7, 11.7; HRMS (ESI): *m/z* $[M+H]^+$ calcd for $C_{23}H_{28}N_3O_5$ 426.2029, found 426.2029.

1-(3-methoxy-5-((4-methoxybenzyl)(4-methoxyphenyl)amino)-4-(methoxymethyl)cyclopent-2-enyl)-5-methylpyrimidine-2,4(1H,3H)-dione (4o):

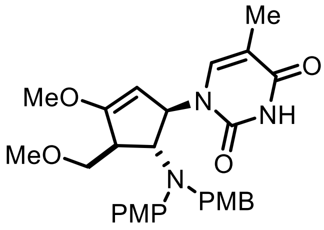

4o

Reaction was carried out according to general procedure described above. Flash column chromatography (hexane/EtOAc 1:1) gave **4o** as colourless solid (46.7 mg, 92%): ^1H NMR (CD_3CN, 400 MHz): δ 9.37 (s, 1H), 7.31 (d, *J*= 1.2 Hz, 1H), 7.22 (d, *J*= 8.8 Hz, 2H), 6.80-6.85 (m, 4H), 6.74-6.77 (m, 2H), 5.84-5.87 (m, 1H), 4.50 (s, 1H), 4.36 (d, *J*= 15.2 Hz, 1H), 4.30 (d, *J*= 15.2 Hz, 1H), 4.09 (dd, *J*= 6.4, 2.4 Hz, 1H), 3.75 (s, 3H), 3.68-3.72 (m, 7H), 3.41 (dd, *J*= 9.6, 2.8 Hz, 1H), 3.39 (s, 3H), 2.93-2.95 (m, 1H), 1.79 (d, *J*= 1.2 Hz, 3H); ^{13}C NMR (CD_3CN, 100 MHz): δ 163.9, 162.2, 158.7, 153.2, 151.3, 142.2, 137.2, 131.3, 129.0, 119.0, 114.3, 113.6, 109.9, 94.1, 70.0, 68.0, 59.7, 58.3, 56.6, 55.0, 54.8, 52.6, 46.3, 11.6; HRMS (ESI): *m/z* $[M+H]^+$ calcd for $C_{28}H_{34}N_3O_6$ 508.2448, found 508.2449.

1-(5-(benzyl(4-chlorophenyl)amino)-3-methoxy-4-(methoxymethyl)cyclopent-2-enyl)-5-methylpyrimidine-2,4(1H,3H)-dione (4p):

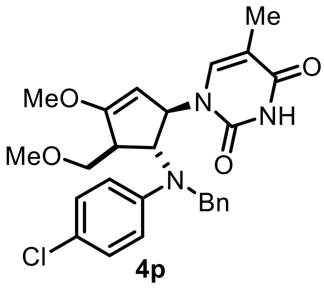

4p

Reaction was carried out according to general procedure described above. Flash column chromatography (hexane/EtOAc 1:1) gave **4p** as yellow oil (27.0 mg, 56%): ^{1}H NMR (CD_3CN, 400 MHz): δ 9.06 (s, 1H), 7.38 (s, 1H), 7.28-7.32 (m, 4H), 7.22-7.27 (m, 1H), 7.09-7.13 (m, 2H), 6.67-6.71 (m, 2H), 5.83-5.85 (m, 1H), 4.56 (s, 1H), 4.55 (d, *J*= 16.8 Hz, 2H), 4.49 (d, *J*= 16.8, 1H), 4.29 (dd, *J*= 6.0, 5.2 Hz, 1H), 3.73 (s, 3H), 3.70 (dd, *J*= 9.6, 3.2 Hz, 1H), 3.44 (dd, *J*= 9.6, 3.2 Hz, 1H), 3.35 (s, 3H), 2.98-3.00 (m, 1H), 1.82 (d, *J*= 0.8 Hz, 3H); ^{13}C NMR (CD_3CN, 100 MHz): δ 163.7, 162.2, 151.1, 147.0, 138.9, 137.1, 128.6, 128.5, 127.1, 126.9, 121.9, 116.8, 116.1, 110.0, 94.1, 70.0, 67.4, 59.9, 58.4, 56.8, 52.5, 46.7, 11.6; HRMS (ESI): *m/z* $[M+H]^+$ calcd for $C_{26}H_{29}ClN_3O_4$ 482.1847, found 482.1848.

1-(5-(benzyl(4-methoxyphenyl)amino)-3-(benzyloxy)-4-(benzyloxymethyl)cyclopent-2-enyl)-5-methylpyrimidine-2,4(1H,3H)-dione (4q):

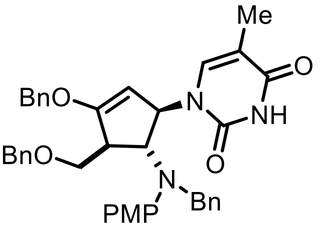

4q

Reaction was carried out according to general procedure described above. Flash column chromatography (hexane/EtOAc 2:1) gave **4q** as brown oil (57.3 mg, 91%): ^{1}H NMR (CD_3CN, 400 MHz): δ 9.84 (s, 1H), 7.32 (d, *J*= 6.0 Hz, 2H), 7.21-7.24 (m, 10H), 7.14 (t, *J*= 7.6 Hz, 2H), 7.05-7.09 (m, 2H), 6.77 (d, *J*= 7.2 Hz, 2H), 6.62 (d, *J*= 7.2 Hz, 2H), 5.88 (d, *J*= 4.8 Hz, 1H), 4.97 (d, *J*= 12.8 Hz, 1H), 4.93 (d, *J*= 12.8 Hz, 1H), 4.46-4.49 (m, 2H), 4.40 (d, *J*= 12.0 Hz, 2H), 4.16 (s, 2H), 3.87 (dd, *J*= 9.6, 5.6 Hz, 1H), 3.57 (s, 3H), 3.53 (dd, *J*= 9.6, 2.4 Hz, 1H), 2.98-3.00 (m, 1H), 1.34 (s, 3H); ^{13}C NMR (CD_3CN, 100 MHz): δ 163.4, 160.4, 153.5, 151.0, 142.1, 139.5, 138.4, 136.7, 136.6, 128.3, 128.1, 127.7, 127.6, 127.5, 127.4, 127.1, 126.6, 119.6, 114.1, 109.7, 95.9, 73.0, 70.8, 67.8, 67.5, 59.5, 51.6, 53.9, 46.9, 11.4; HRMS (ESI): *m/z* $[M+H]^+$ calcd for $C_{39}H_{40}N_3O_5$ 630.2968, found 630.2968.

1-(5-(benzyl(4-methoxyphenyl)amino)-3-(methoxymethoxy)-4-((methoxymethoxy)methyl)cyclopent-2-enyl)-5-methylpyrimidine-2,4(1H,3H)-dione (4r):

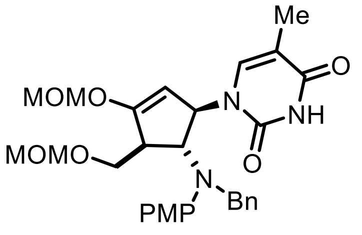

4r

Reaction was carried out according to general procedure described above. Flash column chromatography (hexane/EtOAc 2:1) gave **4r** as brown oil (27.9 mg, 52%): ^{1}H NMR ($CDCl_3$, 400 MHz): δ 8.41 (s, 1H), 7.36 (s, 1H), 6.84 (d, *J*= 9.2 Hz, 1H), 5.97 (s, 1H), 5.04 (s, 1H), 4.63 (d, *J*= 5.6 Hz, 1H), 4.57 (d, *J*= 6.4 Hz, 1H), 4.43 (d, *J*= 16.0 Hz, 1H), 4.39 (d, *J*= 16.0 Hz, 1H), 4.08 (t, *J*= 5.6, 1H), 3.88 (dd, *J*= 8.0 Hz, 1H), 3.74 (s, 3H), 3.59 (d, *J*= 8.0 Hz, 1H), 3.46 (s, 3H), 3.27 (s, 3H), 2.99 (m, 1H), 1.87 (s, 3H); ^{13}C NMR ($CDCl_3$, 100 MHz): δ 163.7, 158.5, 153.7, 150.7, 142.0, 139.1, 137.3, 128.5, 127.4, 127.0, 119.6, 114.5, 110.5, 97.4, 96.7, 95.0, 67.2, 65.1, 60.3, 56.4, 55.5, 55.3, 54.7, 46.9, 12.6; HRMS (ESI): *m/z* $[M+H]^+$ calcd for $C_{29}H_{36}N_3O_7$ 538.2553, found 538.2551.

1-(5-(benzyl(4-methoxyphenyl)amino)-3-methoxy-4-methylcyclopent-2-enyl)-5-methylpyrimidine-2,4(1H,3H)-dione (4s):

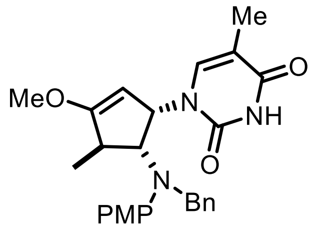

4s

Reaction was carried out according to general procedure described above. Flash column chromatography (hexane/EtOAc 1:1) gave **4s** as brown oil (38.9 mg, 87%): ^{1}H NMR (CD_3CN, 400 MHz): δ 9.29 (s, 1H), 7.35 (d, *J*= 7.6 Hz, 2H), 7.24-7.28 (m, 3H), 7.18 (t, *J*= 7.2 Hz, 1H), 6.81-6.84 (m, 2H), 6.67-6.70 (m, 2H), 5.82-5.84 (m, 1H), 4.46 (s, 2H), 4.39 (s, 1H), 3.77 (t, *J*= 7.6 Hz, 1H), 3.69 (s, 3H), 3.66 (s, 3H), 2.88-2.95 (m, 1H), 1.82 (d, *J*= 1.2 Hz, 3H), 1.28 (d, *J*= 6.8 Hz, 1H); ^{13}C NMR (CD_3CN, 100 MHz): δ 164.4, 163.8, 153.3, 151.3, 142.6, 139.7, 136.7, 136.6, 128.2, 127.6, 126.7,

119.6, 114.0, 110.2, 91.4, 91.3, 76.3, 59.2, 56.4, 56.3, 55.0, 54.9, 51.6, 39.7, 15.8, 11.4; HRMS (ESI): *m/z* $[M+H]^+$ calcd for $C_{26}H_{30}N_3O_4$ 448.2236, found 448.2238.

Characterization Data of 7

4-methoxy-5-(methoxymethyl)-N1-(4-methoxyphenyl)cyclopentane-1,2-diamine (7):

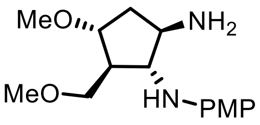

7

Reaction was carried out according to procedure described above. Flash column chromatography (CH_2Cl_2/MeOH 9:1) gave **7** as brown oil (27 mg, 84% over 2 step): ^1H NMR (CD_3OD, 400 MHz): δ 6.73-6.74 (m, 4H), 3.71-3.72 (m, 4H), 3.48 (dd, *J*= 9.2, 3.6 Hz, 1H), 3.32-3.37 (m, 5H), 3.31 (s, 3H), 3.28 (s, 3H), 3.19 (dd, *J*= 17.6, 8.0 Hz, 1H); ^{13}C NMR ($CDCl_3$, 100 MHz): δ 152.3, 142.8, 115.0, 114.4, 80.4, 71.7, 63.2, 57.7, 56.1, 55.4, 54.8, 52.9, 36.5; HRMS (ESI): *m/z* $[M+H]^+$ calcd for $C_{14}H_{22}N_2O_3$ 266.1630, found 266.1630.

1H, 13C and 2D (COSY and NOESY) NMR Spectra

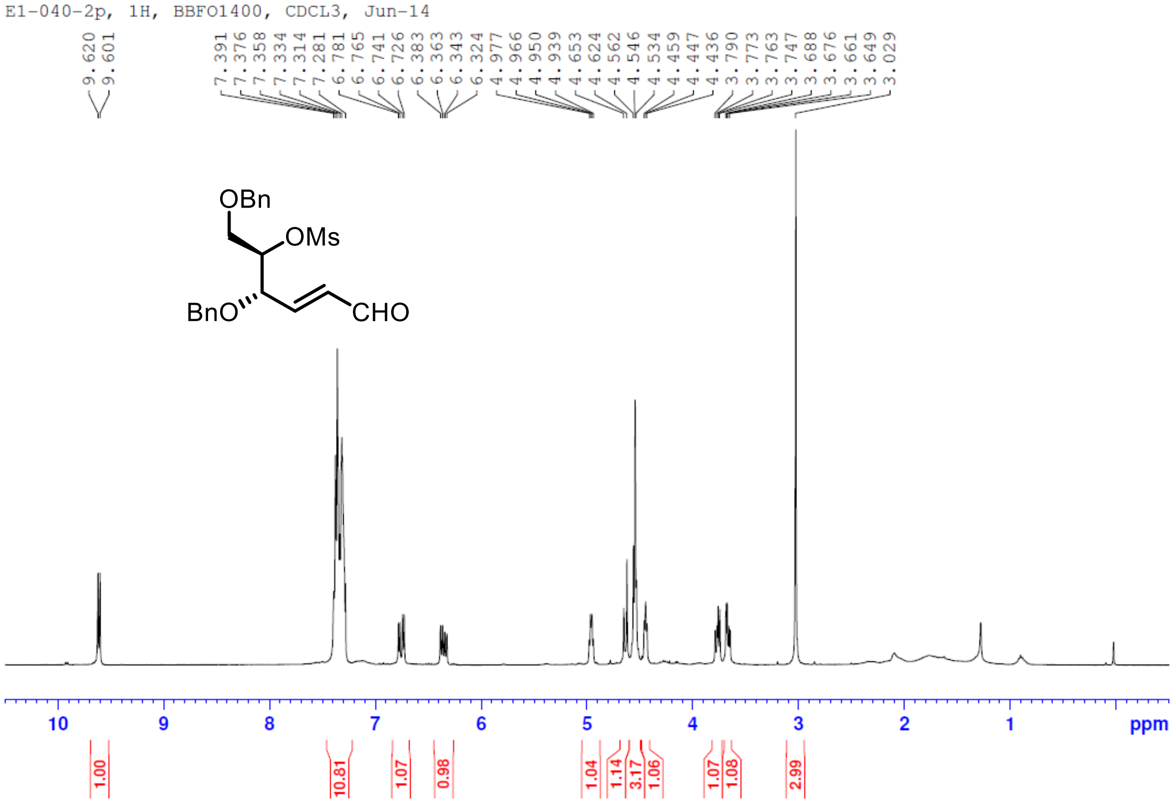

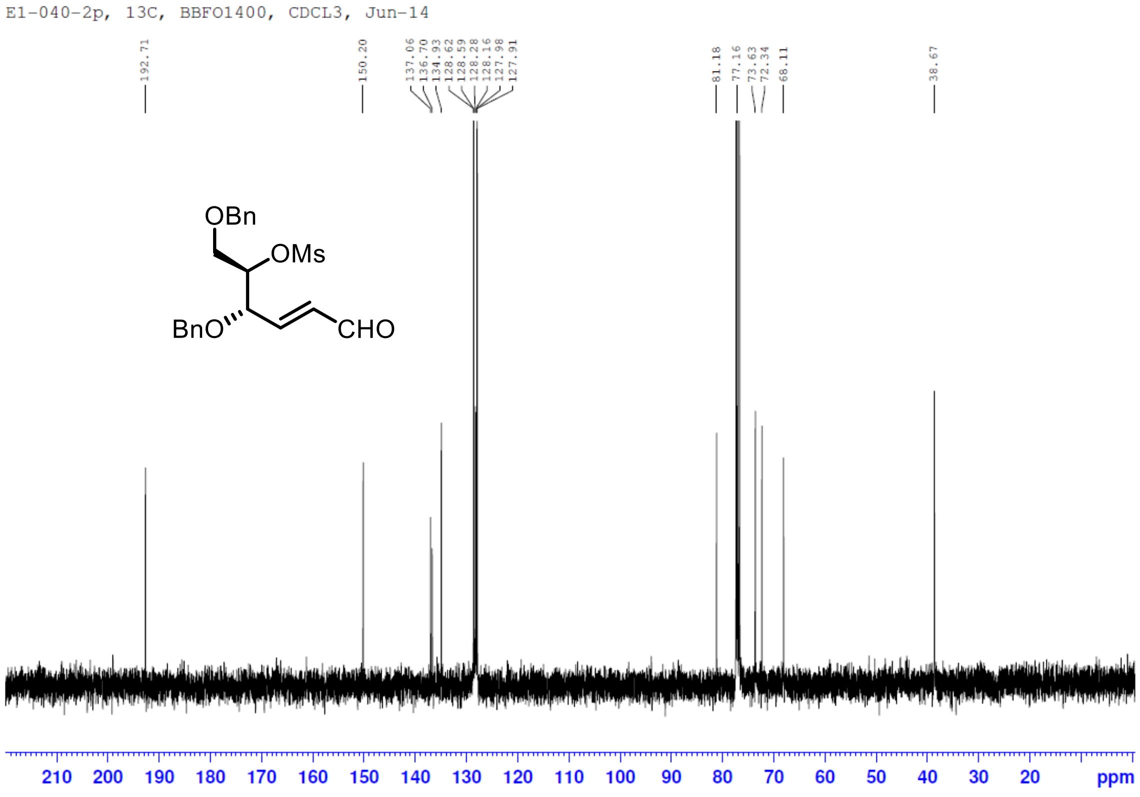

E1-043-2p, 1H, BBFO2 400, CDCl3, Jun-14

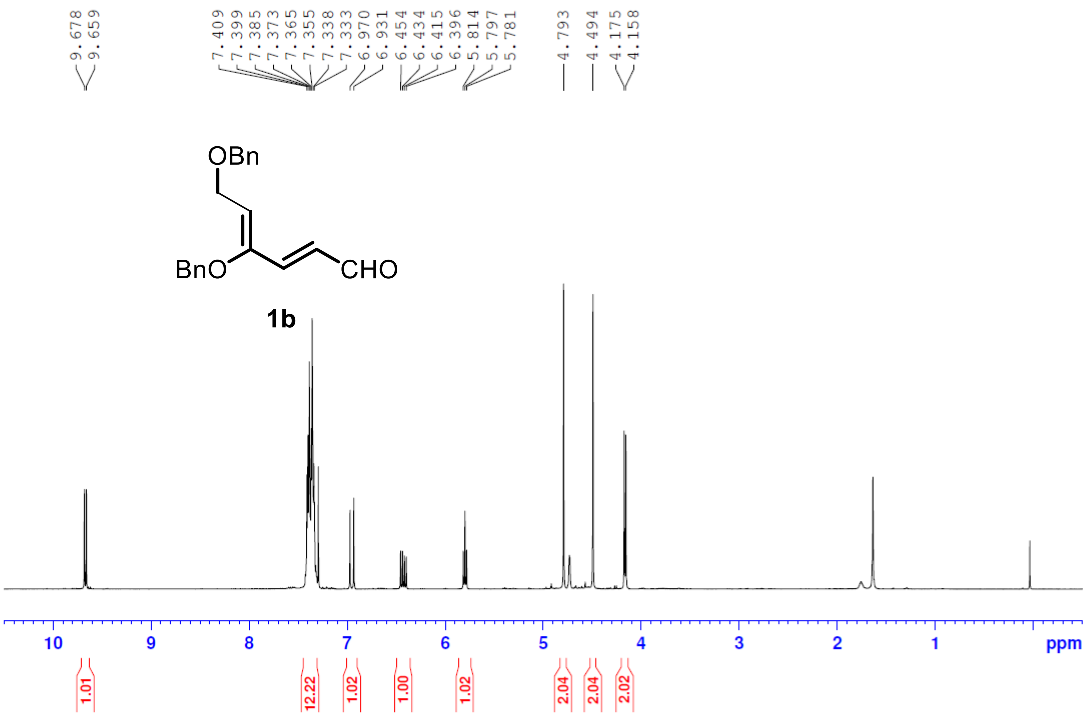

E1-043-2p, 13C, BBFO2 400, CDCl3, Jun-14

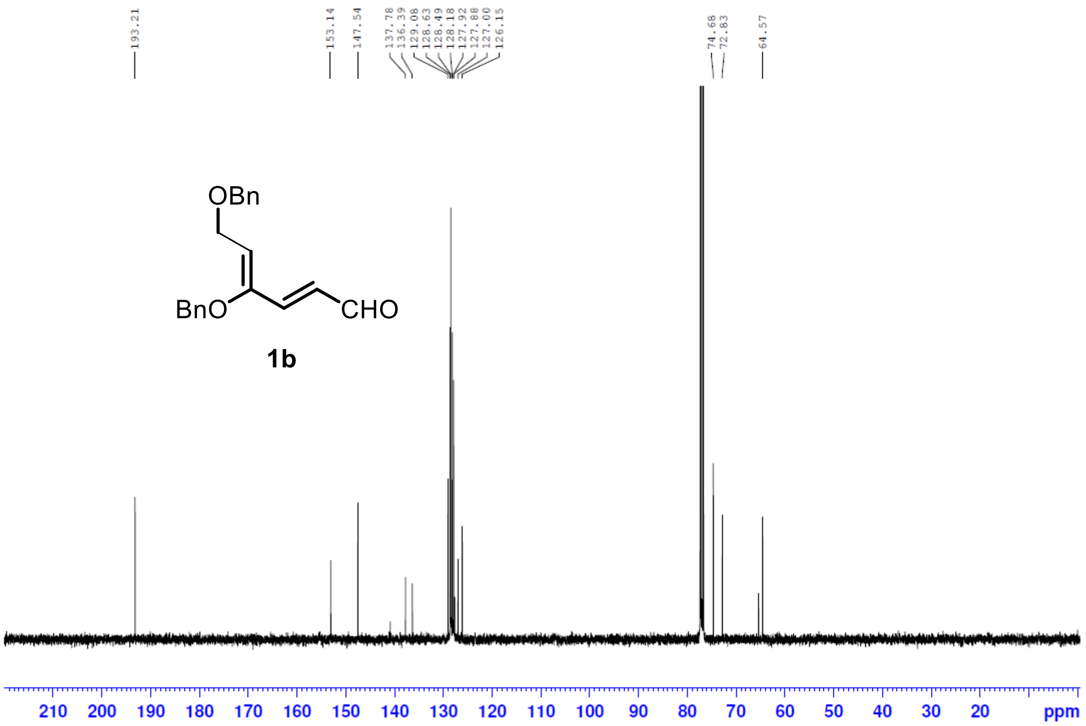

E1-043-1p, 1H, BBFO2 400, CDCl3, Jun-14

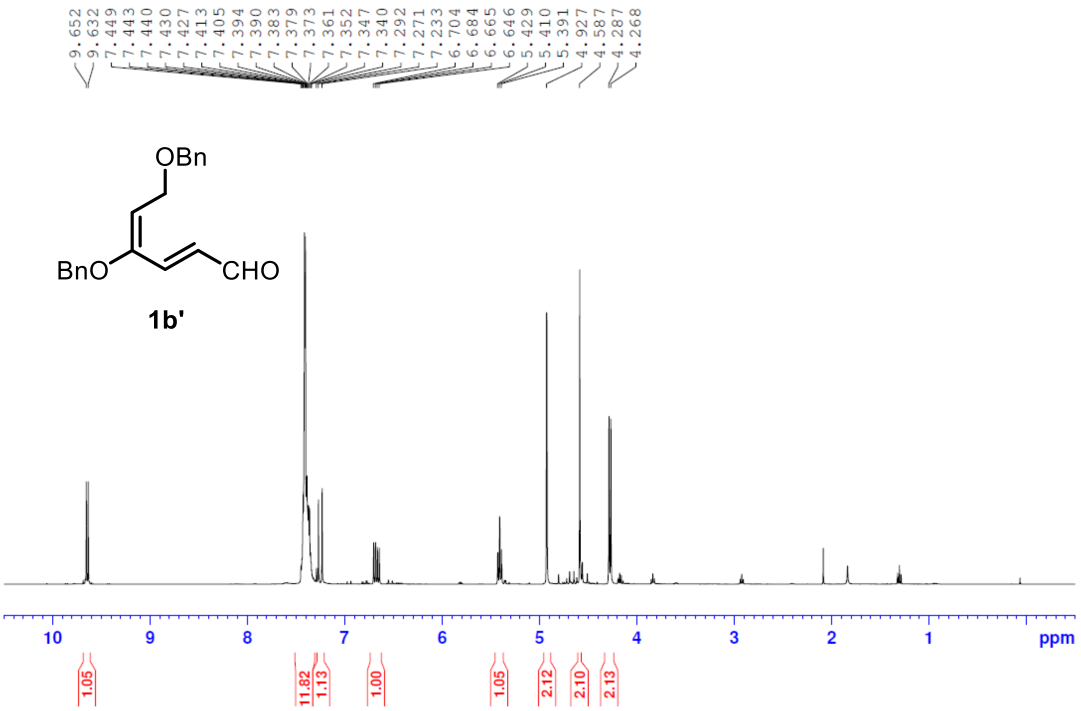

E1-043-1p, 13C, BBFO2 400, CDCl3, Jun-14

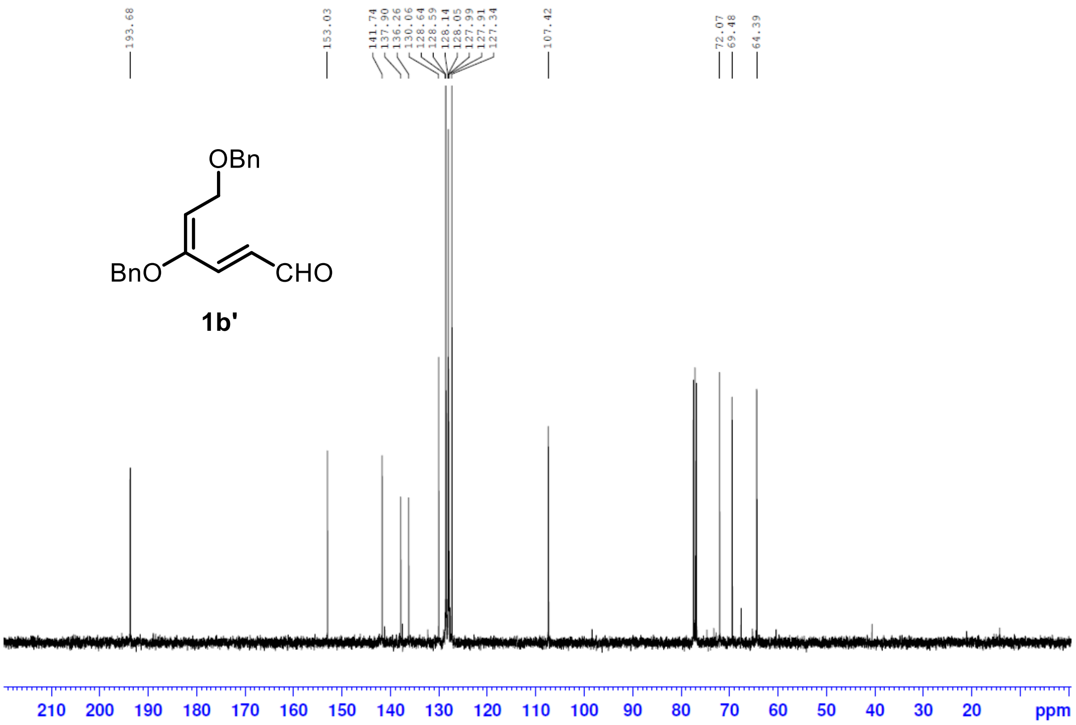

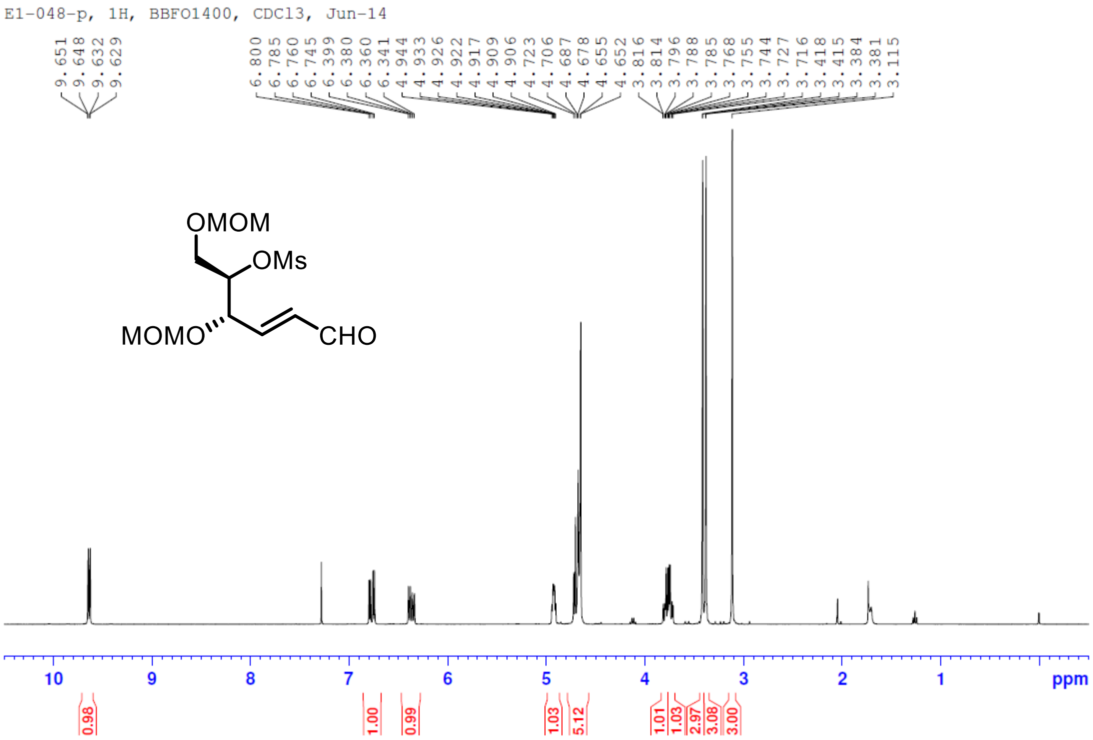
E1-048-p, 1H, BBFO1400, CDCl3, Jun-14
9.651 9.648 9.632 9.629
6.800 6.785 6.760 6.745 6.399 6.380 6.360 6.341 4.944 4.933 4.926 4.922 4.917 4.909 4.906 4.723 4.706 4.687 4.678 4.655 4.652 3.816 3.814 3.796 3.788 3.785 3.768 3.755 3.744 3.727 3.716 3.418 3.415 3.384 3.381 3.115
OMOM
OMs
MOMO
CHO
10 9 8 7 6 5 4 3 2 1 ppm
0.98 1.00 0.99 1.03 5.12 1.01 1.03 2.97 3.08 3.00

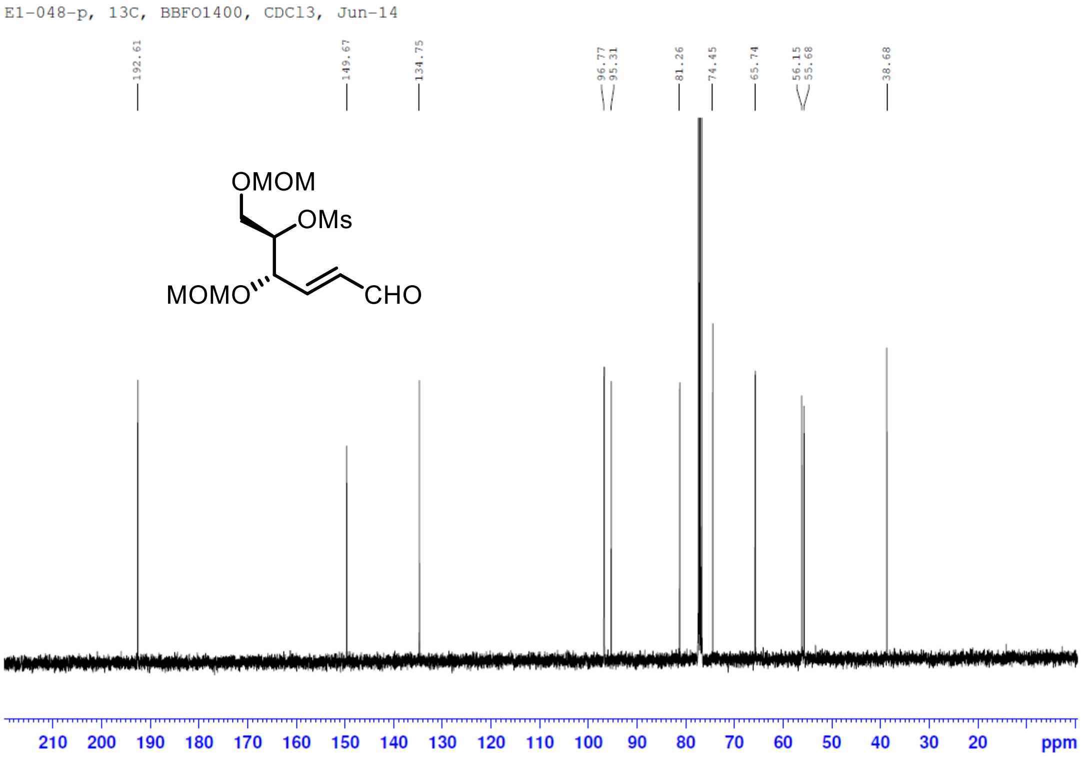
E1-048-p, 13C, BBFO1400, CDCl3, Jun-14
192.61 149.67 134.75 96.77 95.31 81.26 74.45 65.74 56.15 55.68 38.68
OMOM
OMs
MOMO
CHO
210 200 190 180 170 160 150 140 130 120 110 100 90 80 70 60 50 40 30 20 ppm

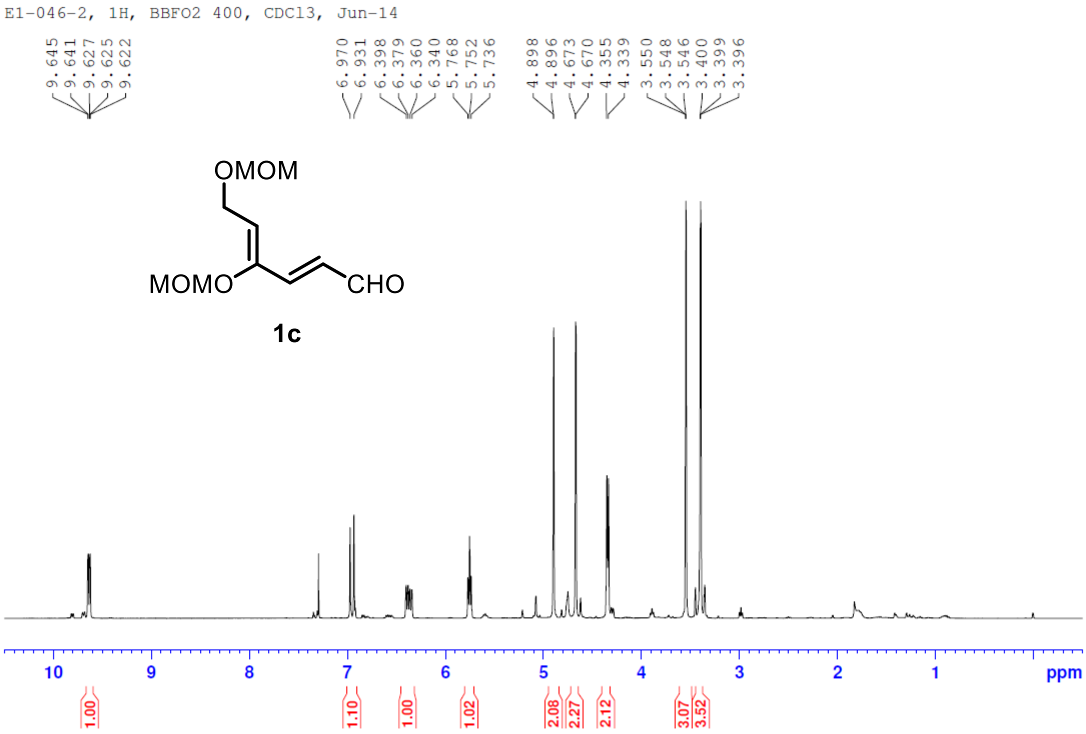
E1-046-2, 1H, BBFO2 400, CDCl3, Jun-14
9.645 9.641 9.627 9.625 9.622
6.970 6.931 6.398 6.379 6.360 6.340 5.768 5.752 5.736
4.898 4.896 4.673 4.670 4.355 4.339 3.550 3.548 3.546 3.400 3.399 3.396
OMOM
MOMO
CHO
1c
10 9 8 7 6 5 4 3 2 1 ppm
1.00 1.10 1.00 1.02 2.08 2.27 2.12 3.07 3.52

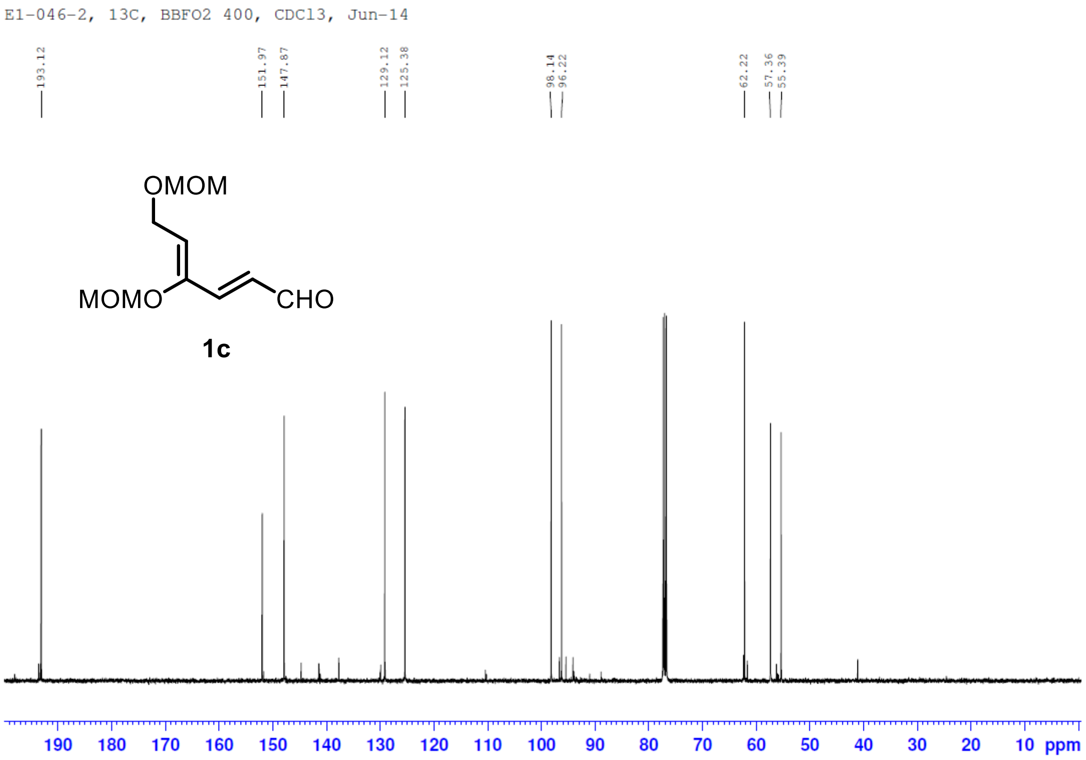
E1-046-2, 13C, BBFO2 400, CDCl3, Jun-14
193.12 151.97 147.87 129.12 125.38 98.14 96.22 62.22 57.36 55.39
OMOM
MOMO
CHO
1c
190 180 170 160 150 140 130 120 110 100 90 80 70 60 50 40 30 20 10 ppm

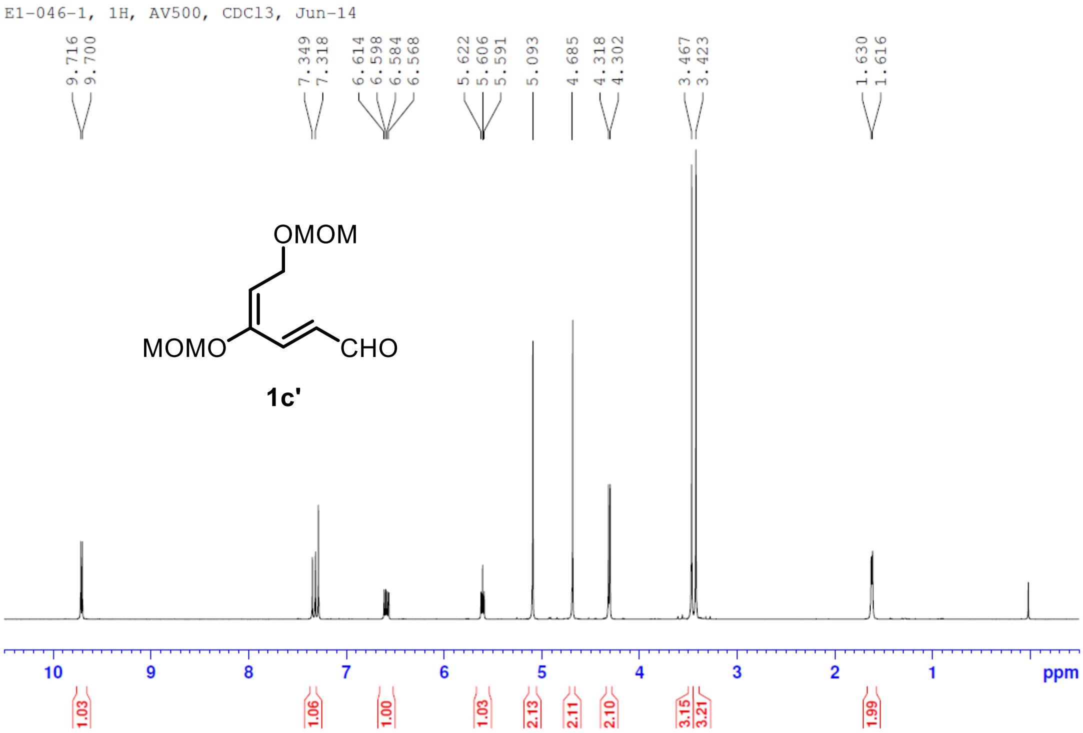
E1-046-1, 1H, AV500, CDCl3, Jun-14
9.716
9.700
7.349
7.318
6.614
6.598
6.584
6.568
5.622
5.606
5.591
5.093
4.685
4.318
4.302
3.467
3.423
1.630
1.616
OMOM
MOMO
CHO
1c'
10
9
8
7
6
5
4
3
2
1
ppm
1.03
1.06
1.00
1.03
2.13
2.11
2.10
3.15
3.21
1.99

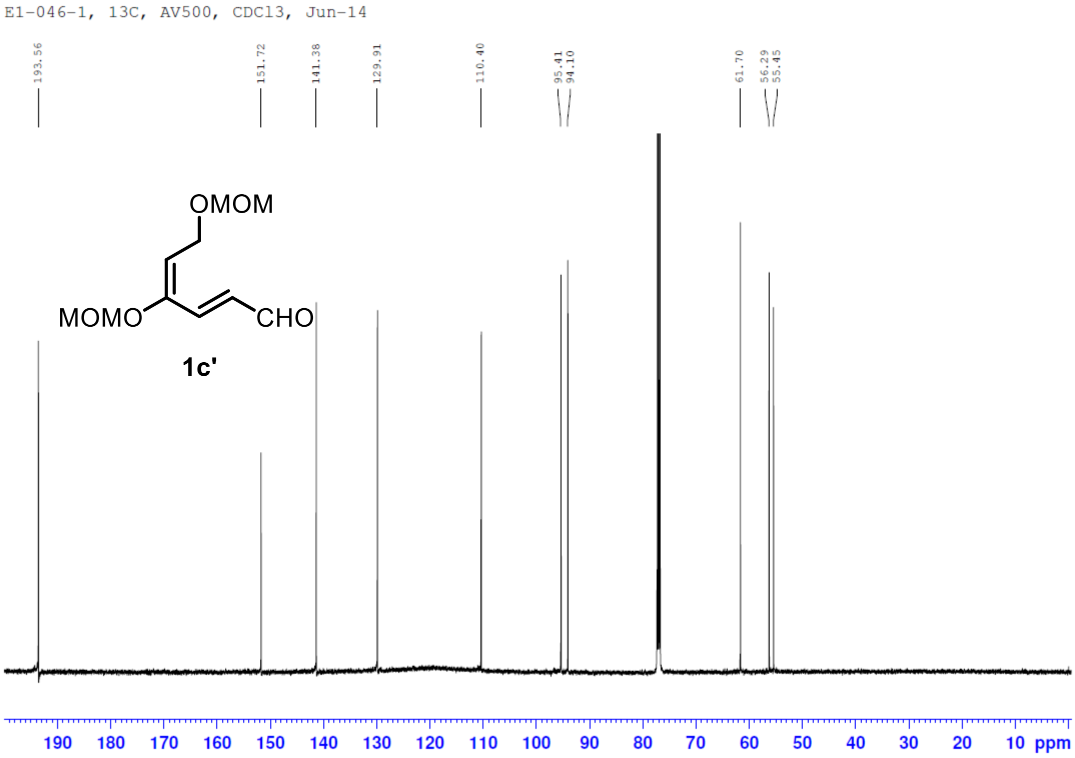
E1-046-1, 13C, AV500, CDCl3, Jun-14
193.56
151.72
141.38
129.91
110.40
95.41
94.10
61.70
56.29
55.45
OMOM
MOMO
CHO
1c'
190
180
170
160
150
140
130
120
110
100
90
80
70
60
50
40
30
20
10
ppm

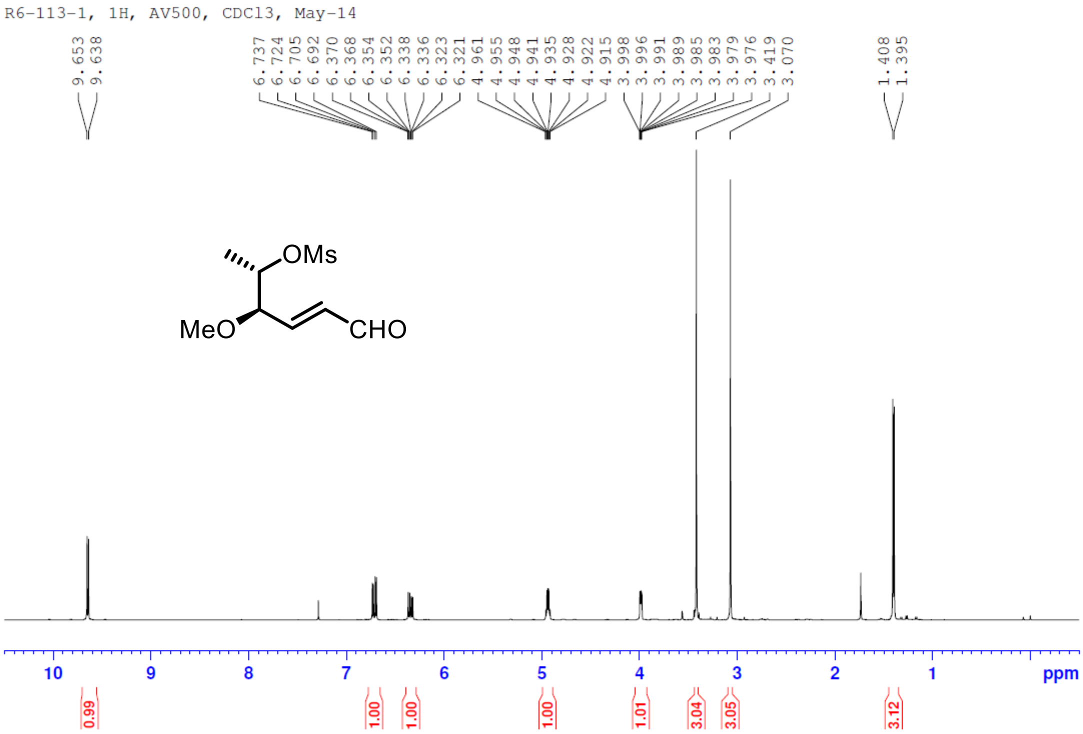
R6-113-1, 1H, AV500, CDCl3, May-14
9.653
9.638
6.737
6.724
6.705
6.692
6.370
6.368
6.354
6.352
6.338
6.336
6.323
6.321
4.961
4.955
4.948
4.941
4.935
4.928
4.922
4.915
3.998
3.996
3.991
3.989
3.985
3.983
3.979
3.976
3.419
3.070
1.408
1.395
OMs
MeO
CHO
ppm
0.99
1.00
1.00
1.00
1.01
3.04
3.05
3.12

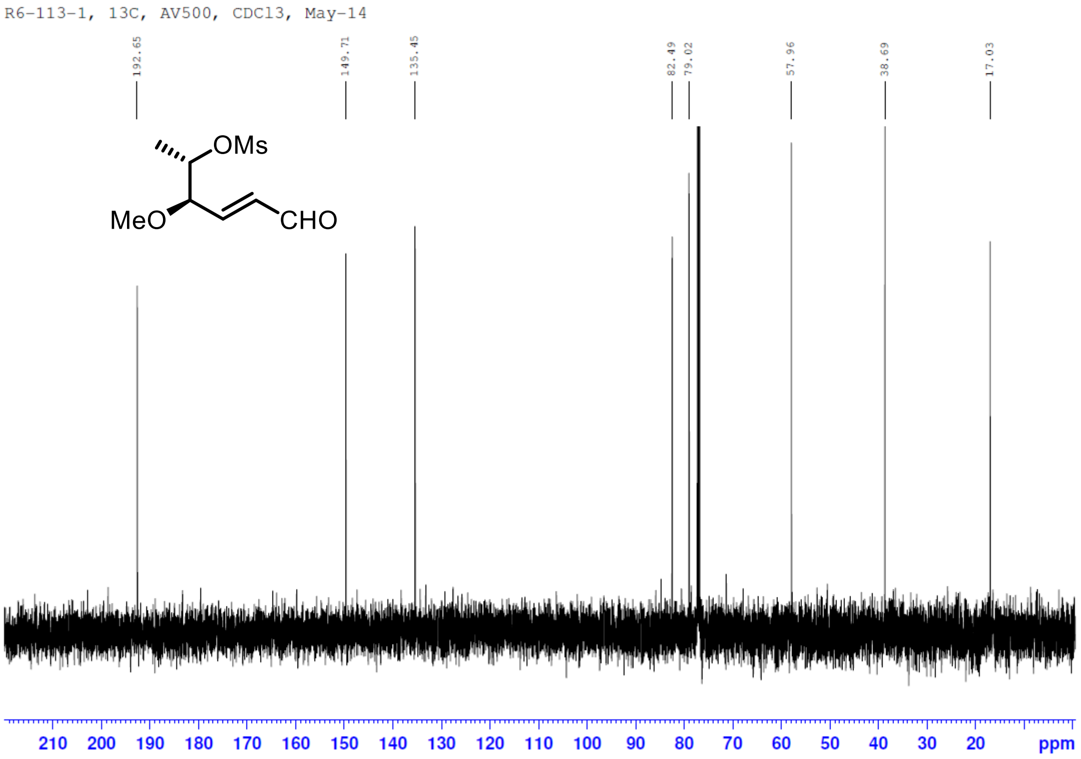
R6-113-1, 13C, AV500, CDCl3, May-14
192.65
149.71
135.45
82.49
79.02
57.96
38.69
17.03
OMs
MeO
CHO
ppm

R8-041-2, 1H, BBFO1400, CDCl3, Jun-15

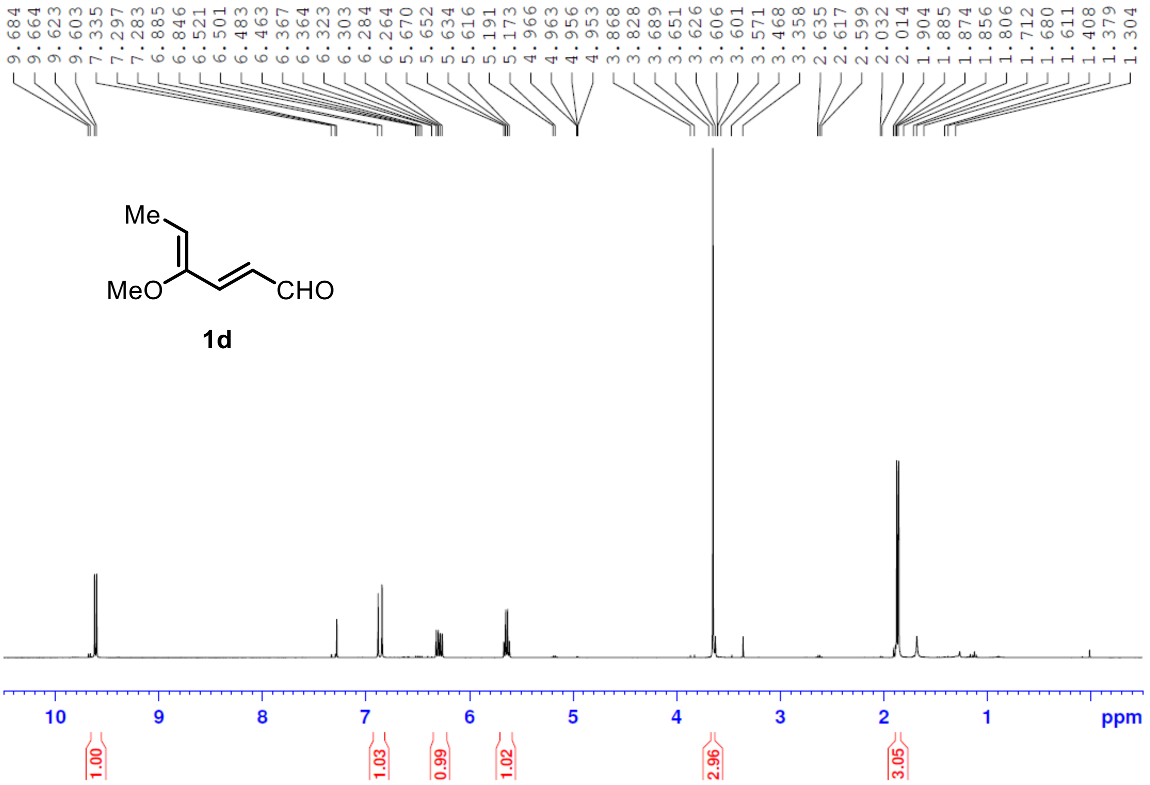

R8-041-2, 13C, BBFO1400, CDCl3, Jun-15

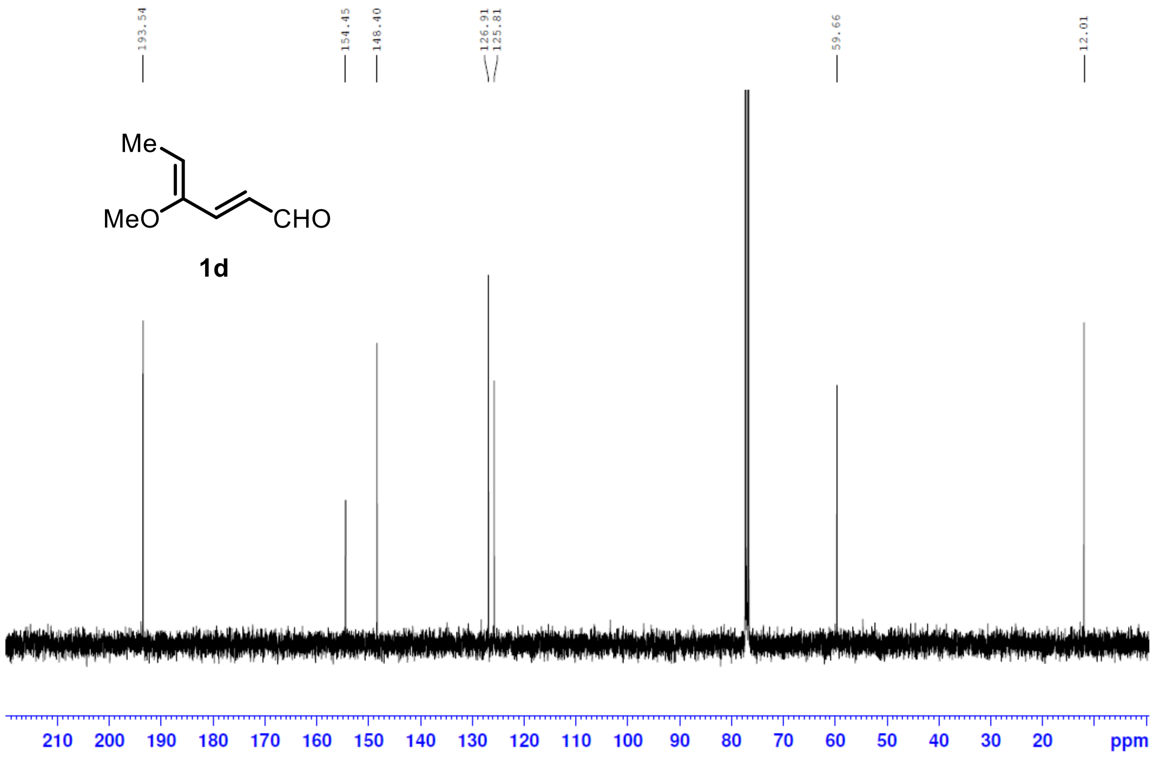

R6-114-1, 1H, BBFO2 400, CDCl3, May-14

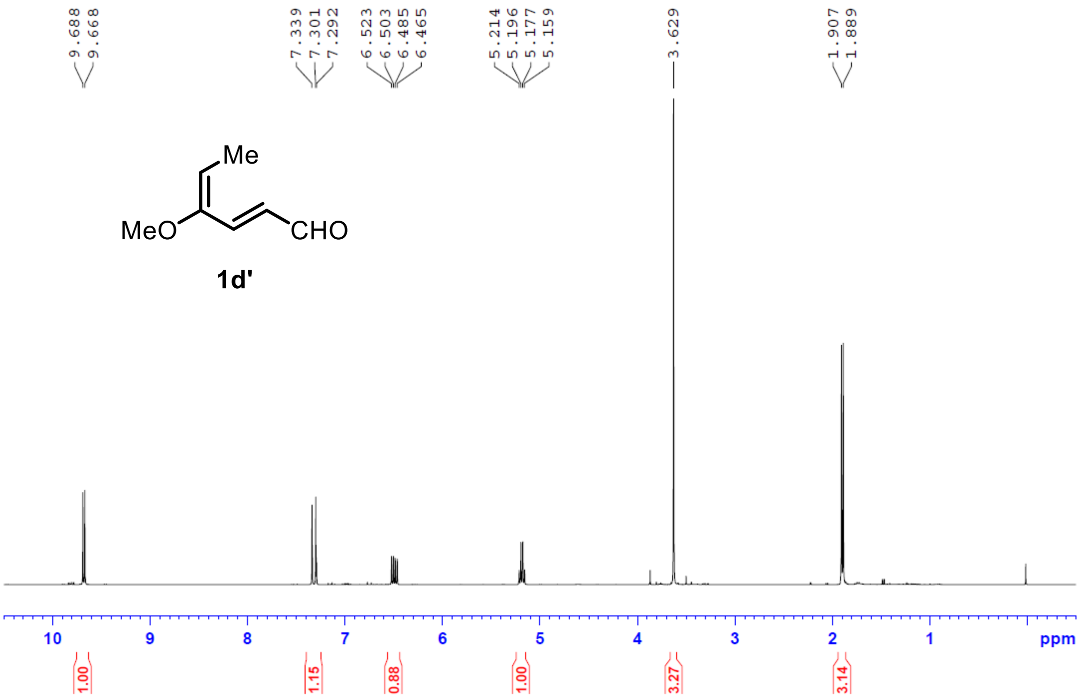

R6-114-1, 13C, BBFO2 400, CDCl3, May-14

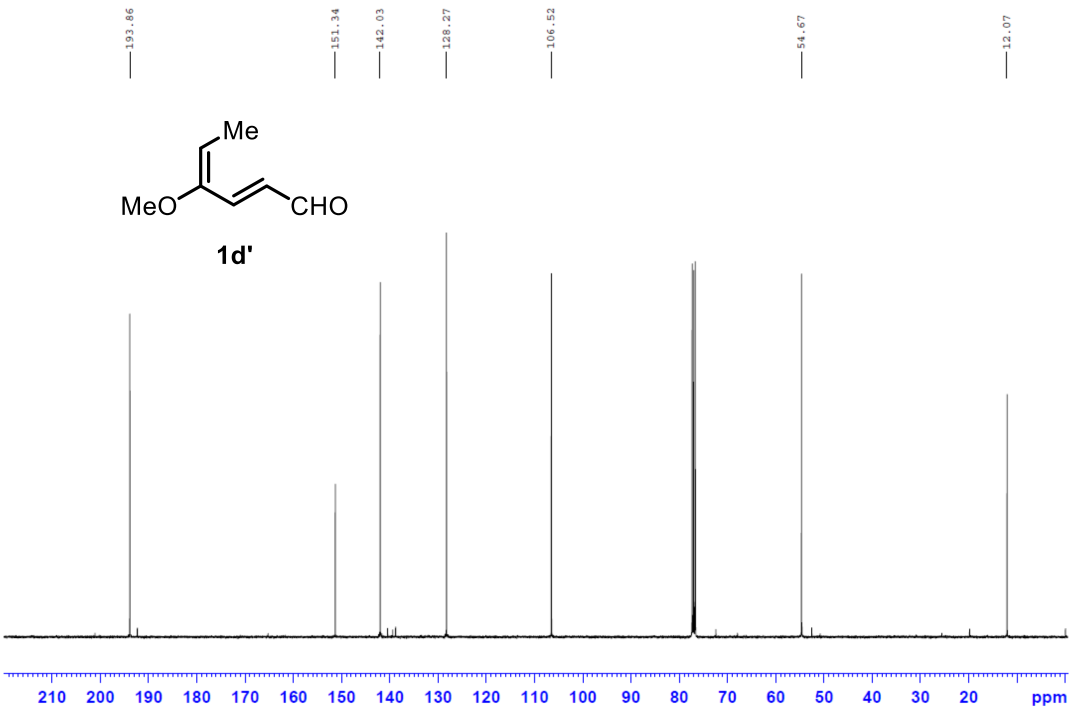

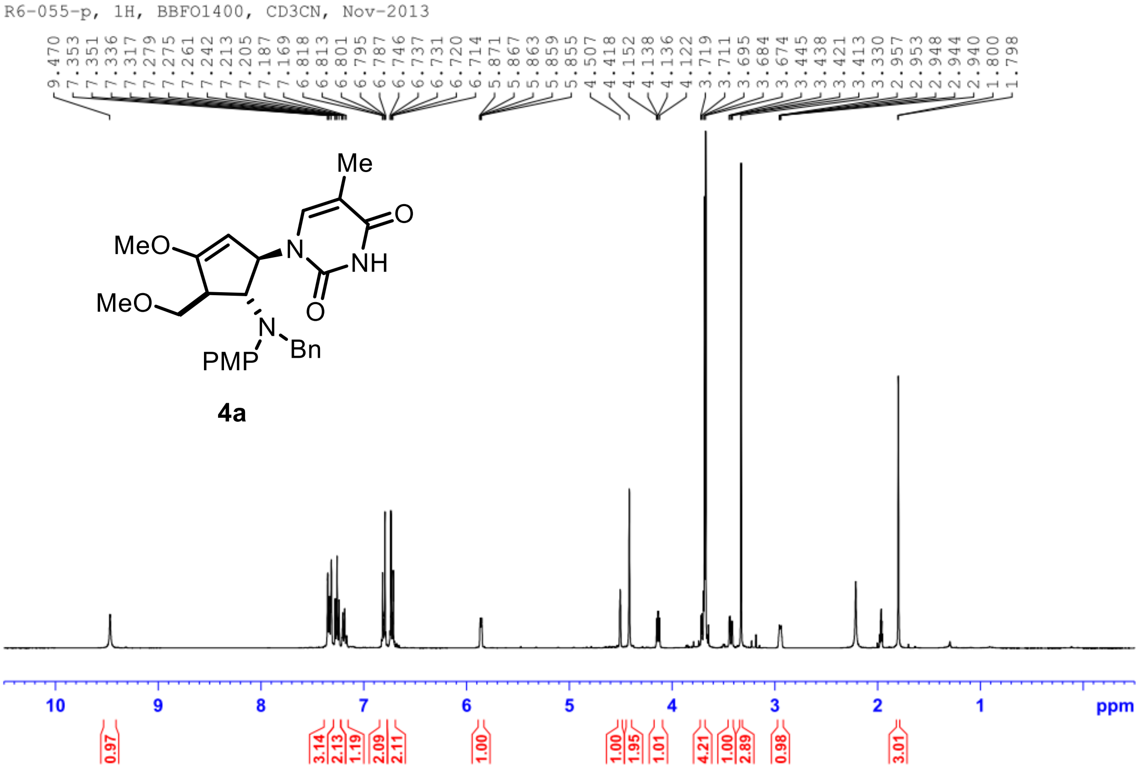
R6-055-p, 1H, BBFO1400, CD3CN, Nov-2013
9.470 7.353 7.351 7.336 7.317 7.279 7.275 7.261 7.242 7.213 7.205 7.187 7.169 6.818 6.813 6.801 6.795 6.787 6.746 6.737 6.731 6.720 6.714 5.871 5.867 5.863 5.859 5.855 4.507 4.418 4.152 4.138 4.136 4.122 3.719 3.711 3.695 3.684 3.674 3.445 3.438 3.421 3.413 3.330 2.957 2.953 2.948 2.944 2.940 1.800 1.798
Me
O
MeO
N
NH
MeO
O
N
Bn
PMP
4a
10 9 8 7 6 5 4 3 2 1 ppm
0.97 3.14 2.13 1.19 2.09 2.11 1.00 1.00 1.95 1.01 4.21 1.00 2.89 0.98 3.01

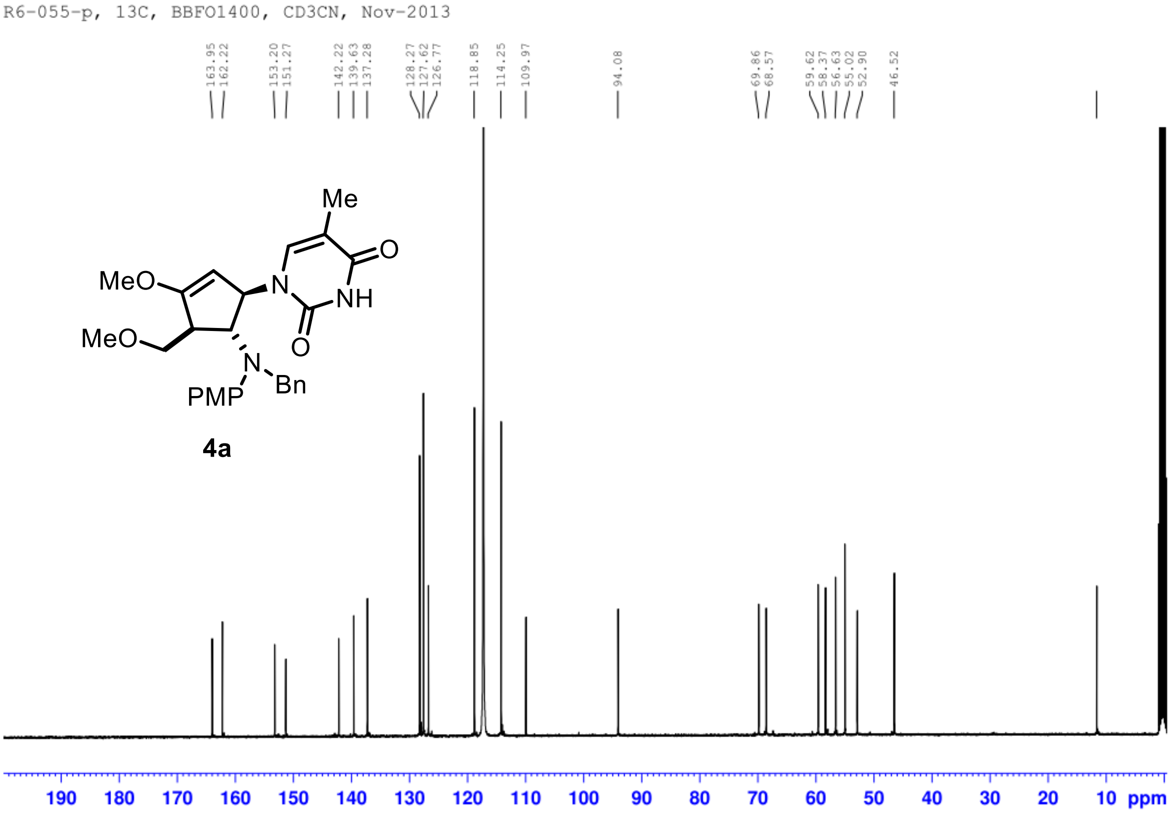
R6-055-p, 13C, BBFO1400, CD3CN, Nov-2013
163.95 162.22 153.20 151.27 142.22 139.63 137.28 128.27 127.62 126.77 118.85 114.25 109.97 94.08 69.86 68.57 59.62 58.37 56.63 55.02 52.90 46.52
Me
O
MeO
N
NH
MeO
O
N
Bn
PMP
4a
190 180 170 160 150 140 130 120 110 100 90 80 70 60 50 40 30 20 10 ppm

COSY of **4a**

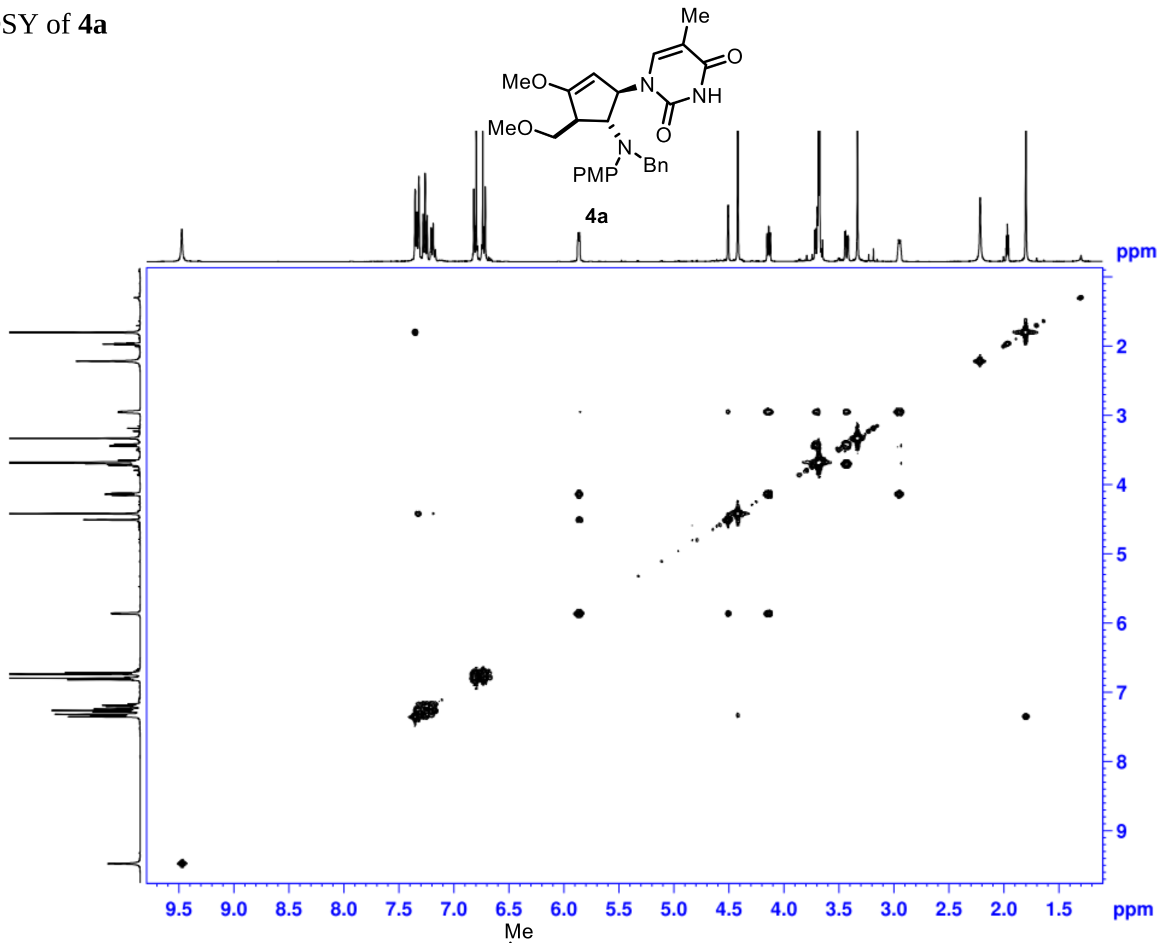
Me
MeO
N
O
NH
MeO
O
N
PMP
Bn
4a
ppm
2
3
4
5
6
7
8
9
9.5
9.0
8.5
8.0
7.5
7.0
6.5
6.0
5.5
5.0
4.5
4.0
3.5
3.0
2.5
2.0
1.5
ppm

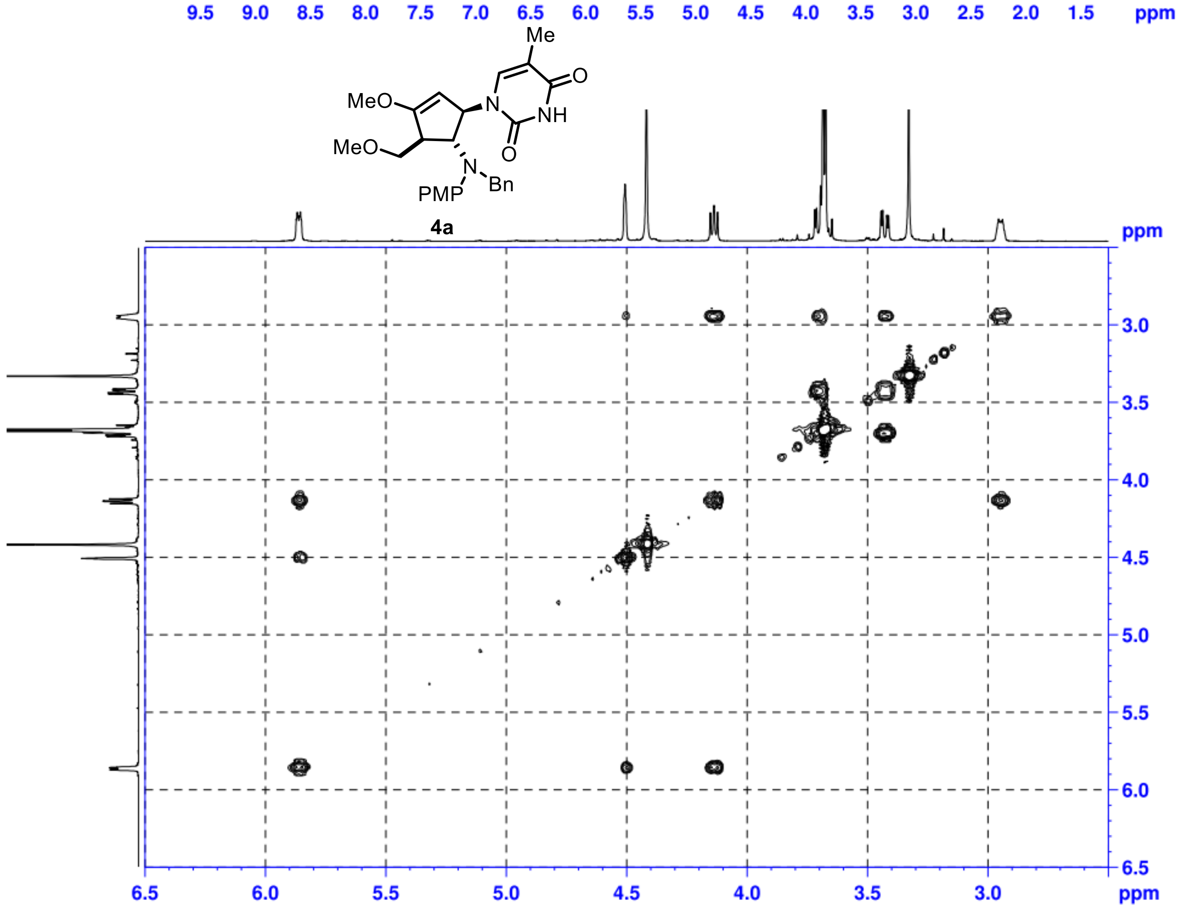
Me
MeO
N
O
NH
MeO
O
N
PMP
Bn
4a
ppm
3.0
3.5
4.0
4.5
5.0
5.5
6.0
6.5
6.5
6.0
5.5
5.0
4.5
4.0
3.5
3.0
ppm

NOESY of **4a**

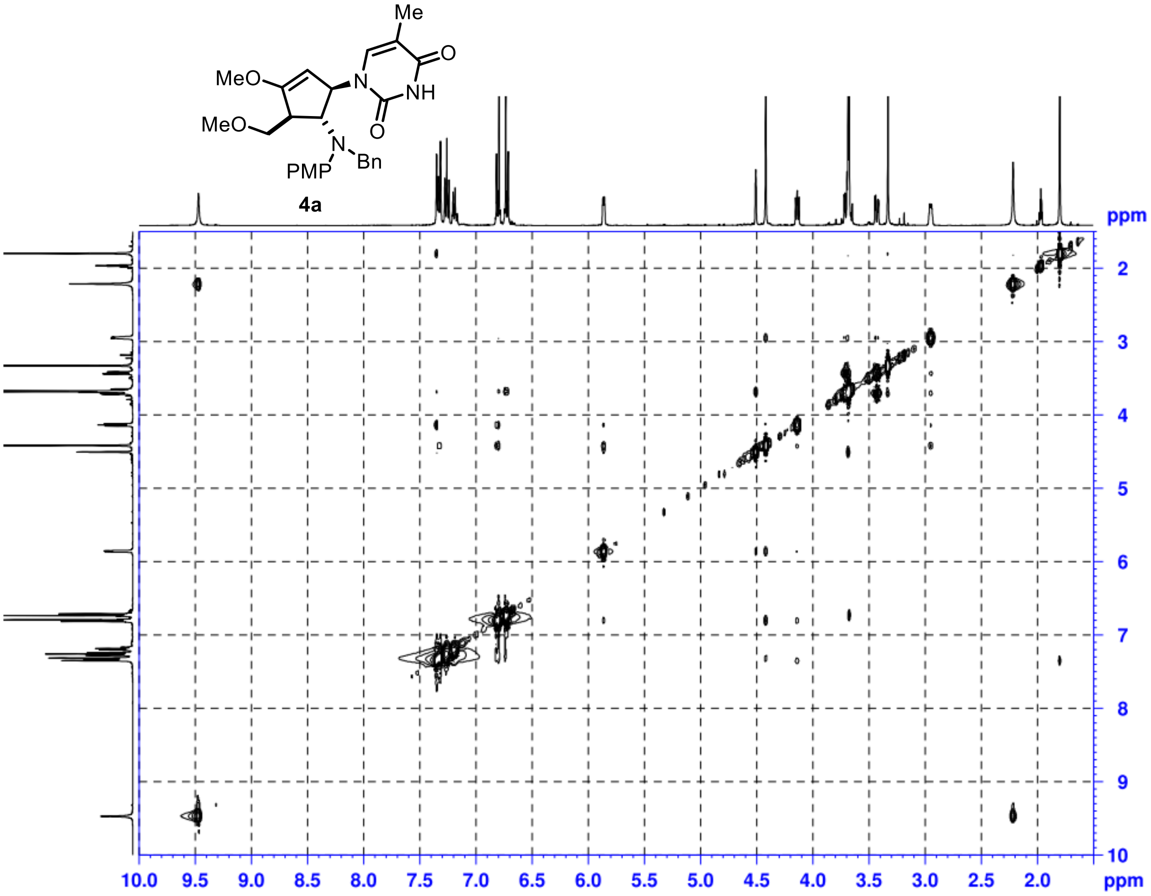

Me
O
MeO
N
NH
MeO
O
N
Bn
PMP
4a
ppm
10.0 9.5 9.0 8.5 8.0 7.5 7.0 6.5 6.0 5.5 5.0 4.5 4.0 3.5 3.0 2.5 2.0
2 3 4 5 6 7 8 9 10

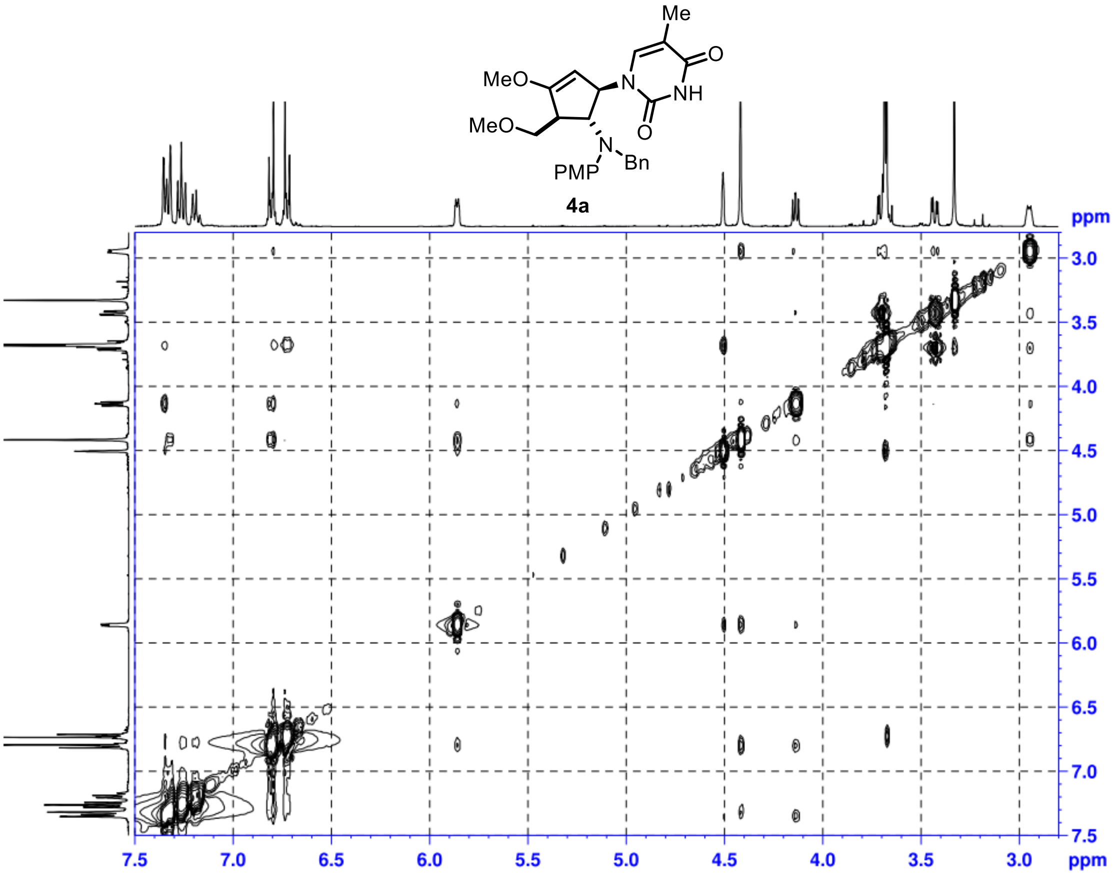

Me
O
MeO
N
NH
MeO
O
N
Bn
PMP
4a
ppm
7.5 7.0 6.5 6.0 5.5 5.0 4.5 4.0 3.5 3.0
3.0 3.5 4.0 4.5 5.0 5.5 6.0 6.5 7.0 7.5

R6-058-1p, 1H, BBFO1400, CD3CN, Dec-2013

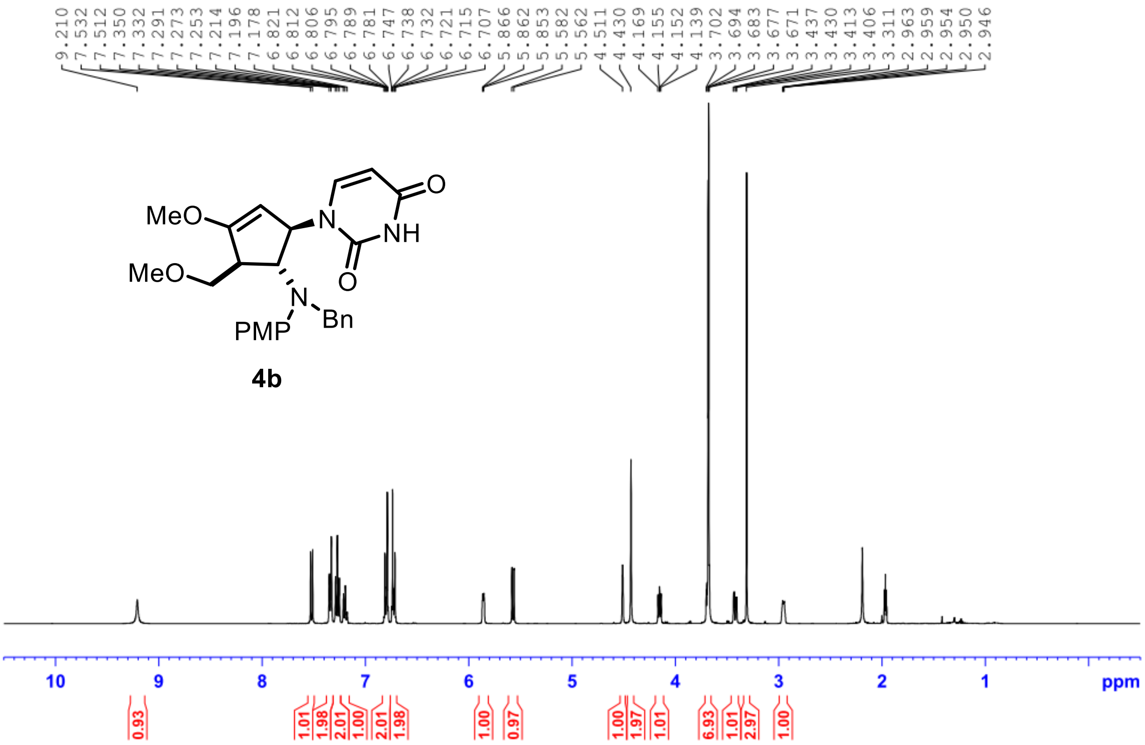

R6-058-1p, 13C, BBFO1400, CD3CN, Dec-2013

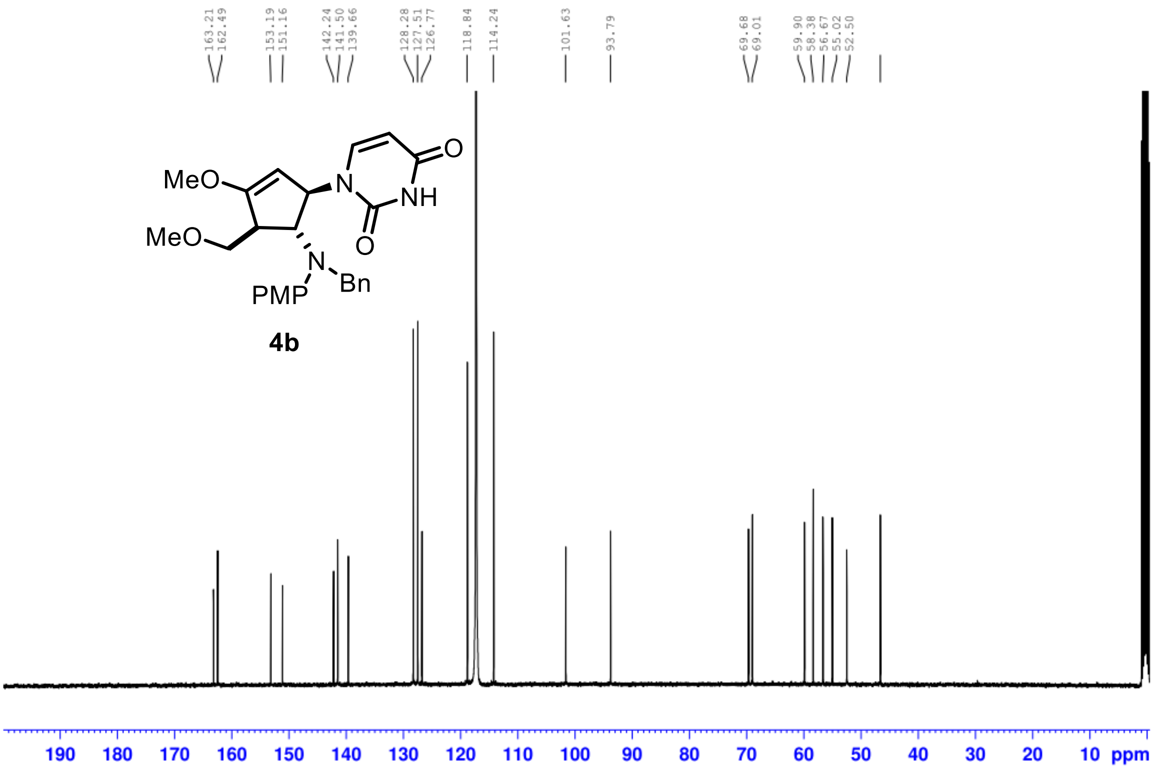

COSY of **4b**

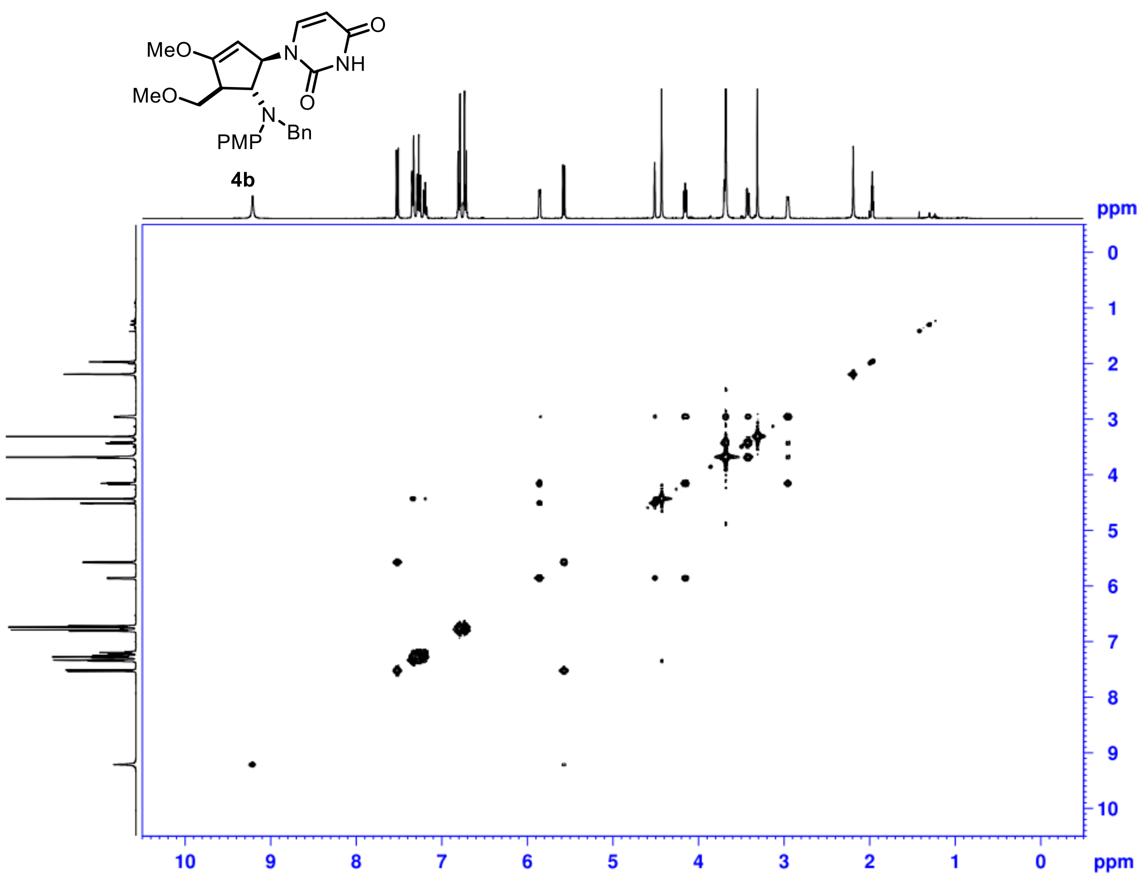
MeO
MeO
N
NH
O
O
N
Bn
PMP
4b
ppm
0
1
2
3
4
5
6
7
8
9
10
10
9
8
7
6
5
4
3
2
1
0
ppm

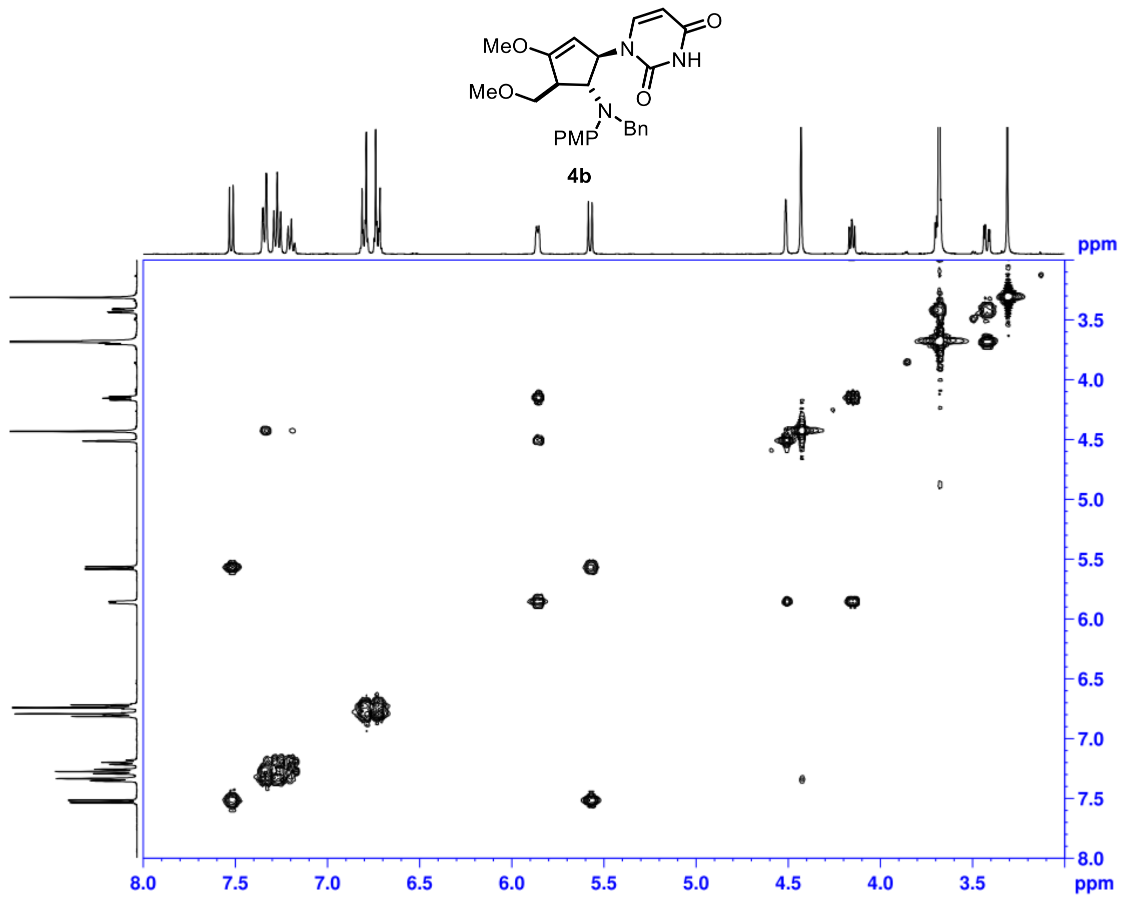
MeO
MeO
N
NH
O
O
N
Bn
PMP
4b
ppm
3.5
4.0
4.5
5.0
5.5
6.0
6.5
7.0
7.5
8.0
8.0
7.5
7.0
6.5
6.0
5.5
5.0
4.5
4.0
3.5
ppm

NOESY of **4b**

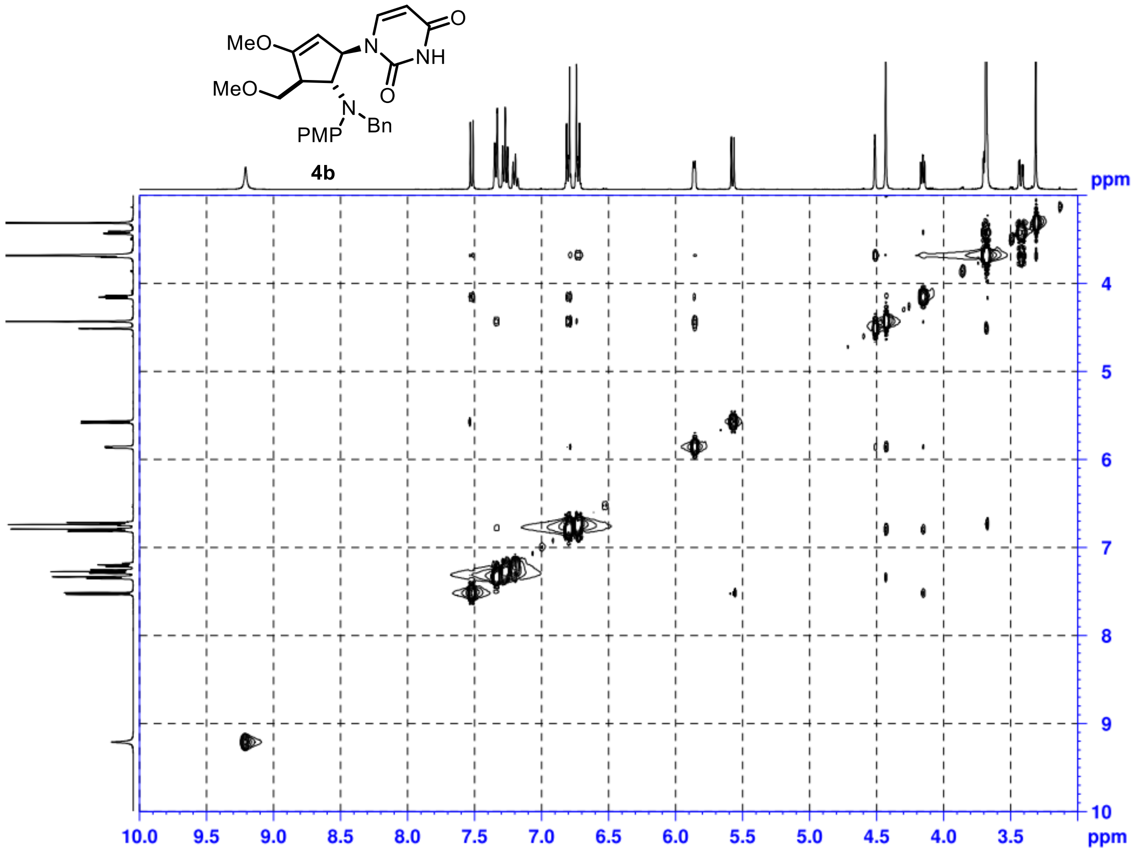
MeO
MeO
N
NH
O
O
N
PMP
Bn
4b
ppm
10.0
9.5
9.0
8.5
8.0
7.5
7.0
6.5
6.0
5.5
5.0
4.5
4.0
3.5
ppm
4
5
6
7
8
9
10

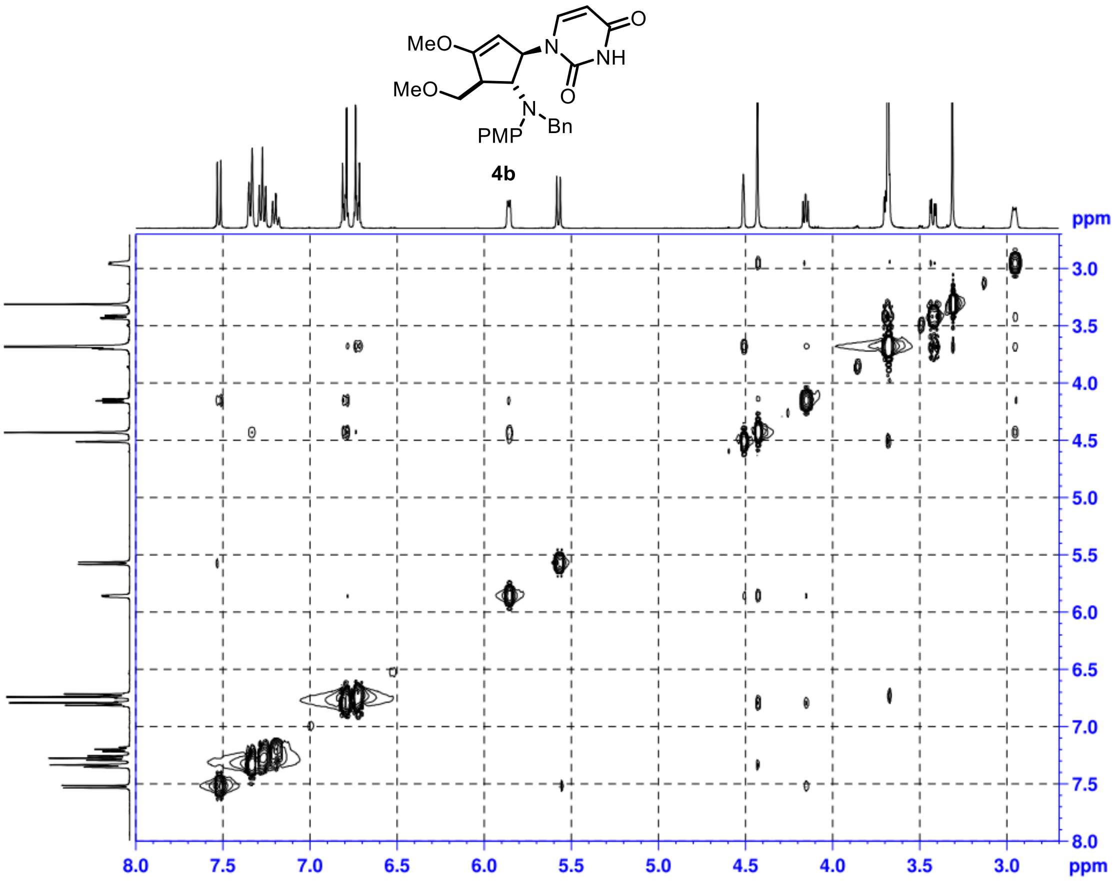
MeO
MeO
N
NH
O
O
N
PMP
Bn
4b
ppm
8.0
7.5
7.0
6.5
6.0
5.5
5.0
4.5
4.0
3.5
3.0
ppm
3.0
3.5
4.0
4.5
5.0
5.5
6.0
6.5
7.0
7.5
8.0

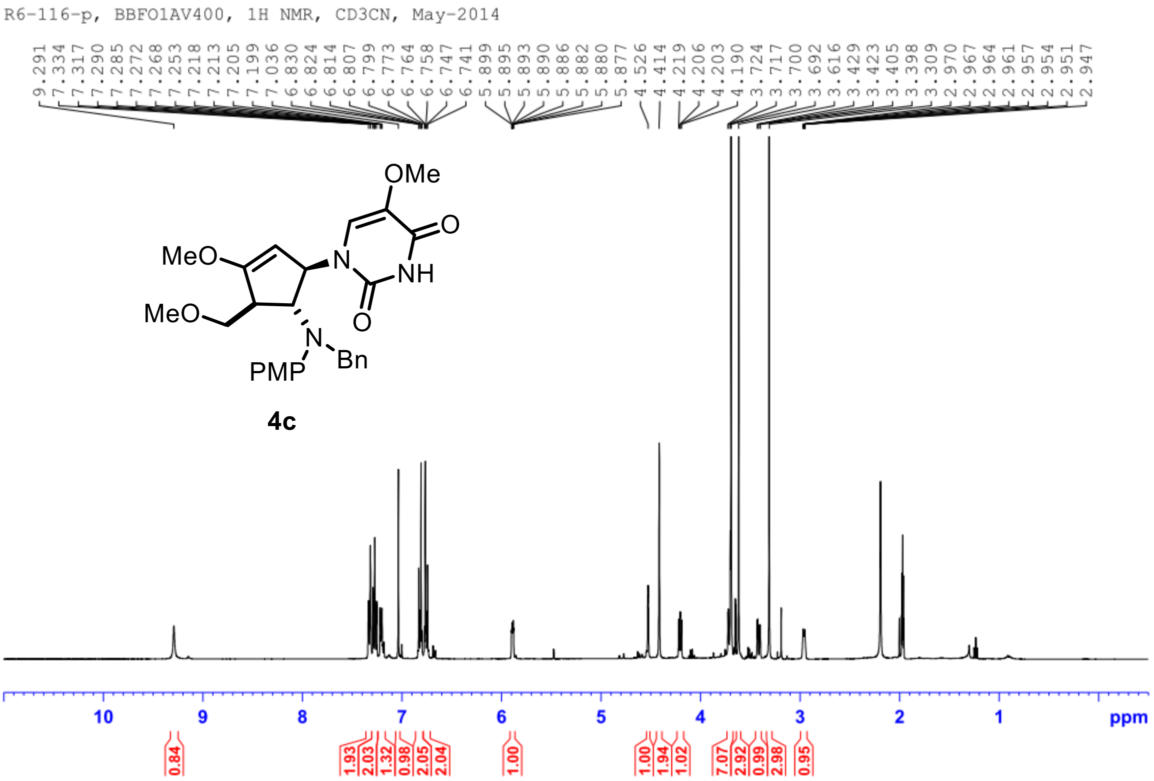
R6-116-p, BBFO1AV400, 1H NMR, CD3CN, May-2014
9.291 7.334 7.317 7.290 7.285 7.272 7.268 7.253 7.218 7.213 7.205 7.199 7.036 6.830 6.824 6.814 6.807 6.799 6.773 6.764 6.758 6.747 6.741 5.899 5.895 5.893 5.890 5.886 5.882 5.880 5.877 4.526 4.414 4.219 4.206 4.203 4.190 3.724 3.717 3.700 3.692 3.616 3.429 3.423 3.405 3.398 3.309 2.970 2.967 2.964 2.961 2.957 2.954 2.951 2.947
OMe
O
MeO
N
NH
MeO
O
N
Bn
PMP
4c
10 9 8 7 6 5 4 3 2 1 ppm
0.84 1.93 2.03 1.32 0.98 2.05 2.04 1.00 1.00 1.94 1.02 7.07 2.92 0.99 2.98 0.95

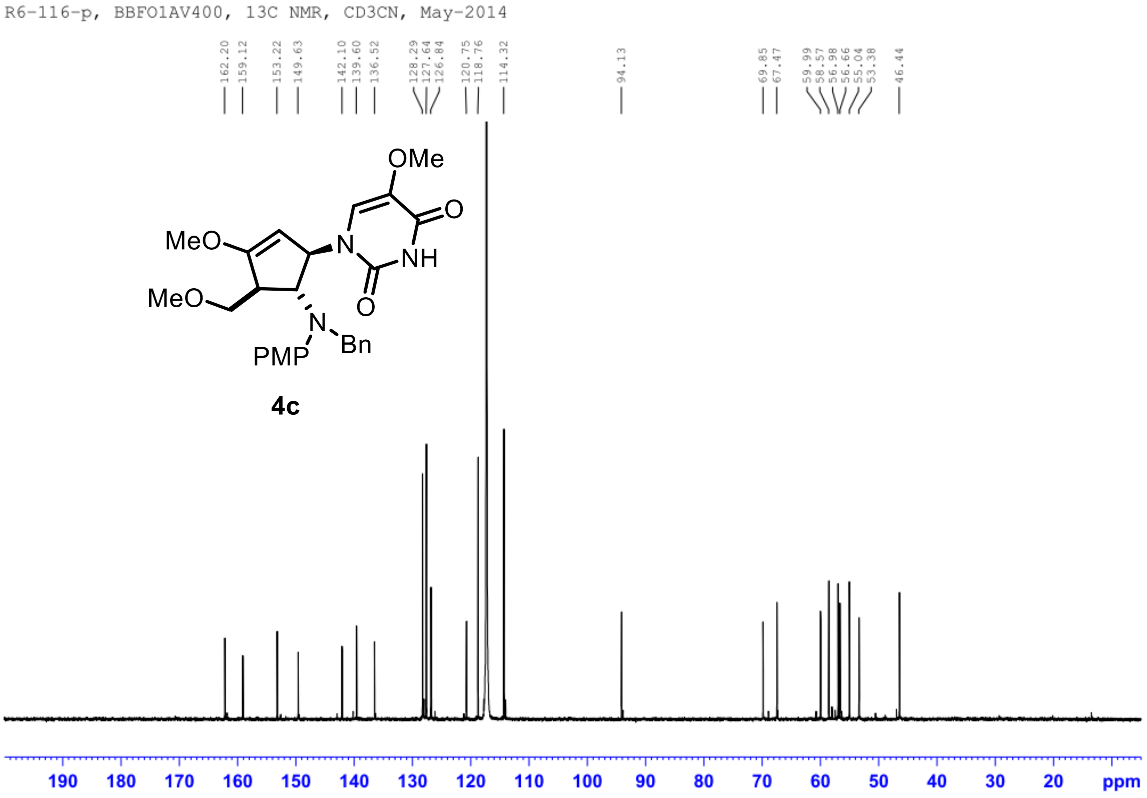
R6-116-p, BBFO1AV400, 13C NMR, CD3CN, May-2014
162.20 159.12 153.22 149.63 142.10 139.60 136.52 128.29 127.64 126.84 120.75 118.76 114.32 94.13 69.85 67.47 59.99 58.57 56.98 56.66 55.04 53.38 46.44
OMe
O
MeO
N
NH
MeO
O
N
Bn
PMP
4c
190 180 170 160 150 140 130 120 110 100 90 80 70 60 50 40 30 20 ppm

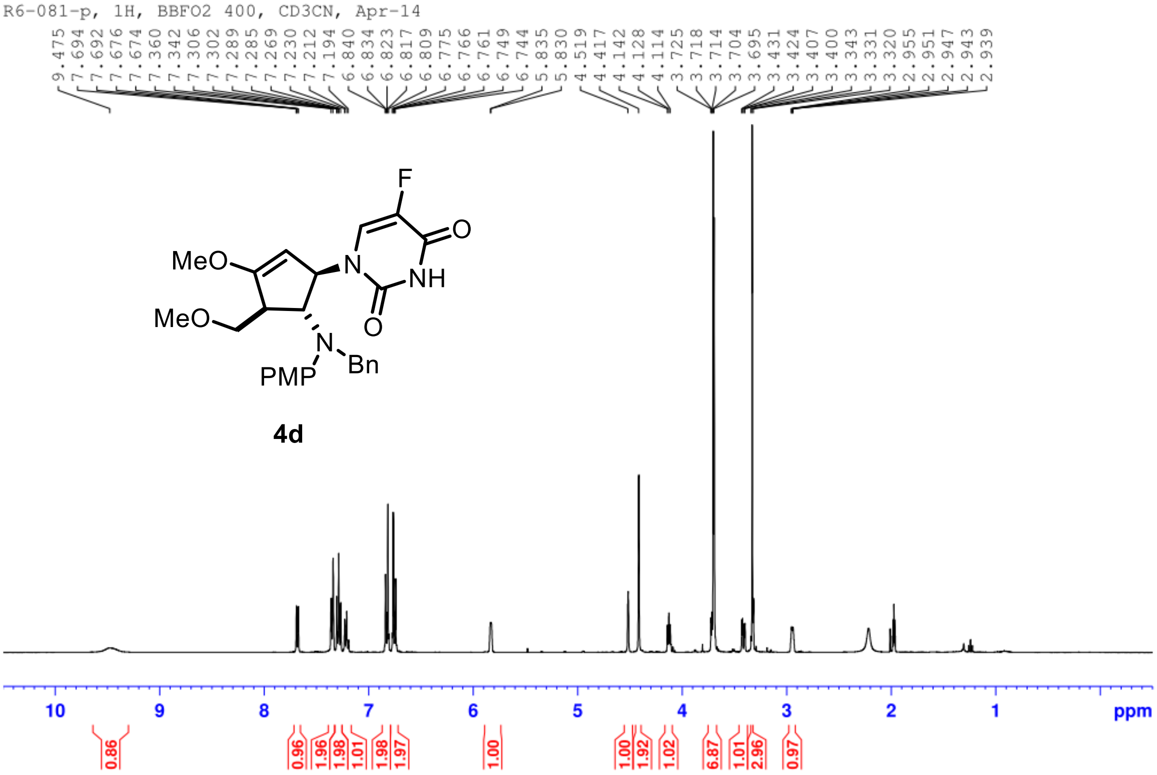
R6-081-p, 1H, BBFO2 400, CD3CN, Apr-14
9.475 7.694 7.692 7.676 7.674 7.360 7.342 7.306 7.302 7.289 7.285 7.269 7.230 7.212 7.194 6.840 6.834 6.823 6.817 6.809 6.775 6.766 6.761 6.749 6.744 5.835 5.830 4.519 4.417 4.142 4.128 4.114 3.725 3.718 3.714 3.704 3.695 3.431 3.424 3.407 3.400 3.343 3.331 3.320 2.955 2.951 2.947 2.943 2.939
F
O
MeO
N
NH
MeO
O
N
PMP
Bn
4d
10 9 8 7 6 5 4 3 2 1 ppm
0.86 0.96 1.96 1.98 1.01 1.98 1.97 1.00 1.00 1.92 1.02 6.87 1.01 2.96 0.97

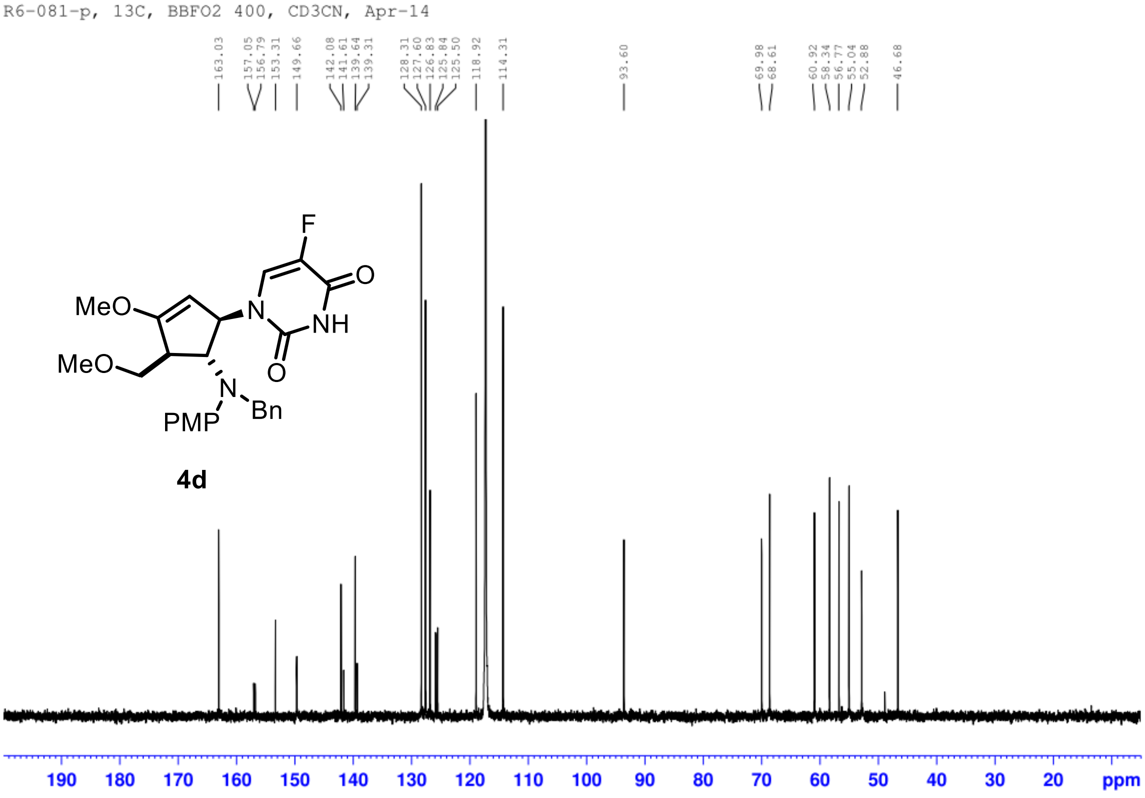
R6-081-p, 13C, BBFO2 400, CD3CN, Apr-14
163.03 157.05 156.79 153.31 149.66 142.08 141.61 139.64 139.31 128.31 127.60 126.83 125.84 125.50 118.92 114.31 93.60 69.98 68.61 60.92 58.34 56.77 55.04 52.88 46.68
F
O
MeO
N
NH
MeO
O
N
PMP
Bn
4d
190 180 170 160 150 140 130 120 110 100 90 80 70 60 50 40 30 20 ppm

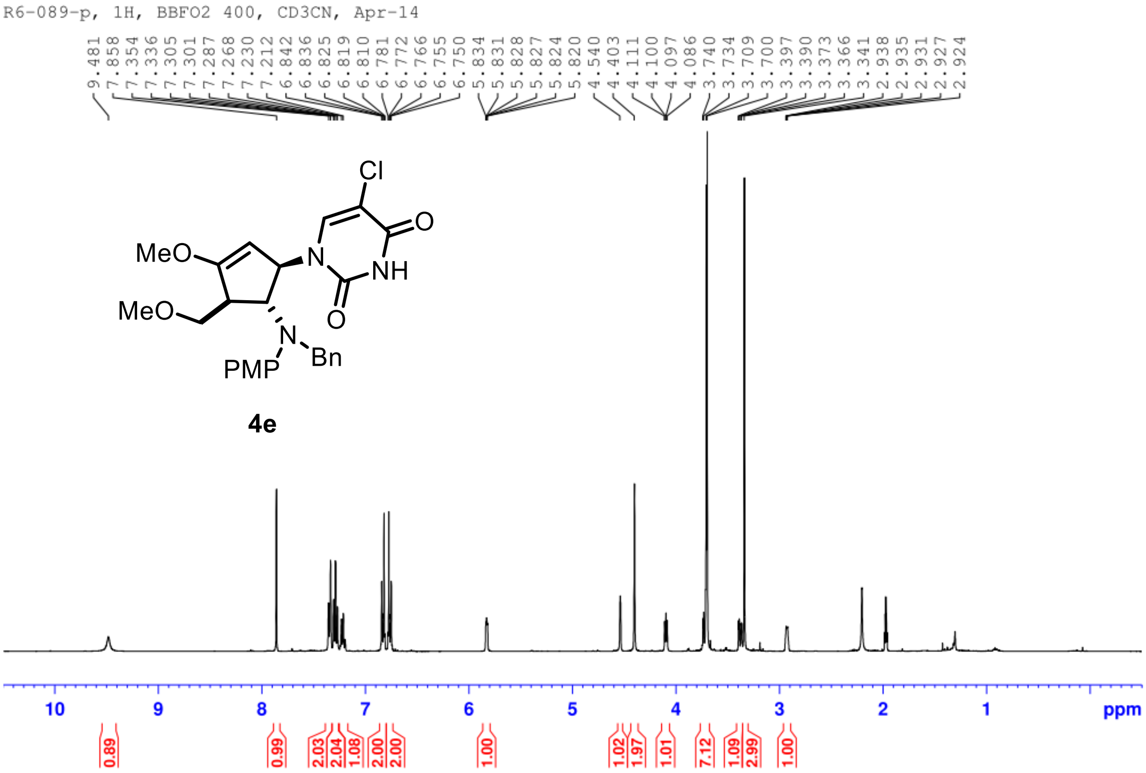
R6-089-p, 1H, BBFO2 400, CD3CN, Apr-14
9.481
7.858
7.354
7.336
7.305
7.301
7.287
7.268
7.230
7.212
6.842
6.836
6.825
6.819
6.810
6.781
6.772
6.766
6.755
6.750
5.834
5.831
5.828
5.827
5.824
5.820
4.540
4.403
4.111
4.100
4.097
4.086
3.740
3.734
3.709
3.700
3.397
3.390
3.373
3.366
3.341
2.938
2.935
2.931
2.927
2.924
Cl
O
MeO
N
NH
MeO
O
N
Bn
PMP
4e
10
9
8
7
6
5
4
3
2
1
ppm
0.89
0.99
2.03
2.04
1.08
2.00
2.00
1.00
1.02
1.97
1.01
7.12
1.09
2.99
1.00

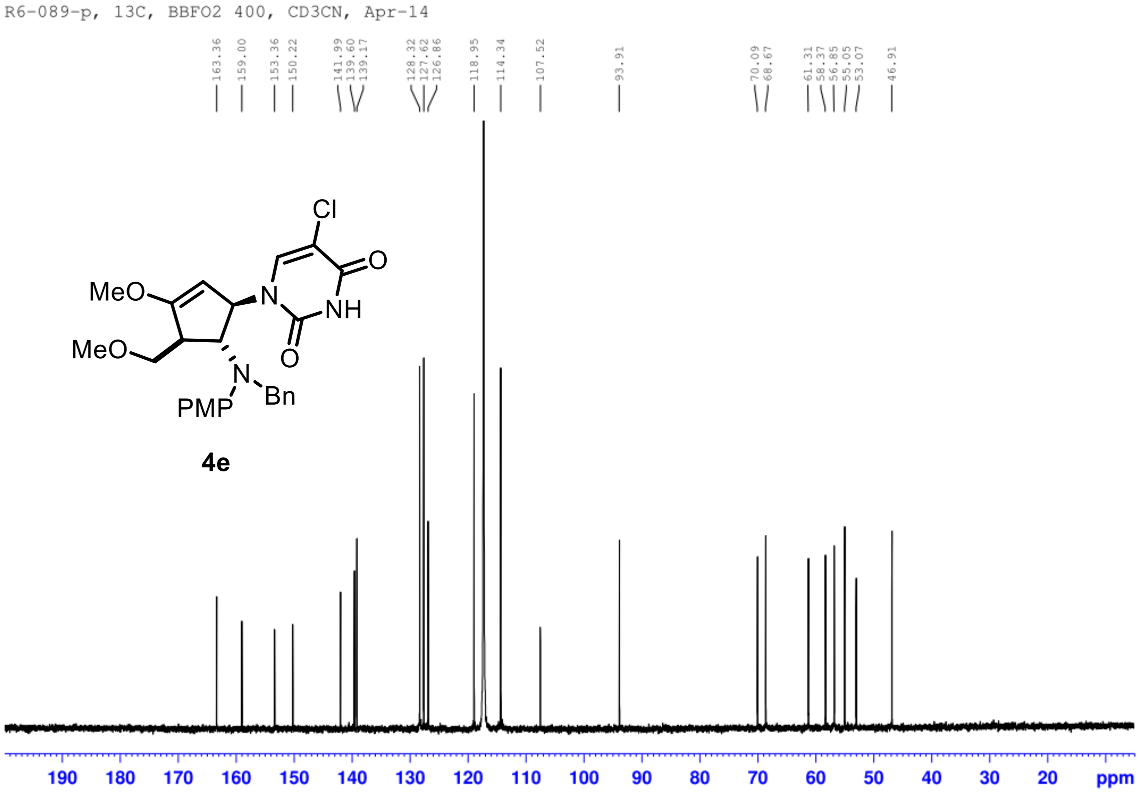
R6-089-p, 13C, BBFO2 400, CD3CN, Apr-14
163.36
159.00
153.36
150.22
141.99
139.60
139.17
128.32
127.62
126.86
118.95
114.34
107.52
93.91
70.09
68.67
61.31
58.37
56.85
55.05
53.07
46.91
Cl
O
MeO
N
NH
MeO
O
N
Bn
PMP
4e
190
180
170
160
150
140
130
120
110
100
90
80
70
60
50
40
30
20
ppm

R6-082-p, 1H, BBFO2 400, CD3CN, Apr-14

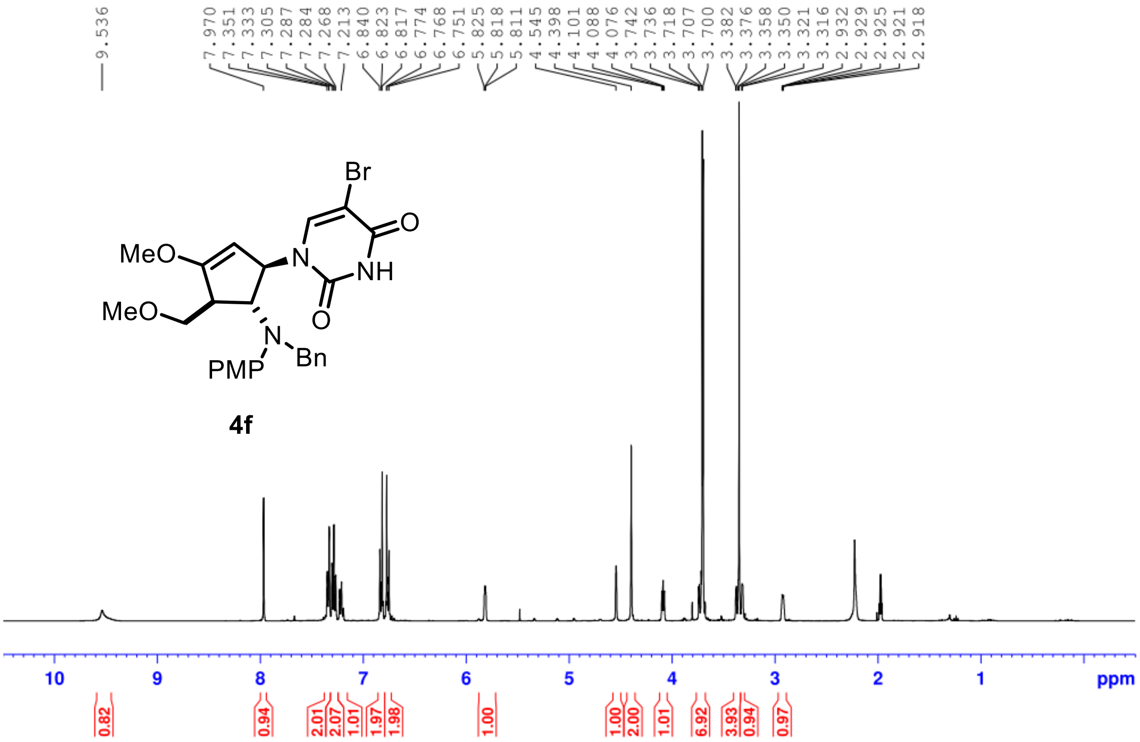

R6-082-p, 13C, BBFO2 400, CD3CN, Apr-14

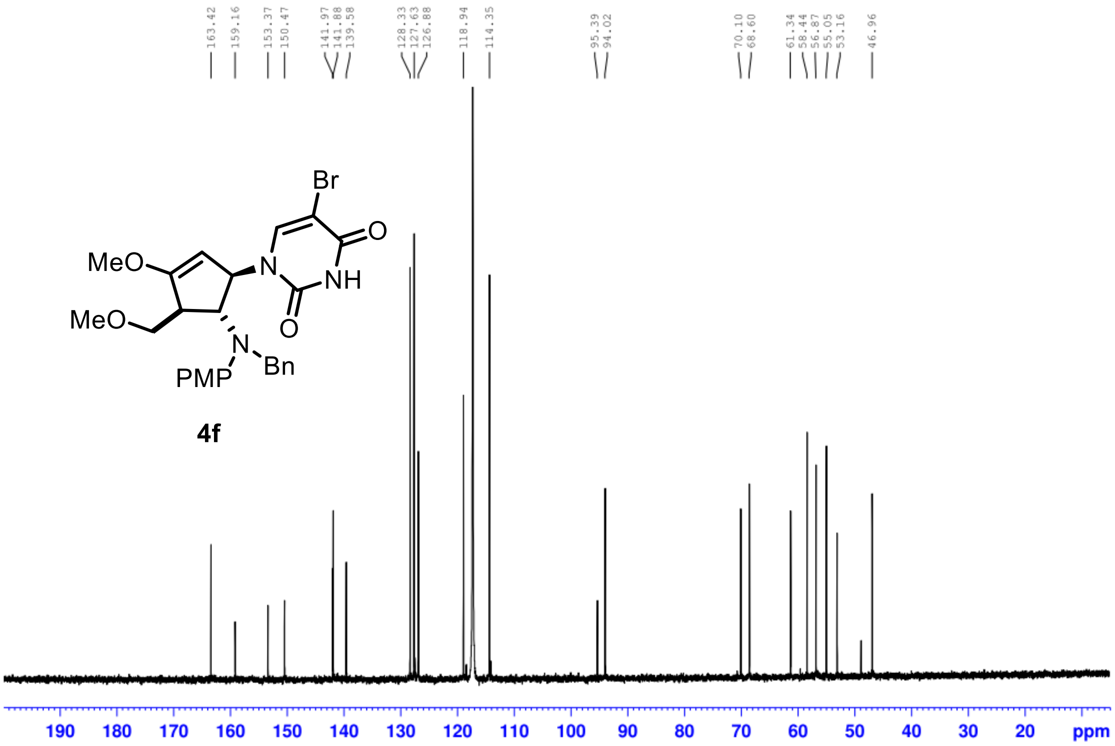

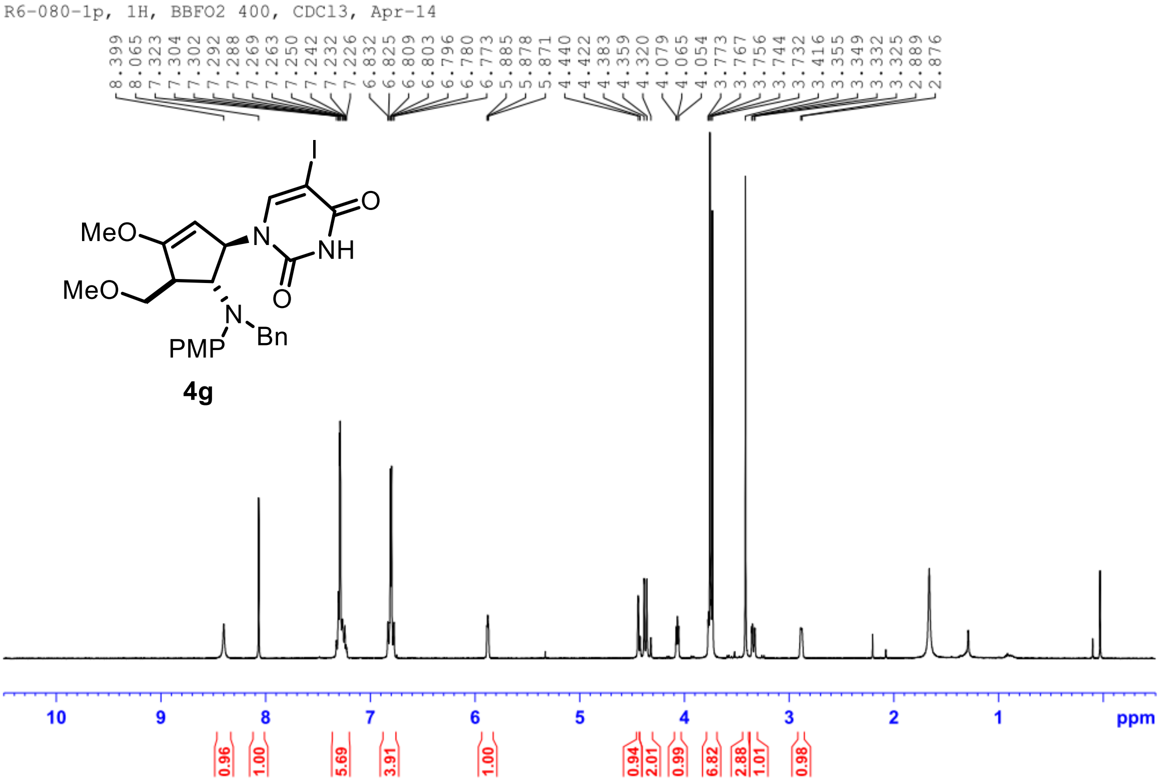
R6-080-1p, 1H, BBFO2 400, CDCl3, Apr-14
8.399 8.065 7.323 7.304 7.302 7.292 7.288 7.269 7.263 7.250 7.242 7.232 7.226 6.832 6.825 6.809 6.803 6.796 6.780 6.773 5.885 5.878 5.871 4.440 4.422 4.383 4.359 4.320 4.079 4.065 4.054 3.773 3.767 3.756 3.744 3.732 3.416 3.355 3.349 3.332 3.325 2.889 2.876
MeO
MeO
N
NH
O
O
I
N
Bn
PMP
4g
ppm
0.96 1.00 5.69 3.91 1.00 0.94 2.01 0.99 6.82 2.88 1.01 0.98

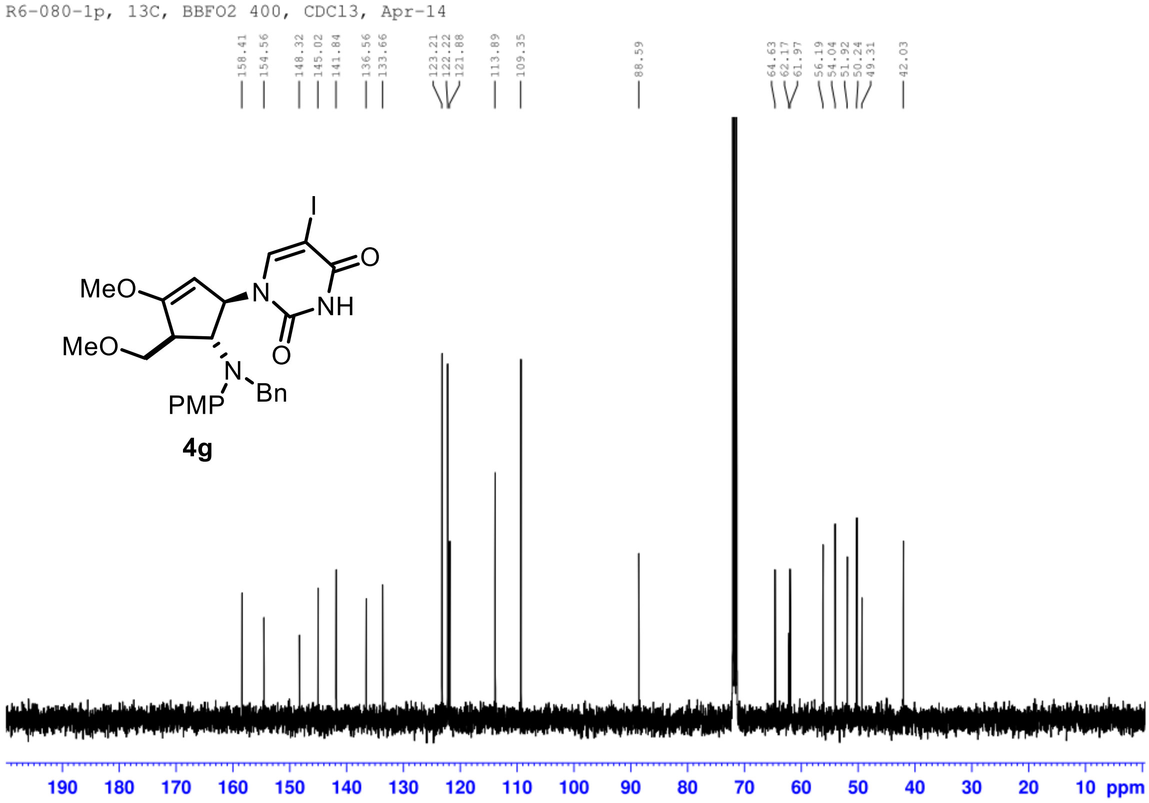
R6-080-1p, 13C, BBFO2 400, CDCl3, Apr-14
158.41 154.56 148.32 145.02 141.84 136.56 133.66 123.21 122.22 121.88 113.89 109.35 88.59 64.63 62.17 61.97 56.19 54.04 51.92 50.24 49.31 42.03
MeO
MeO
N
NH
O
O
I
N
Bn
PMP
4g
ppm

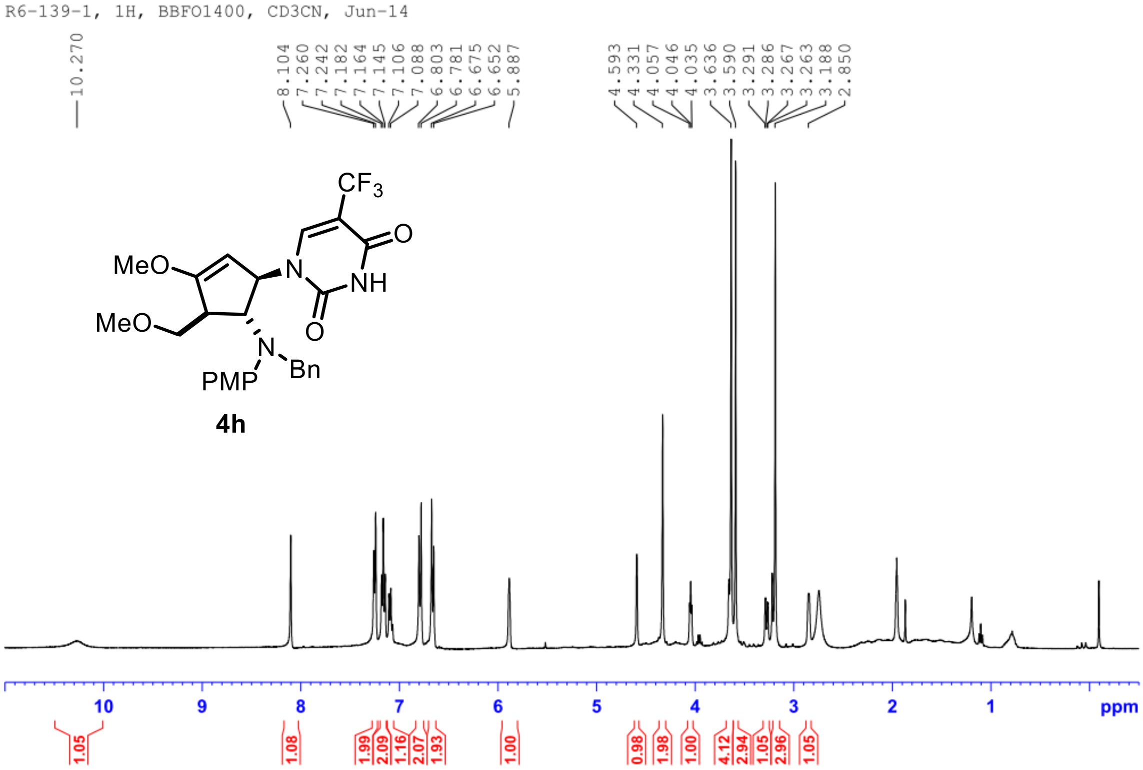

R6-139-1, 1H, BBFO1400, CD3CN, Jun-14
10.270
8.104
7.260
7.242
7.182
7.164
7.145
7.106
7.088
6.803
6.781
6.675
6.652
5.887
4.593
4.331
4.057
4.046
4.035
3.636
3.590
3.291
3.286
3.267
3.263
3.188
2.850
CF3
MeO
MeO
N
NH
O
O
N
Bn
PMP
4h
ppm
1.05
1.08
1.99
2.09
1.16
2.07
1.93
1.00
0.98
1.98
1.00
4.12
2.94
1.05
2.96
1.05

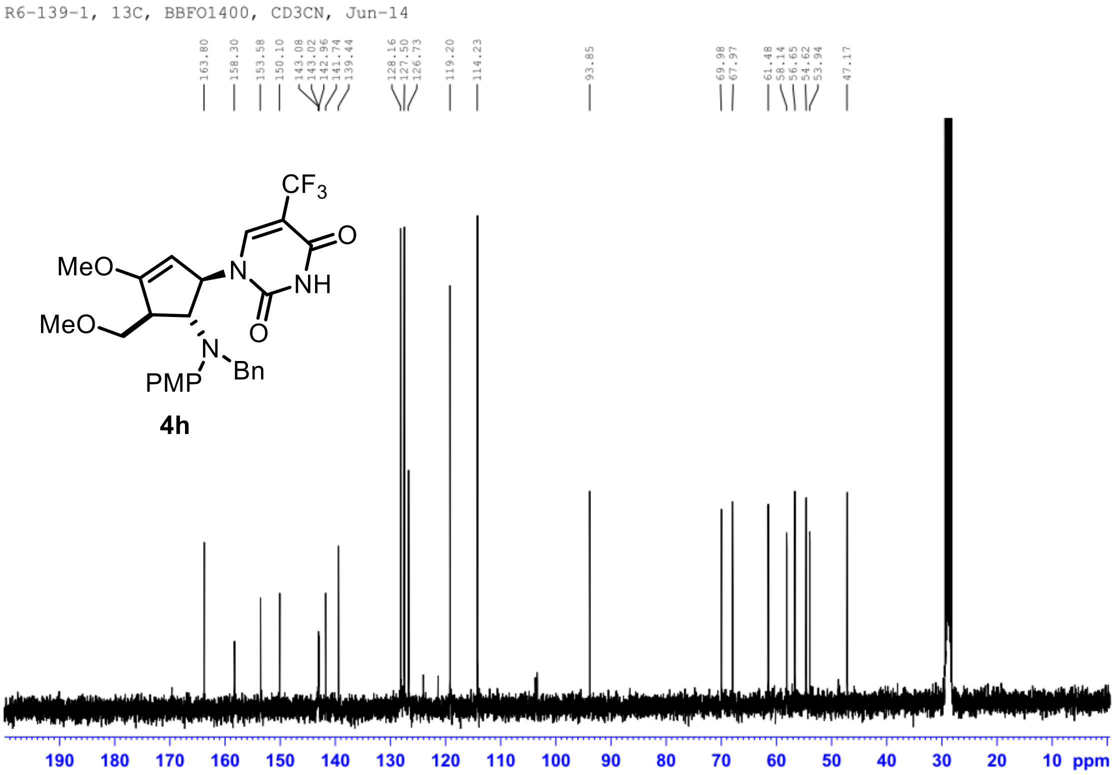

R6-139-1, 13C, BBFO1400, CD3CN, Jun-14
163.80
158.30
153.58
150.10
143.08
143.02
142.96
141.74
139.44
128.16
127.50
126.73
119.20
114.23
93.85
69.98
67.97
61.48
58.14
56.65
54.62
53.94
47.17
CF3
MeO
MeO
N
NH
O
O
N
Bn
PMP
4h
ppm

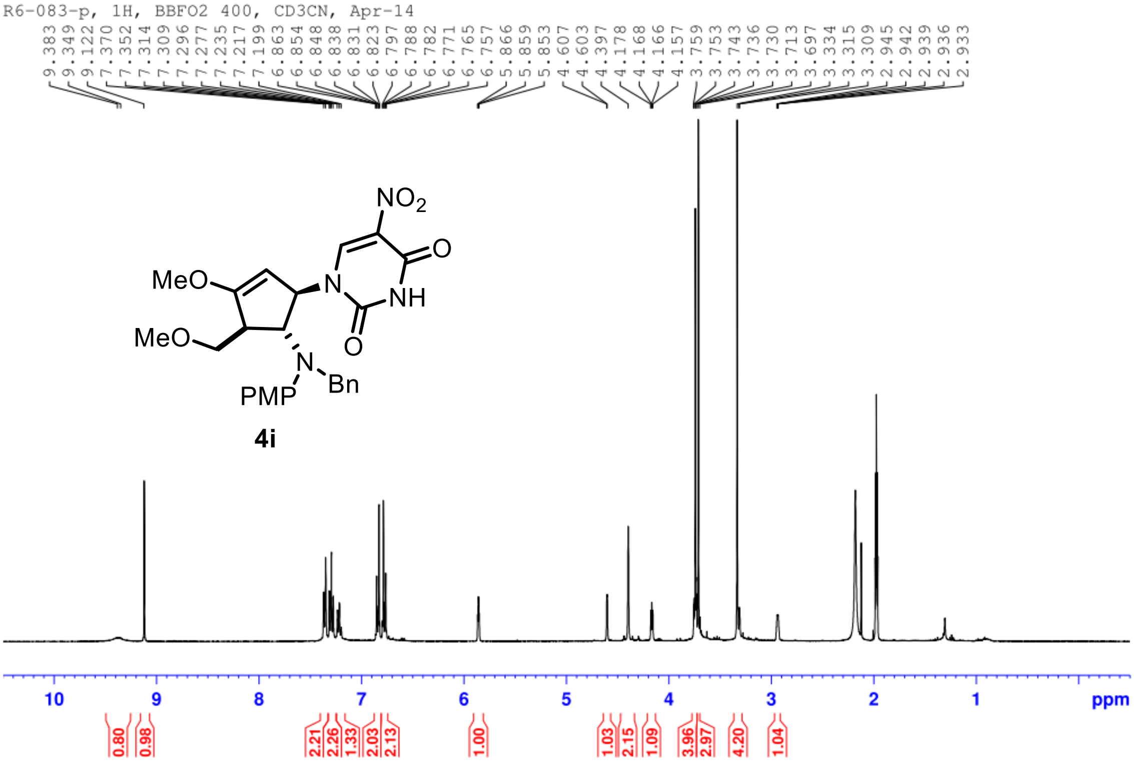
R6-083-p, 1H, BBFO2 400, CD3CN, Apr-14
9.383
9.349
9.122
7.370
7.352
7.314
7.309
7.296
7.277
7.235
7.217
7.199
6.863
6.854
6.848
6.838
6.831
6.823
6.797
6.788
6.782
6.771
6.765
6.757
5.866
5.859
5.853
4.607
4.603
4.397
4.178
4.168
4.166
4.157
3.759
3.753
3.743
3.736
3.730
3.713
3.697
3.334
3.315
3.309
2.945
2.942
2.939
2.936
2.933
NO2
MeO
MeO
N
NH
O
O
N
Bn
PMP
4i
10
9
8
7
6
5
4
3
2
1
ppm
0.80
0.98
2.21
2.26
1.33
2.03
2.13
1.00
1.03
2.15
1.09
3.96
2.97
4.20
1.04

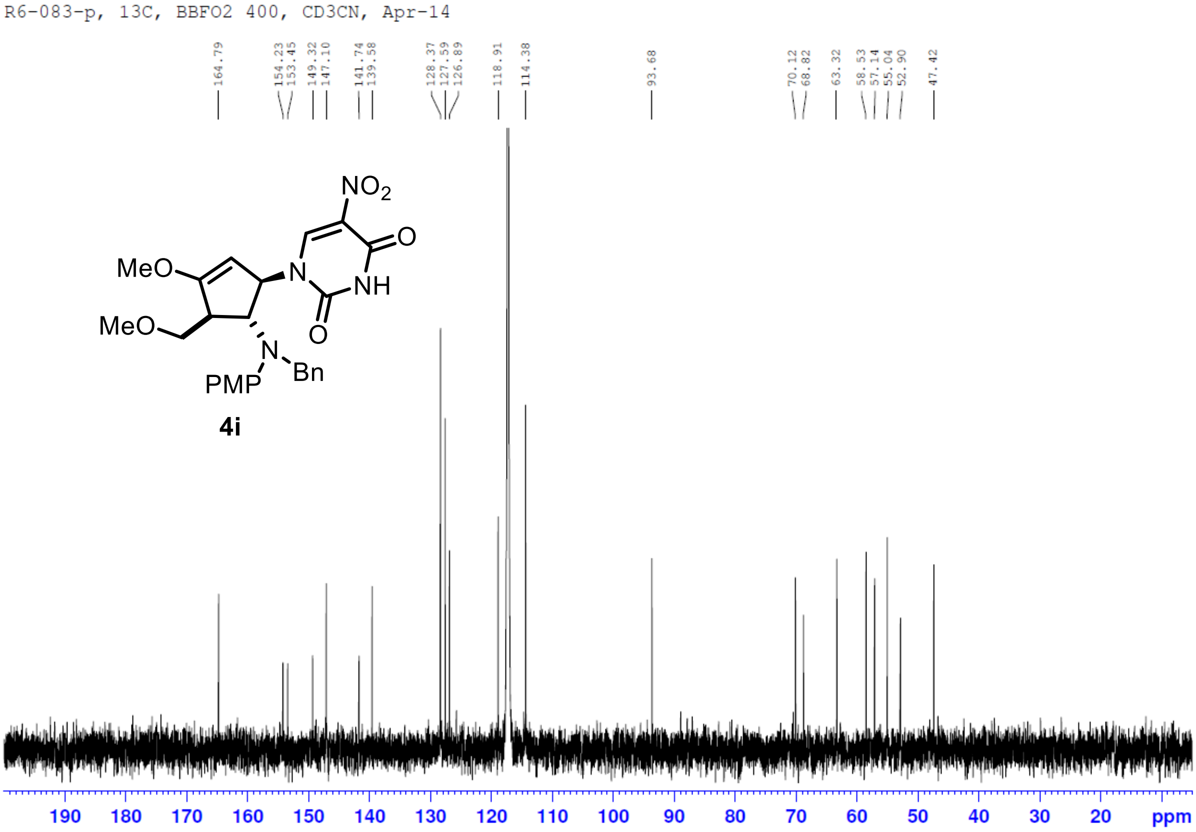
R6-083-p, 13C, BBFO2 400, CD3CN, Apr-14
164.79
154.23
153.45
149.32
147.10
141.74
139.58
128.37
127.59
126.89
118.91
114.38
93.68
70.12
68.82
63.32
58.53
57.14
55.04
52.90
47.42
NO2
MeO
MeO
N
NH
O
O
N
Bn
PMP
4i
190
180
170
160
150
140
130
120
110
100
90
80
70
60
50
40
30
20
ppm

R6-084-p, 1H, BBFO2 400, CD3CN, Apr-14

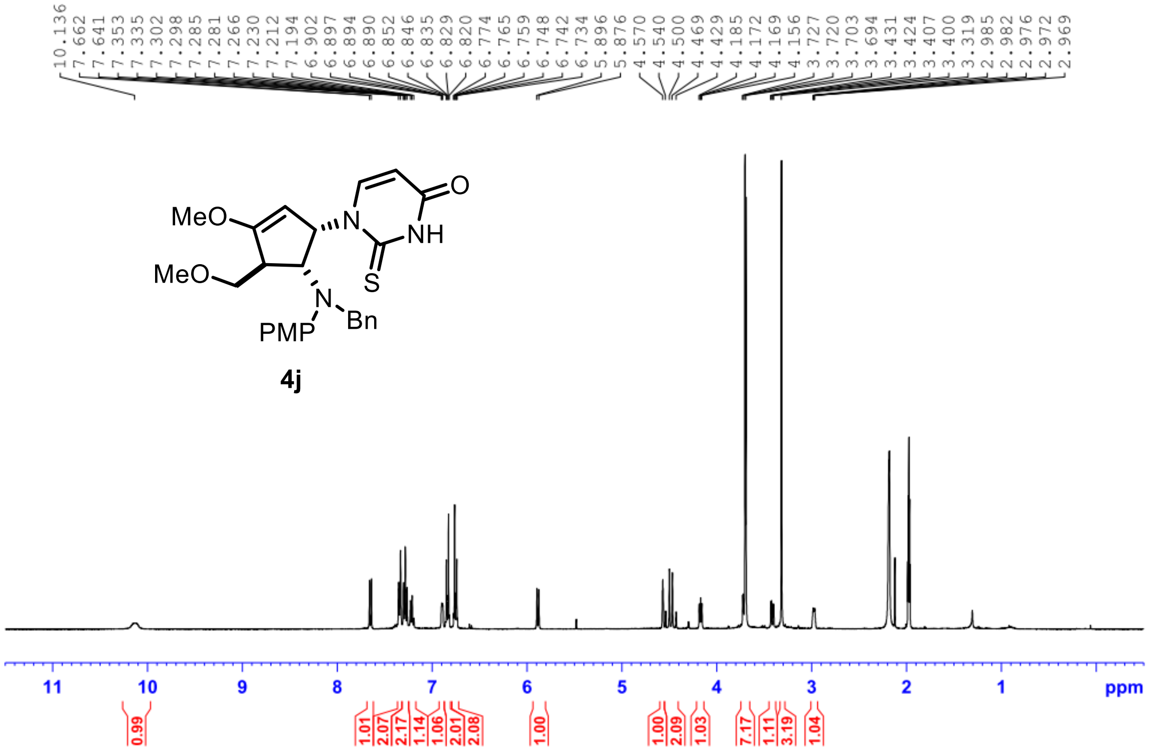

R6-084-p, 13C, BBFO2 400, CD3CN, Apr-14

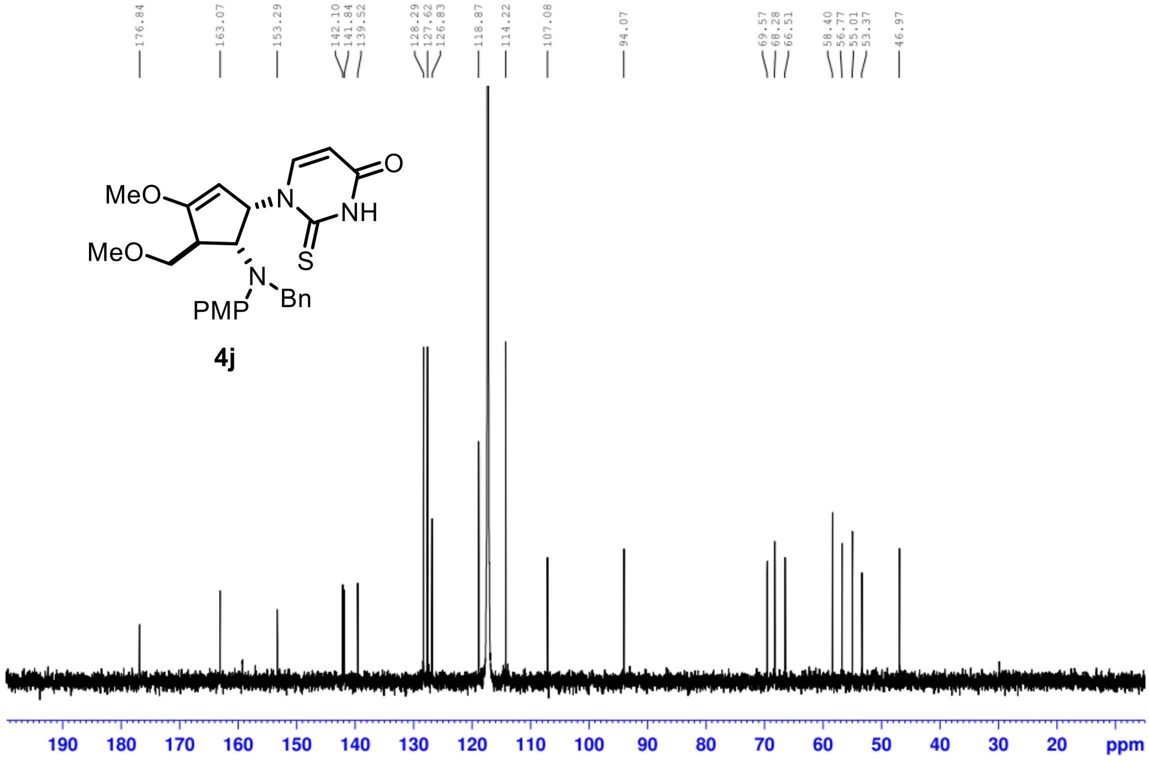

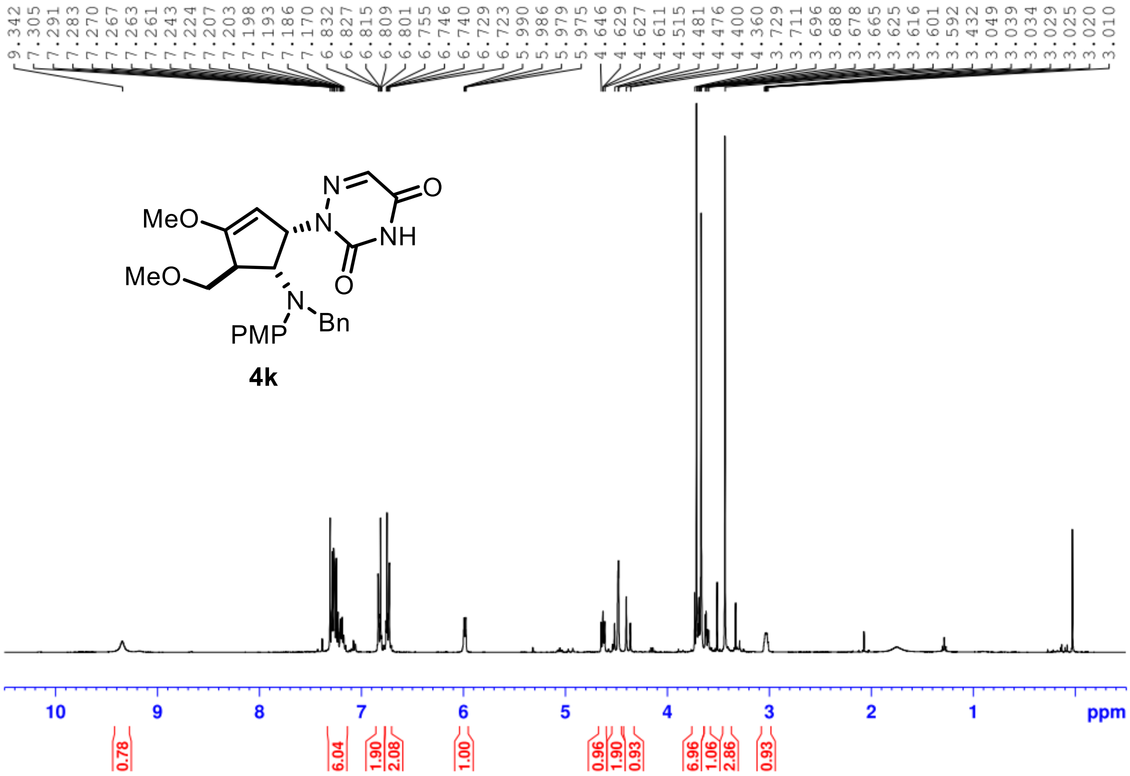

R6-140-3, 13C, BBFO1400, CDCl3, Jun-14

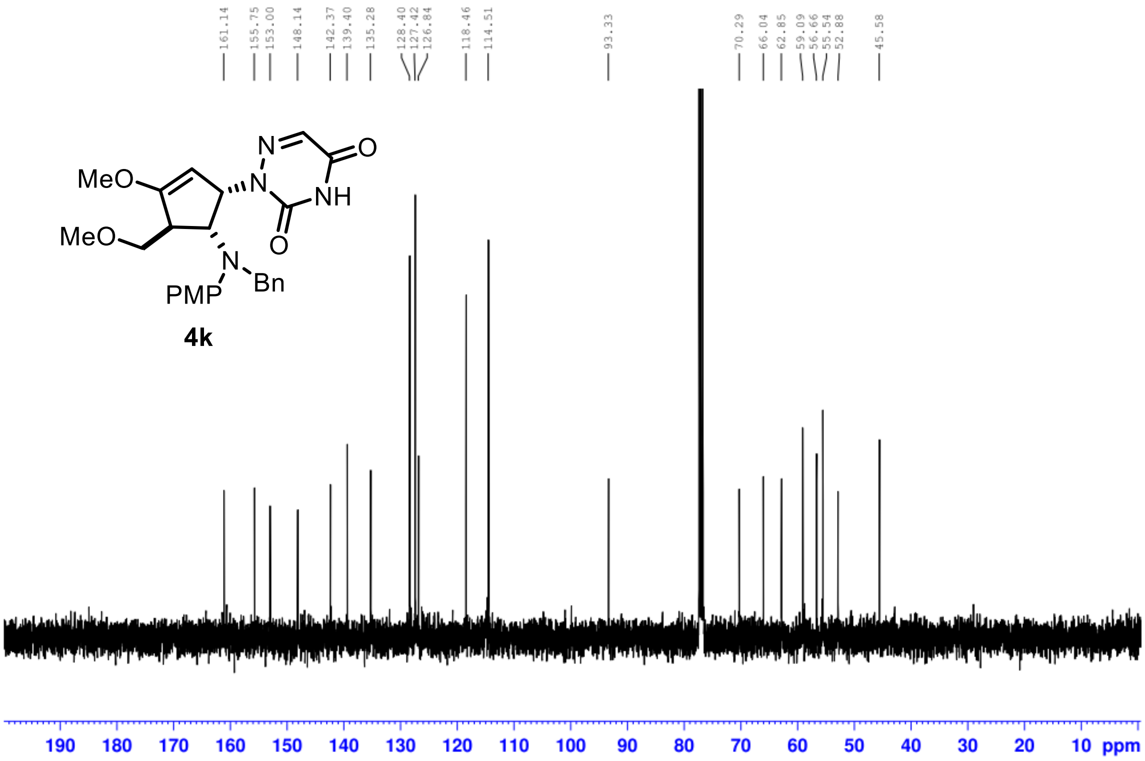

R6-125-p, 1H, BBFO2 400, CD3CN, Jun-14

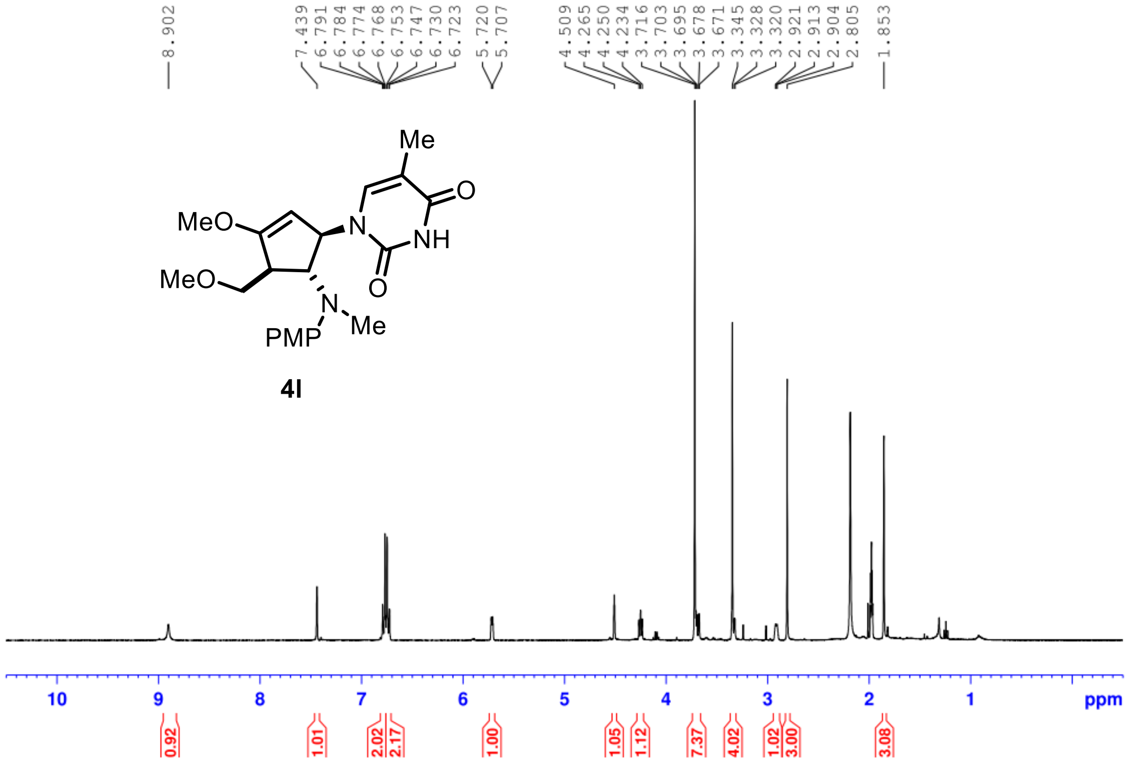

R6-125-p, 13C, BBFO2 400, CD3CN, Jun-14

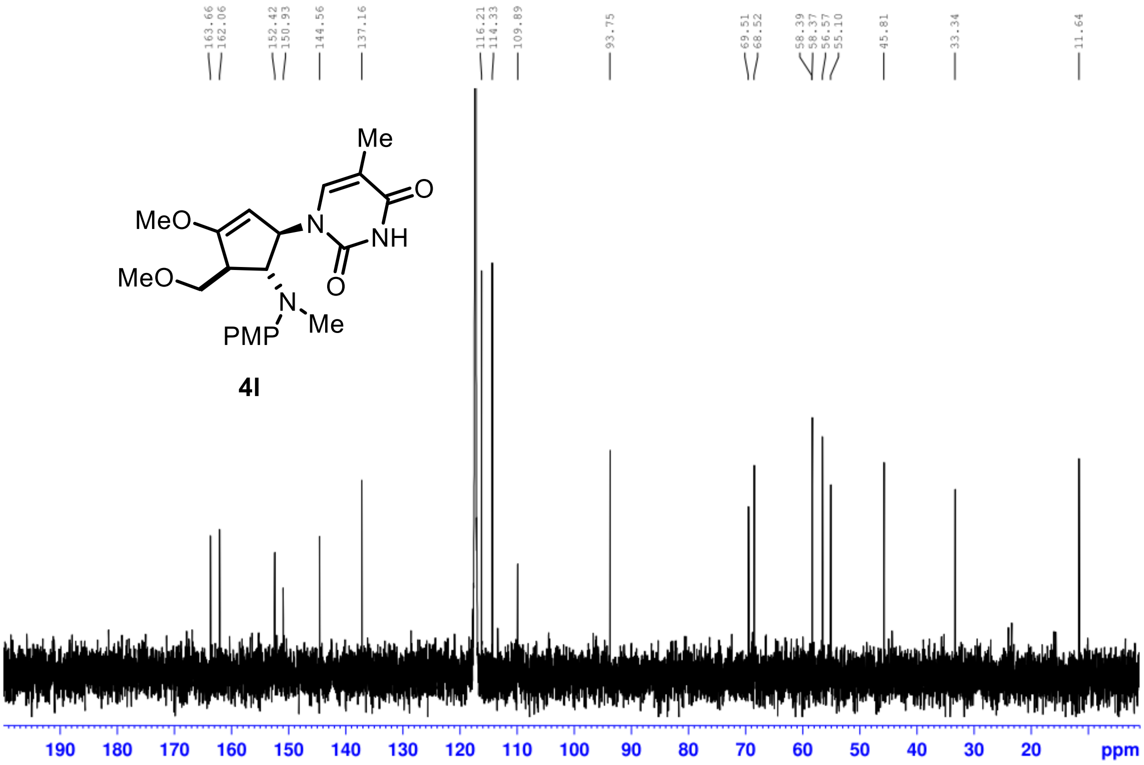

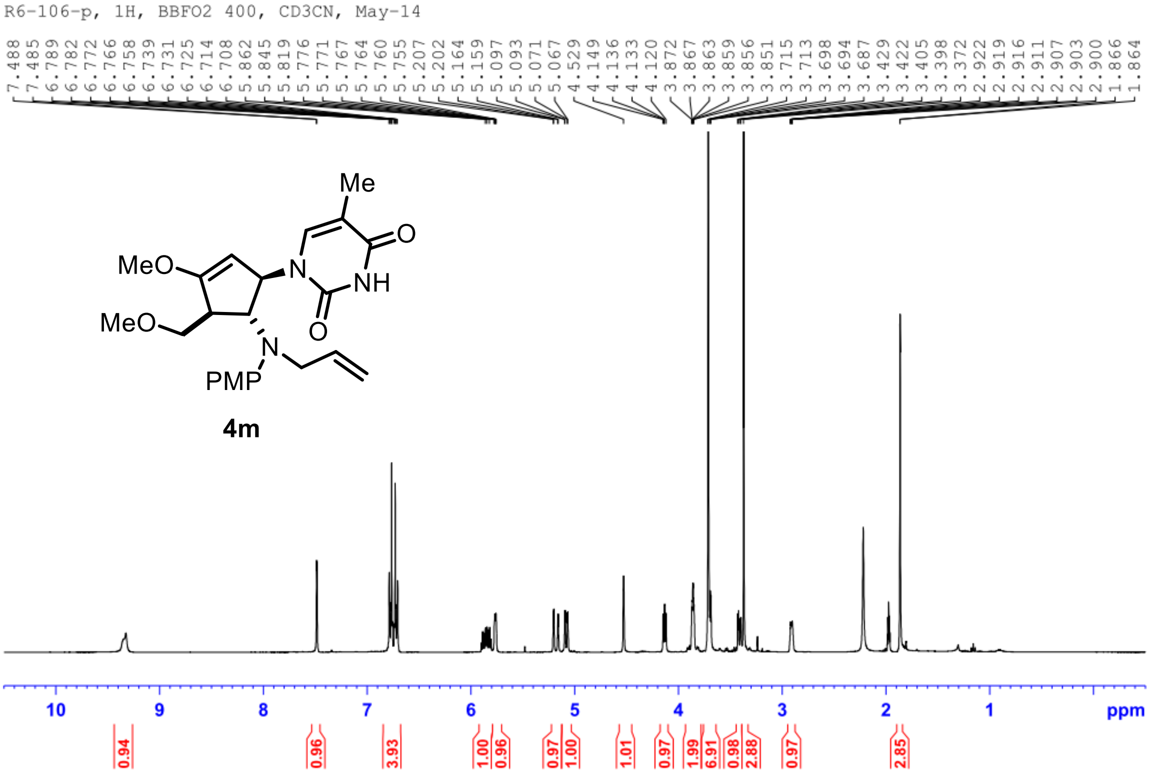
R6-106-p, 1H, BBFO2 400, CD3CN, May-14
7.488 7.485 6.789 6.782 6.772 6.766 6.758 6.739 6.731 6.725 6.714 6.708 5.862 5.845 5.819 5.776 5.771 5.767 5.764 5.760 5.755 5.207 5.202 5.164 5.159 5.097 5.093 5.071 5.067 4.529 4.149 4.136 4.133 4.120 3.872 3.867 3.863 3.859 3.856 3.851 3.715 3.713 3.698 3.694 3.687 3.429 3.422 3.405 3.398 3.372 2.922 2.919 2.916 2.911 2.907 2.903 2.900 1.866 1.864
Me
MeO
MeO
N
NH
O
O
N
PMP
4m
0.94 0.96 3.93 1.00 0.96 0.97 1.00 1.01 0.97 1.99 6.91 0.98 2.88 0.97 2.85
10 9 8 7 6 5 4 3 2 1 ppm

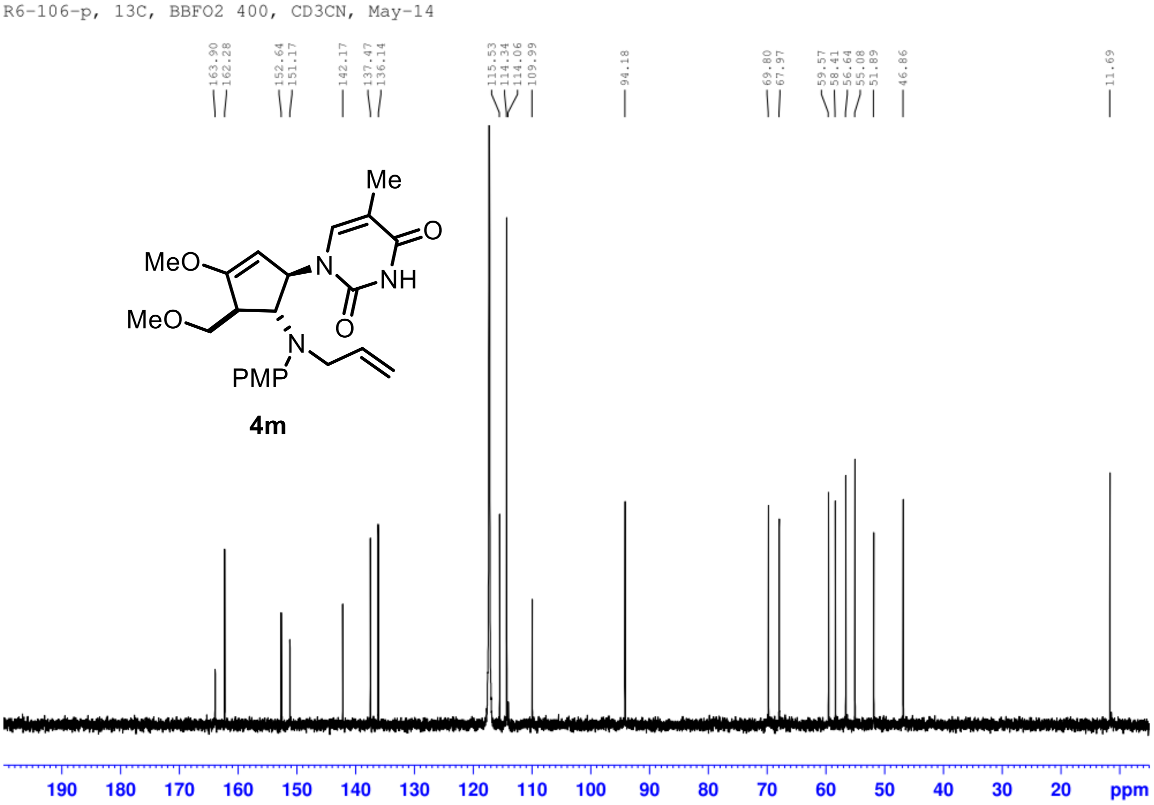
R6-106-p, 13C, BBFO2 400, CD3CN, May-14
163.90 162.28 152.64 151.17 142.17 137.47 136.14 115.53 114.34 114.06 109.99 94.18 69.80 67.97 59.57 58.41 56.64 55.08 51.89 46.86 11.69
Me
MeO
MeO
N
NH
O
O
N
PMP
4m
190 180 170 160 150 140 130 120 110 100 90 80 70 60 50 40 30 20 ppm

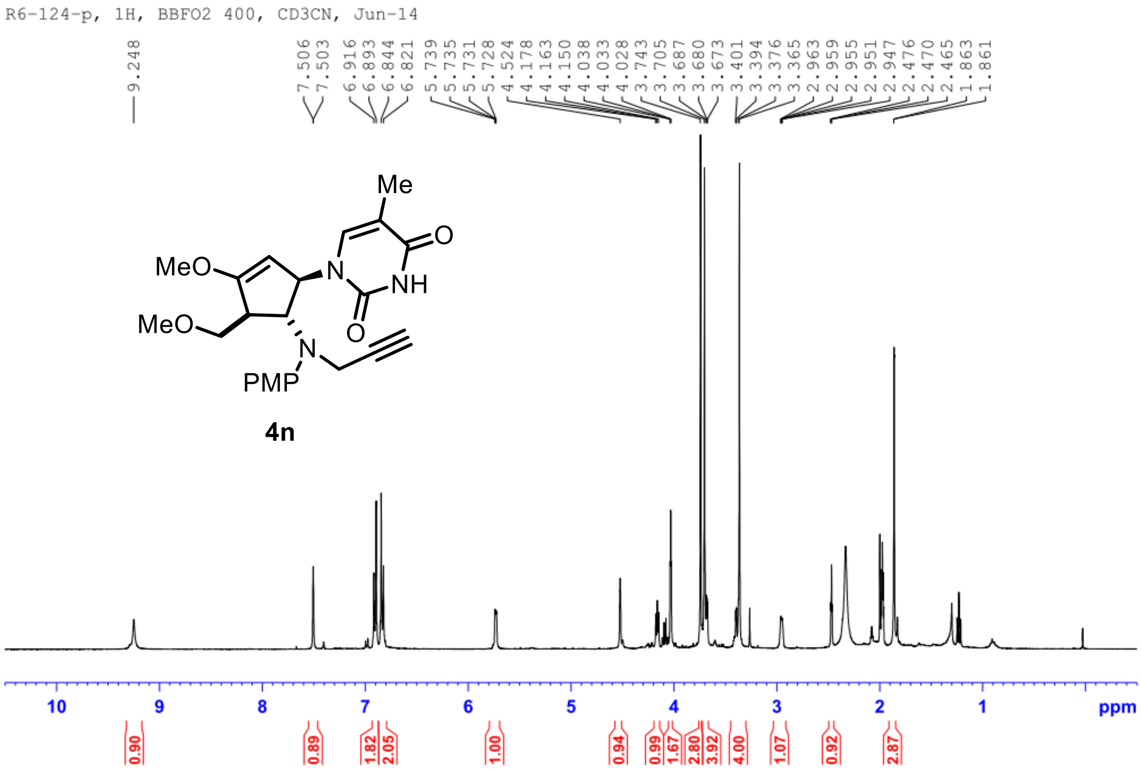
R6-124-p, 1H, BBFO2 400, CD3CN, Jun-14
9.248
7.506
7.503
6.916
6.893
6.844
6.821
5.739
5.735
5.731
5.728
4.524
4.178
4.163
4.150
4.038
4.033
4.028
3.743
3.705
3.687
3.680
3.673
3.401
3.394
3.376
3.365
2.963
2.959
2.955
2.951
2.947
2.476
2.470
2.465
1.863
1.861
Me
O
MeO
N
NH
MeO
O
N
PMP
4n
10 9 8 7 6 5 4 3 2 1 ppm
0.90
0.89
1.82
2.05
1.00
0.94
0.99
1.67
2.80
3.92
4.00
1.07
0.92
2.87

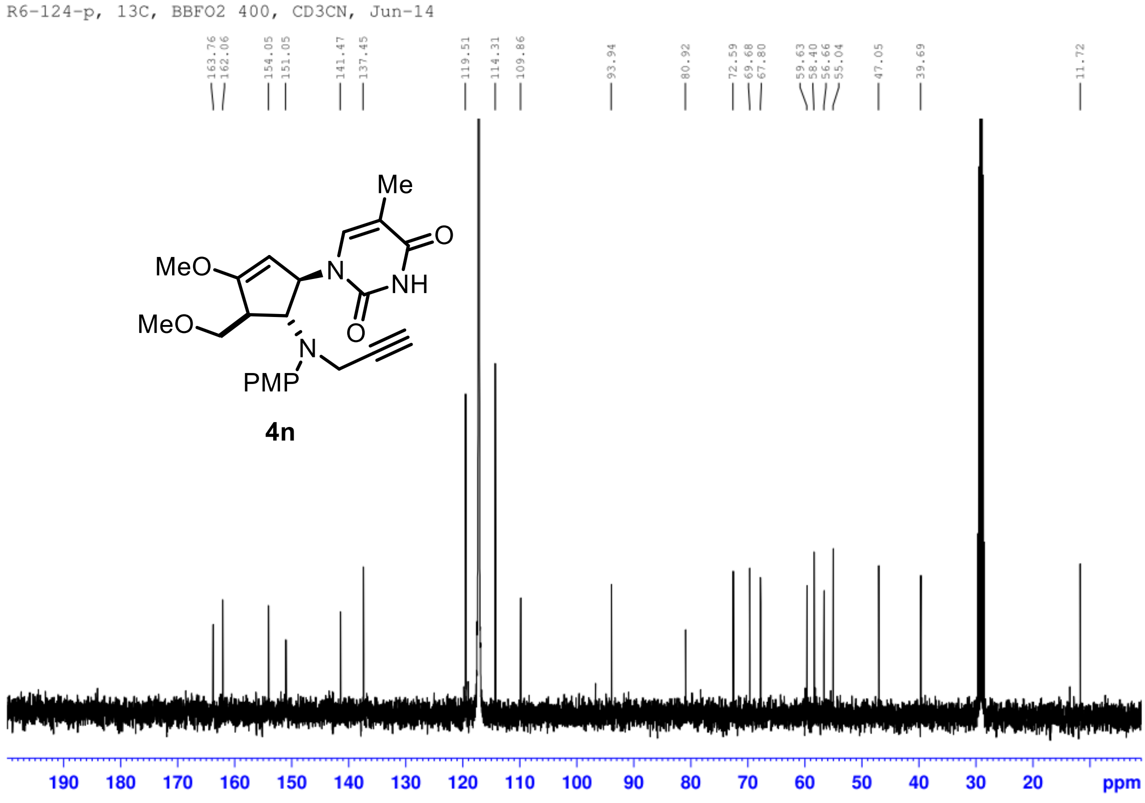
R6-124-p, 13C, BBFO2 400, CD3CN, Jun-14
163.76
162.06
154.05
151.05
141.47
137.45
119.51
114.31
109.86
93.94
80.92
72.59
69.68
67.80
59.63
58.40
56.66
55.04
47.05
39.69
11.72
Me
O
MeO
N
NH
MeO
O
N
PMP
4n
190 180 170 160 150 140 130 120 110 100 90 80 70 60 50 40 30 20 ppm

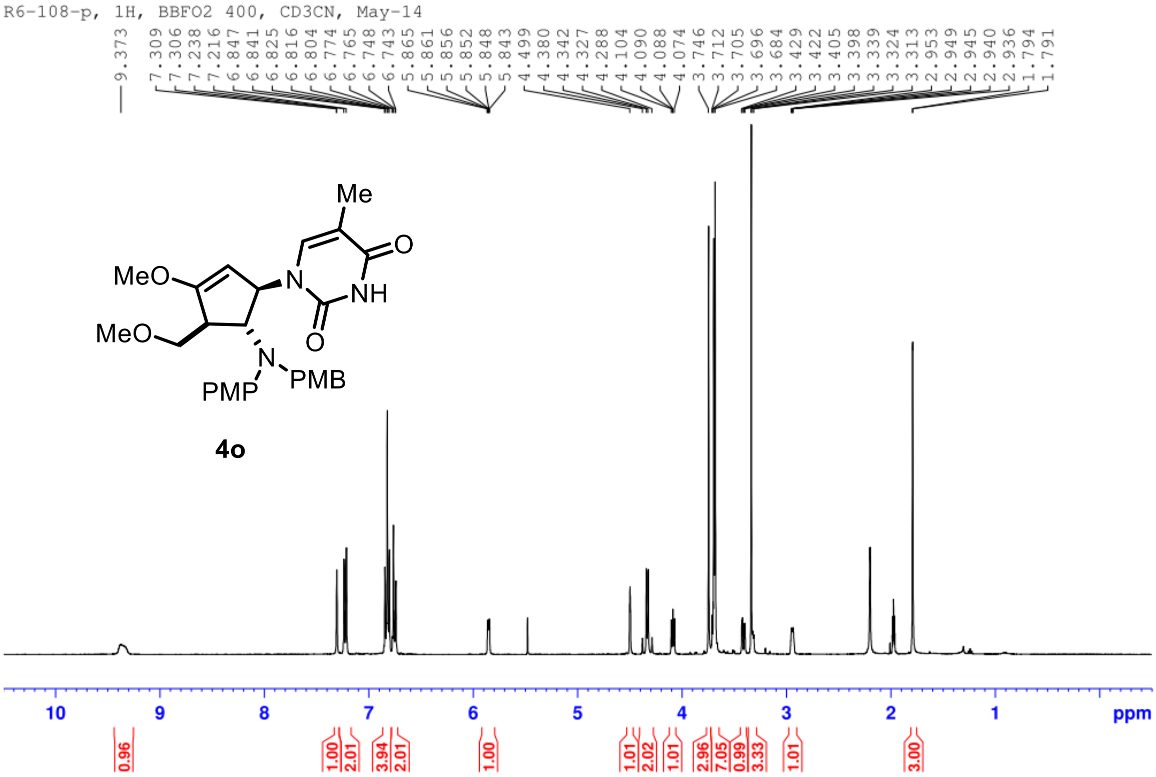
R6-108-p, 1H, BBFO2 400, CD3CN, May-14
9.373
7.309 7.306 7.238 7.216 6.847 6.841 6.825 6.816 6.804 6.774 6.765 6.748 6.743 5.865 5.861 5.856 5.852 5.848 5.843 4.499 4.380 4.342 4.327 4.288 4.104 4.090 4.088 4.074 3.746 3.712 3.705 3.696 3.684 3.429 3.422 3.405 3.398 3.339 3.324 3.313 2.953 2.949 2.945 2.940 2.936 1.794 1.791
Me
O
MeO
N
NH
MeO
O
N
PMP
PMB
4o
10 9 8 7 6 5 4 3 2 1 ppm
0.96
1.00 2.01 3.94 2.01
1.00
1.01 2.02 1.01 2.96 7.05 0.99 3.33 1.01
3.00

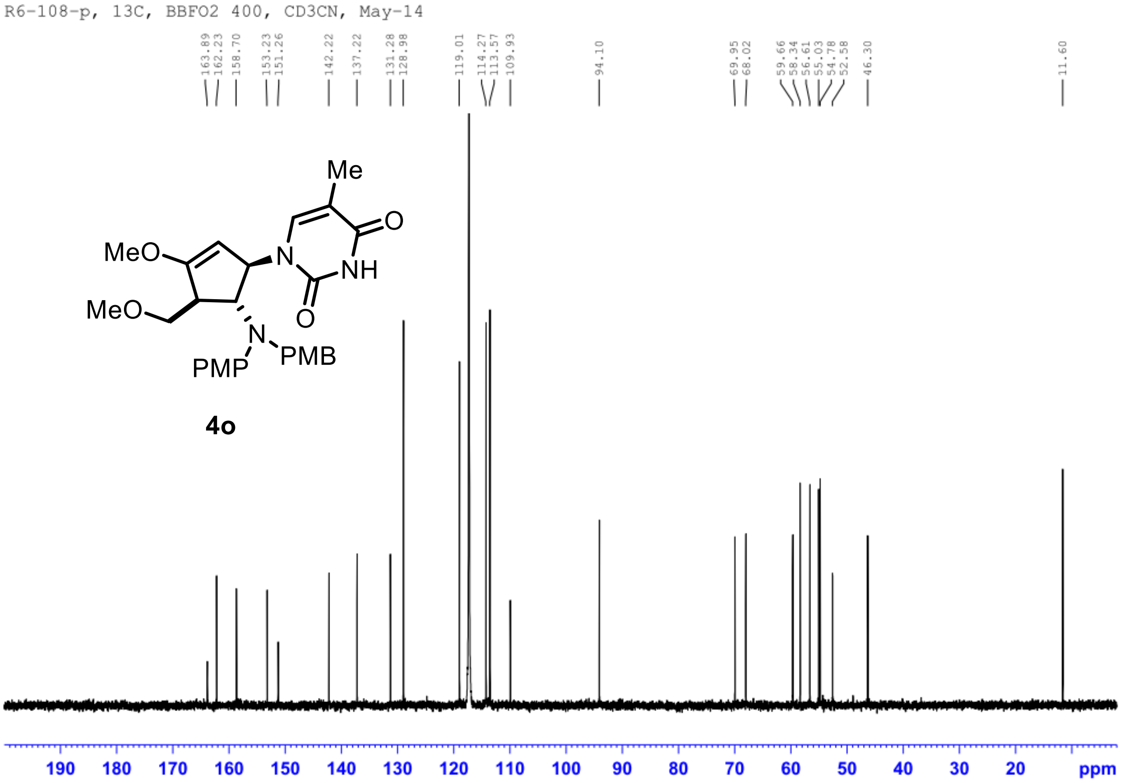
R6-108-p, 13C, BBFO2 400, CD3CN, May-14
163.89 162.23 158.70 153.23 151.26 142.22 137.22 131.28 128.98 119.01 114.27 113.57 109.93 94.10 69.95 68.02 59.66 58.34 56.61 55.03 54.78 52.58 46.30 11.60
Me
O
MeO
N
NH
MeO
O
N
PMP
PMB
4o
190 180 170 160 150 140 130 120 110 100 90 80 70 60 50 40 30 20 ppm

R6-109-p, 1H, BBFO2 400, CD3CN, May-14

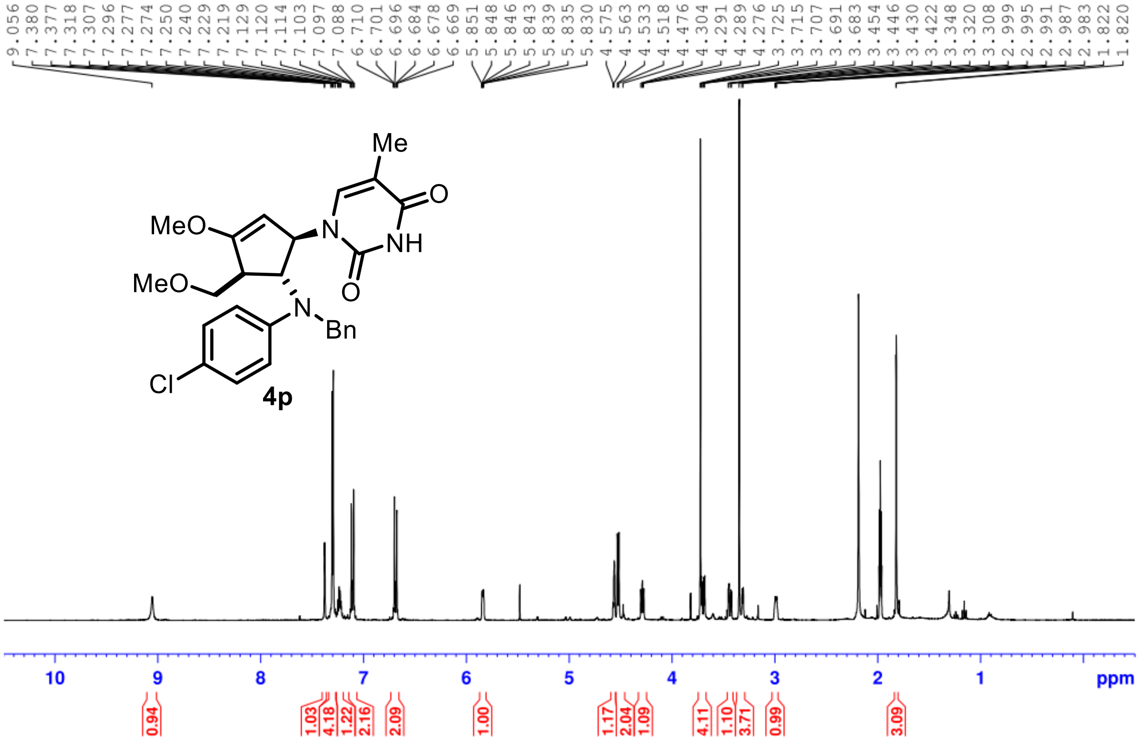

R6-109-p, 13C, BBFO2 400, CD3CN, May-14

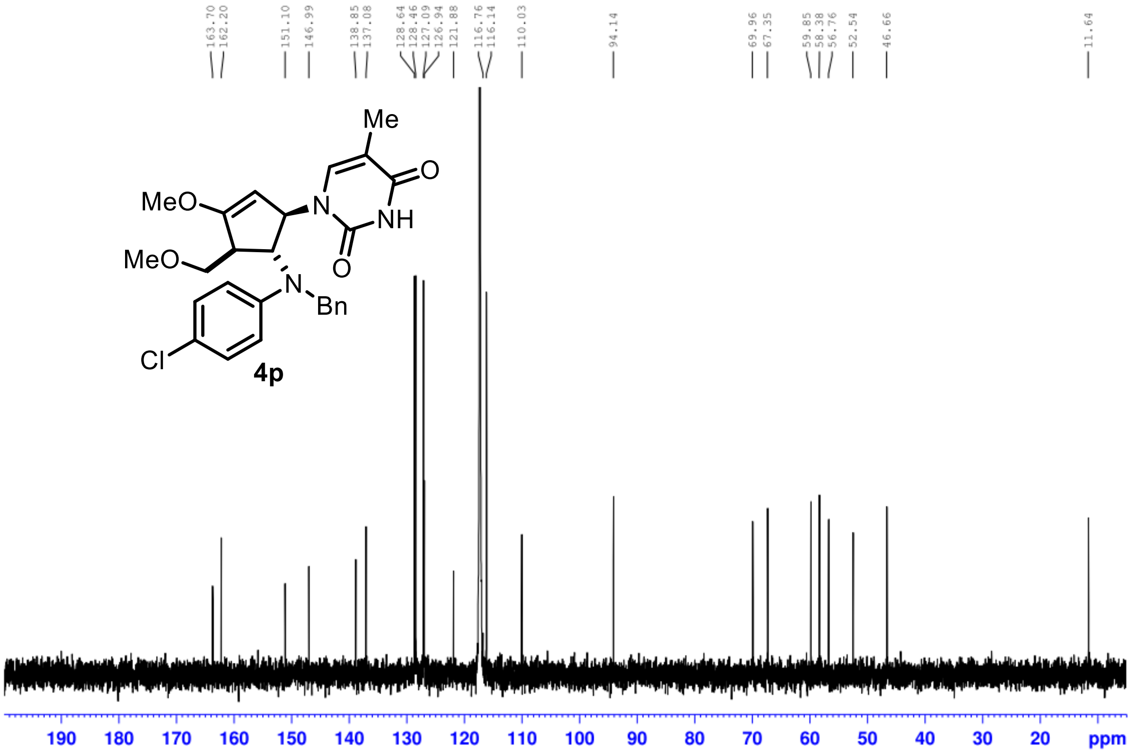

R6-137-p, 1H, BBFO1400, CD3CN, Jun-14

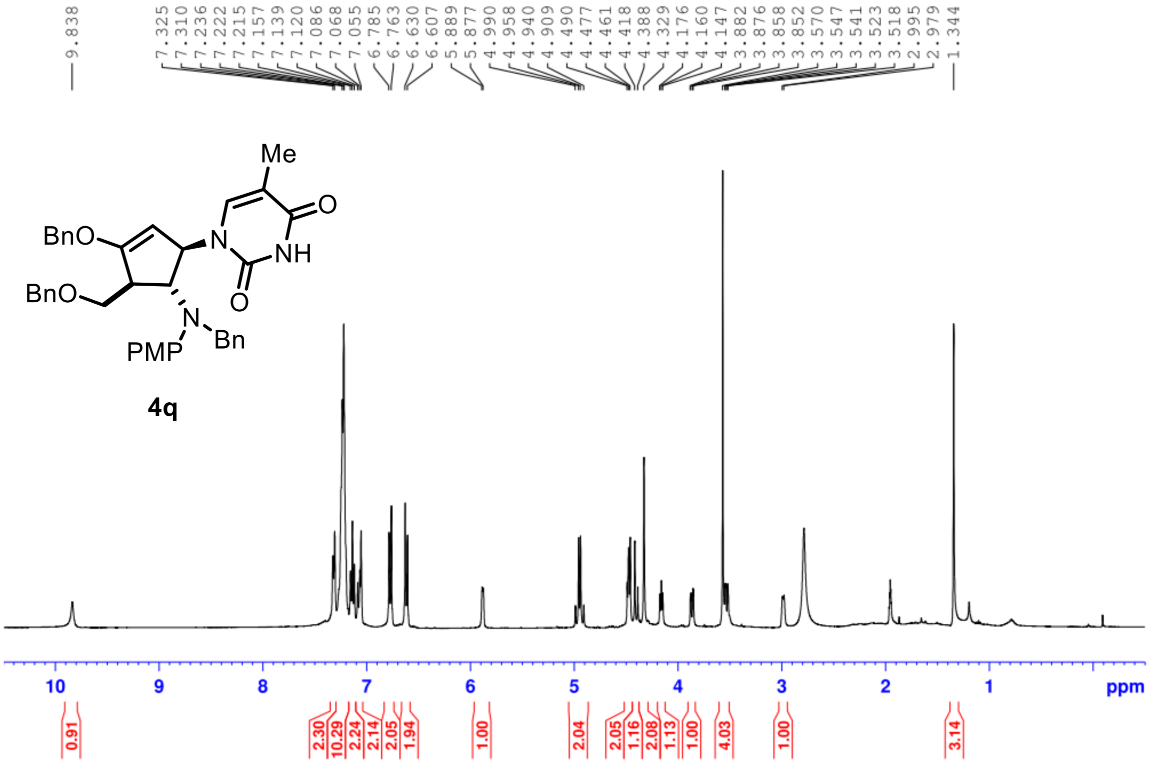

R6-137-p, 13C, BBFO1400, CD3CN, Jun-14

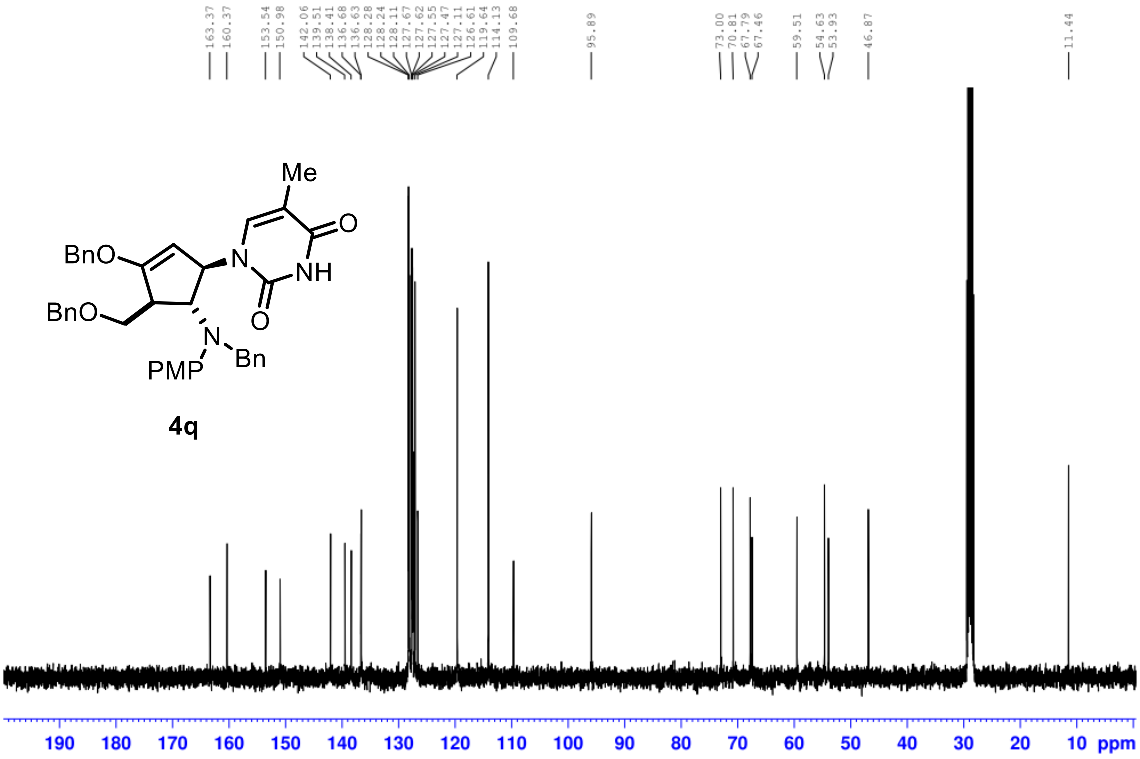

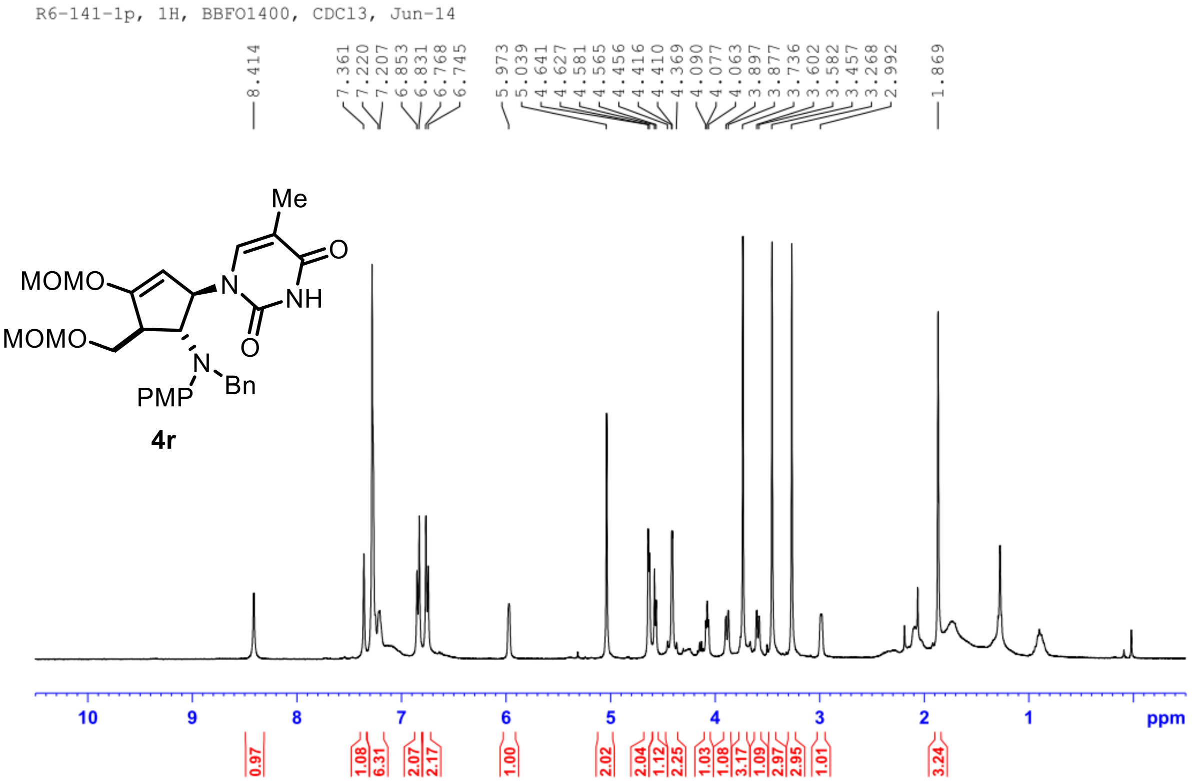

R6-141-1p, 1H, BBFO1400, CDCl3, Jun-14
8.414
7.361
7.220
7.207
6.853
6.831
6.768
6.745
5.973
5.039
4.641
4.627
4.581
4.565
4.456
4.416
4.410
4.369
4.090
4.077
4.063
3.897
3.877
3.736
3.602
3.582
3.457
3.268
2.992
1.869
Me
O
MOMO
N
NH
MOMO
O
N
PMP
Bn
4r
10
9
8
7
6
5
4
3
2
1
ppm
0.97
1.08
6.31
2.07
2.17
1.00
2.02
2.04
1.12
2.25
1.03
1.08
3.17
1.09
2.97
2.95
1.01
3.24

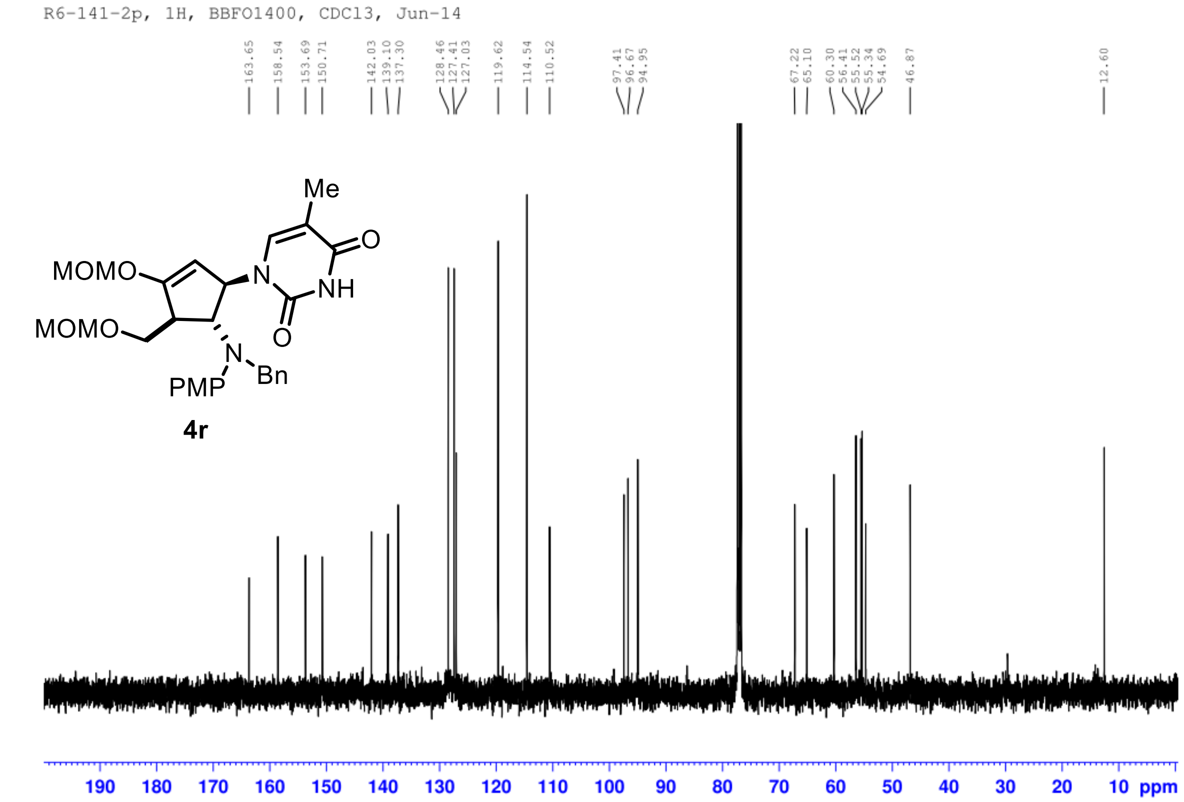

R6-141-2p, 1H, BBFO1400, CDCl3, Jun-14
163.65
158.54
153.69
150.71
142.03
139.10
137.30
128.46
127.41
127.03
119.62
114.54
110.52
97.41
96.67
94.95
67.22
65.10
60.30
56.41
55.52
55.34
54.69
46.87
12.60
Me
O
MOMO
N
NH
MOMO
O
N
PMP
Bn
4r
190
180
170
160
150
140
130
120
110
100
90
80
70
60
50
40
30
20
10
ppm

R6-126-p, 1H, BBFO2 400, CD3CN, Jun-14

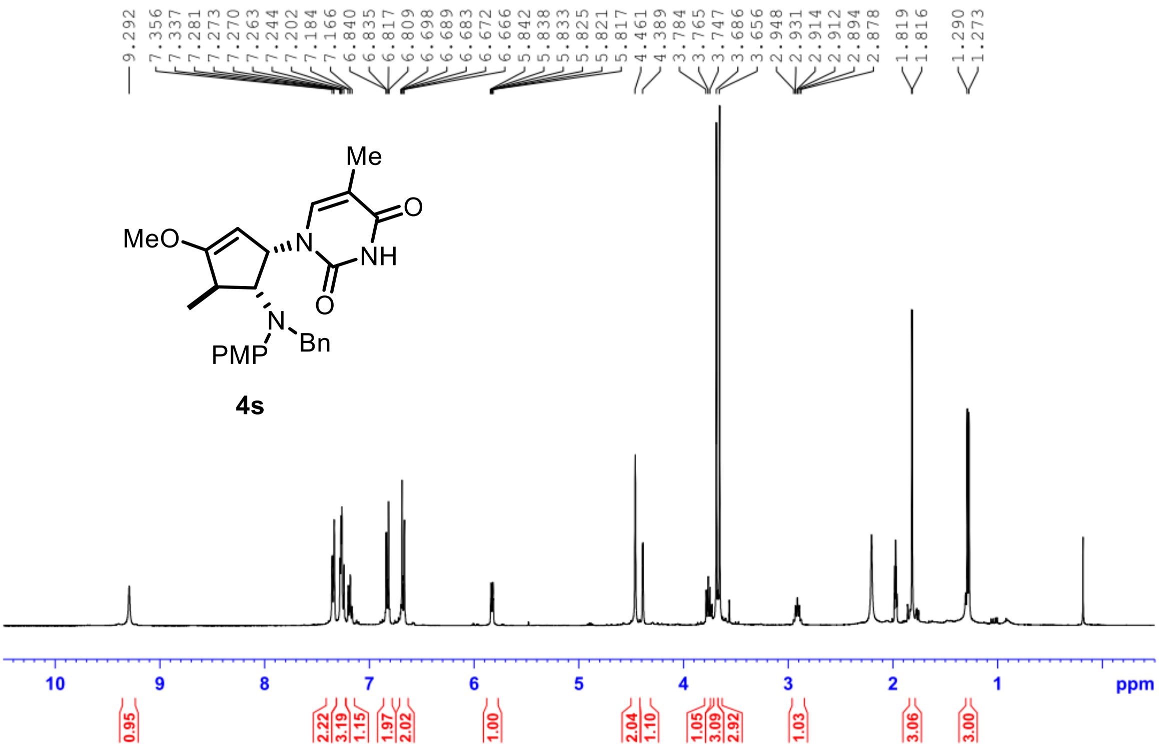

R6-126-p, 13C, BBFO2 400, CD3CN, Jun-14

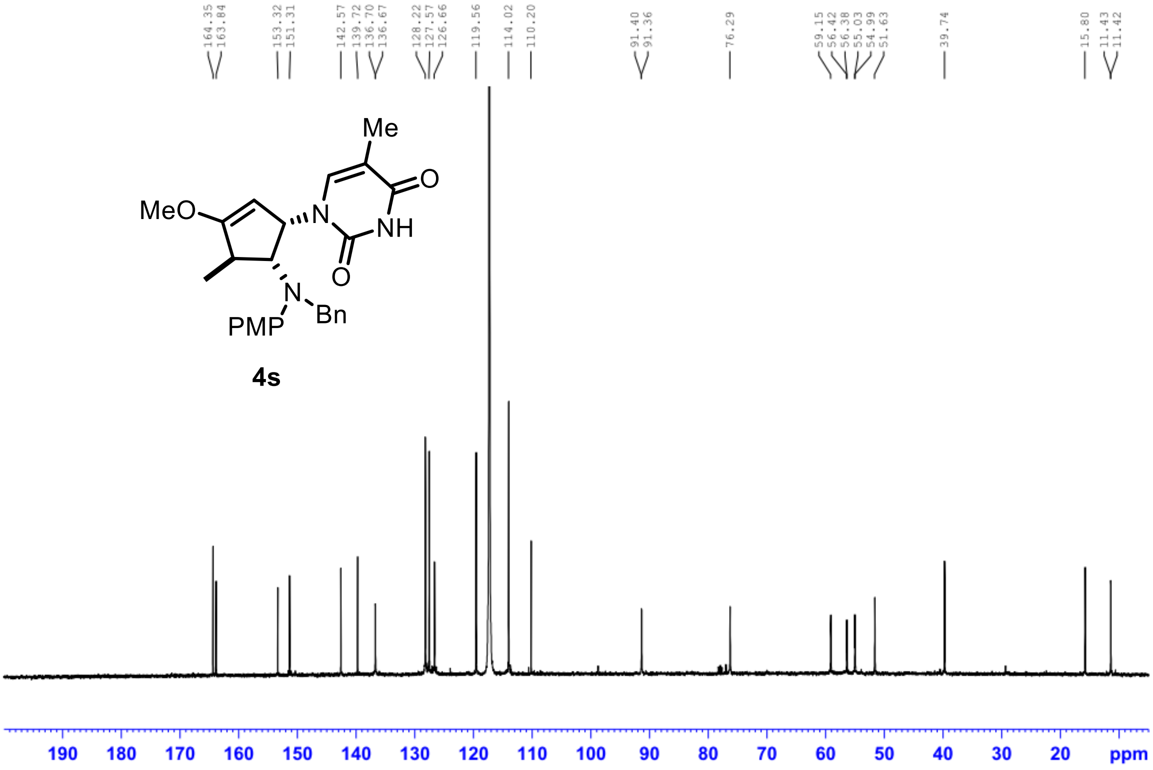

R6-052-2, 1H, BBFO1400, MeOD, Nov-2013

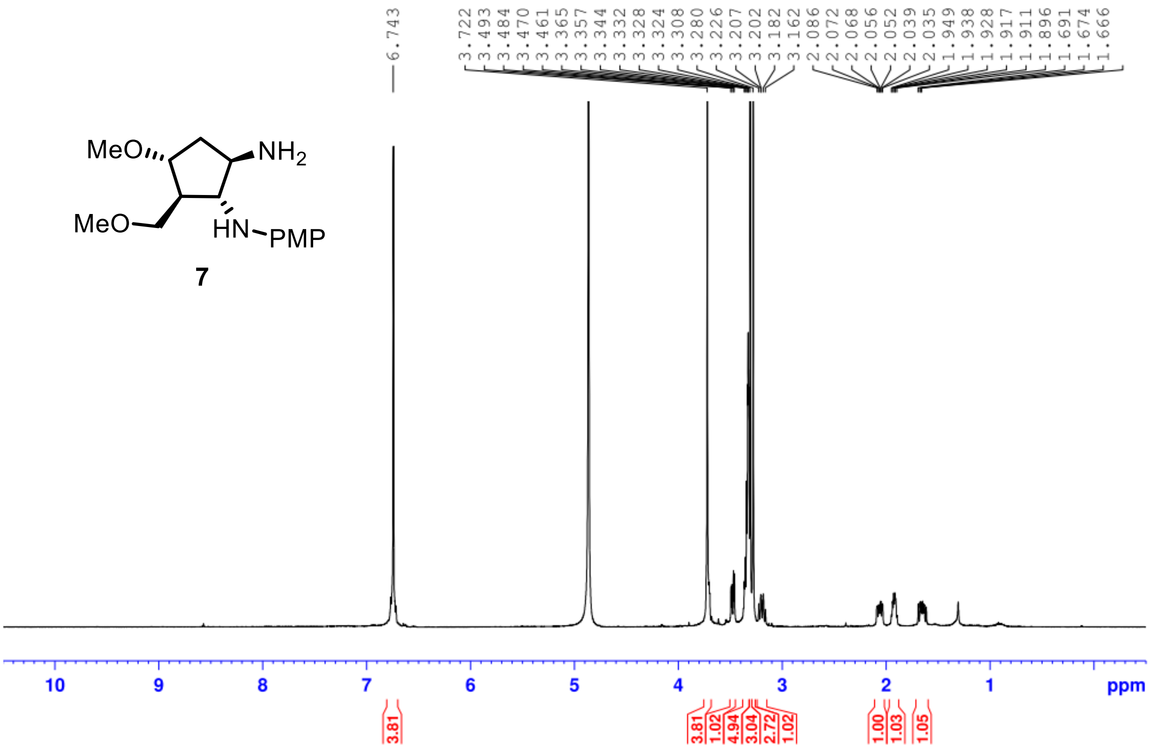

R6-052-2, 13C, BBFO1400, MeOD, Nov-2013

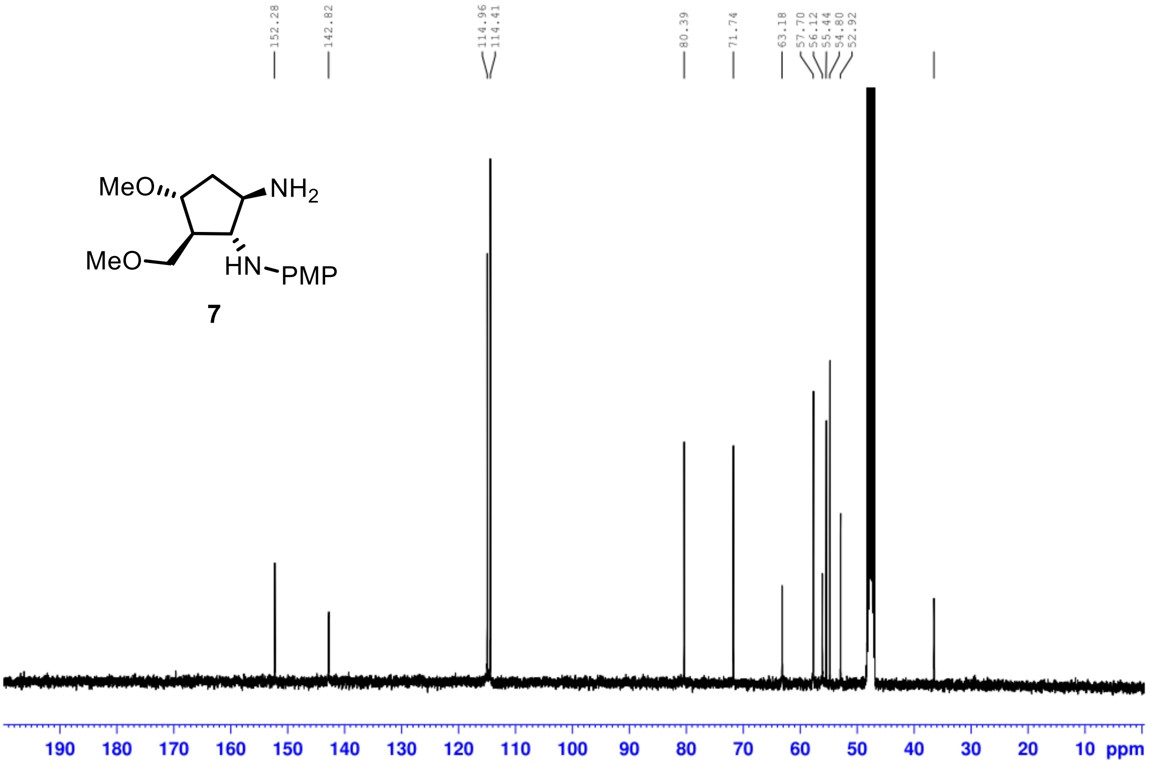

COSY of **7**

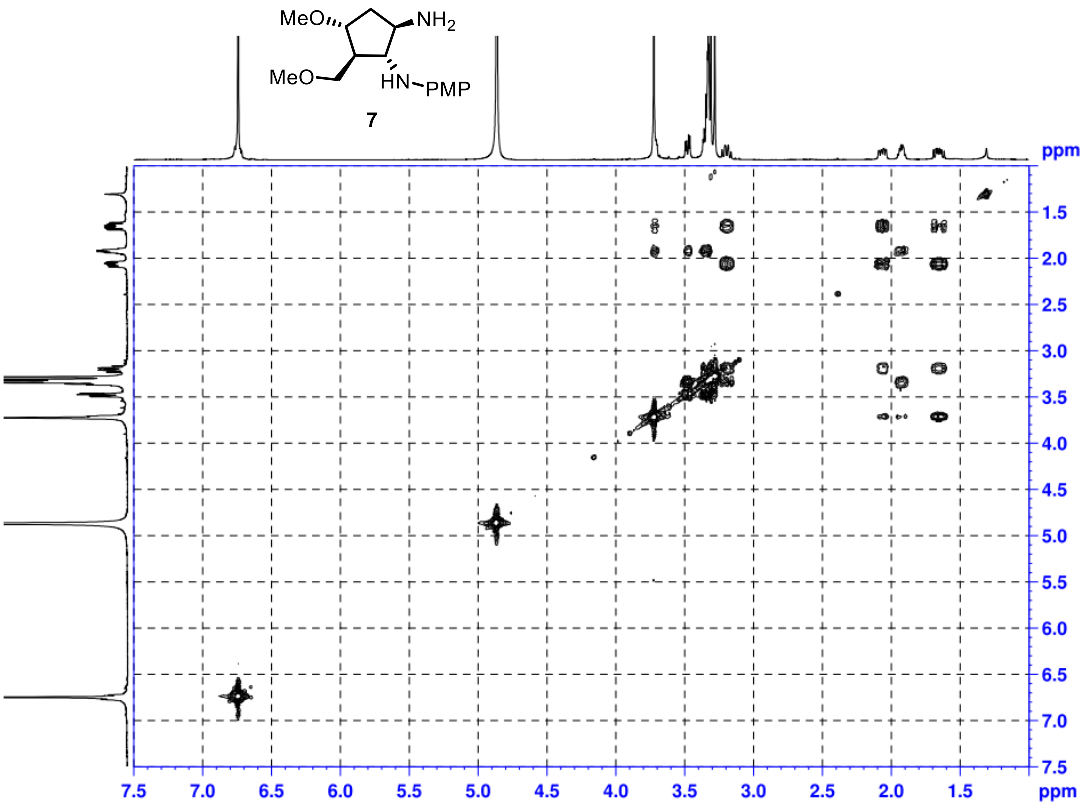
MeO
NH2
MeO
HN
PMP
7
ppm
1.5
2.0
2.5
3.0
3.5
4.0
4.5
5.0
5.5
6.0
6.5
7.0
7.5
7.5
7.0
6.5
6.0
5.5
5.0
4.5
4.0
3.5
3.0
2.5
2.0
1.5
ppm

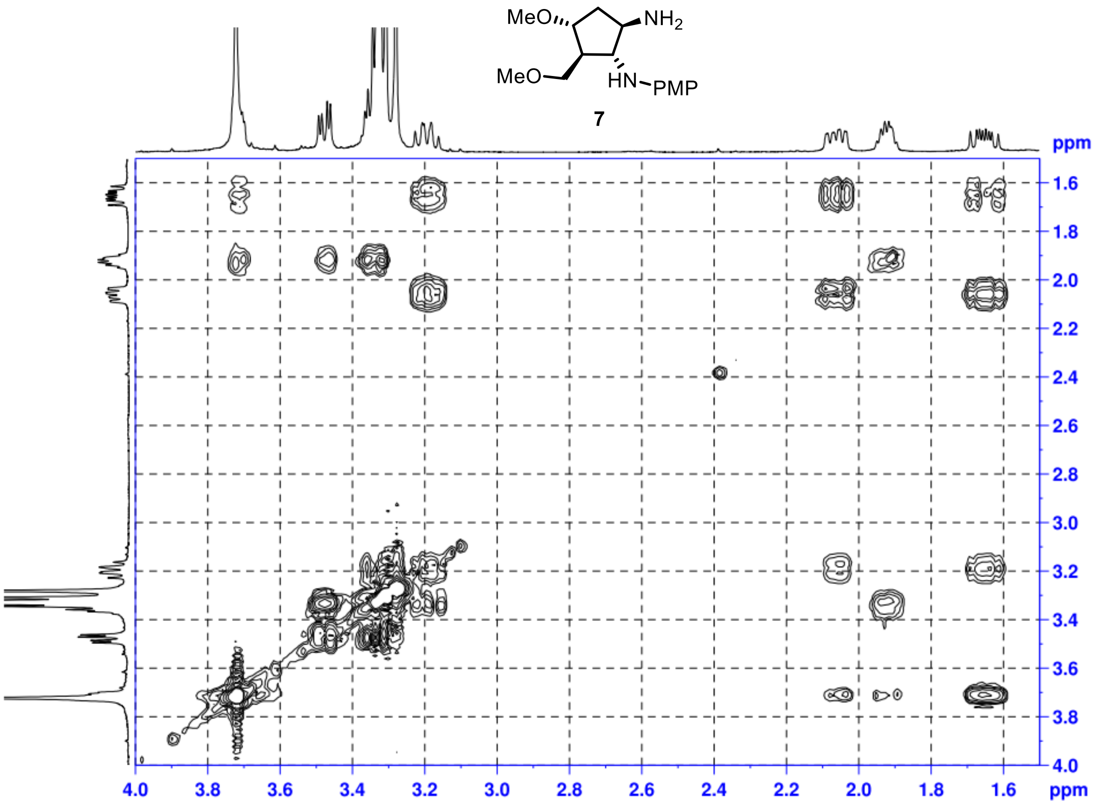
MeO
NH2
MeO
HN
PMP
7
ppm
1.6
1.8
2.0
2.2
2.4
2.6
2.8
3.0
3.2
3.4
3.6
3.8
4.0
4.0
3.8
3.6
3.4
3.2
3.0
2.8
2.6
2.4
2.2
2.0
1.8
1.6
ppm

NOESY of **7**

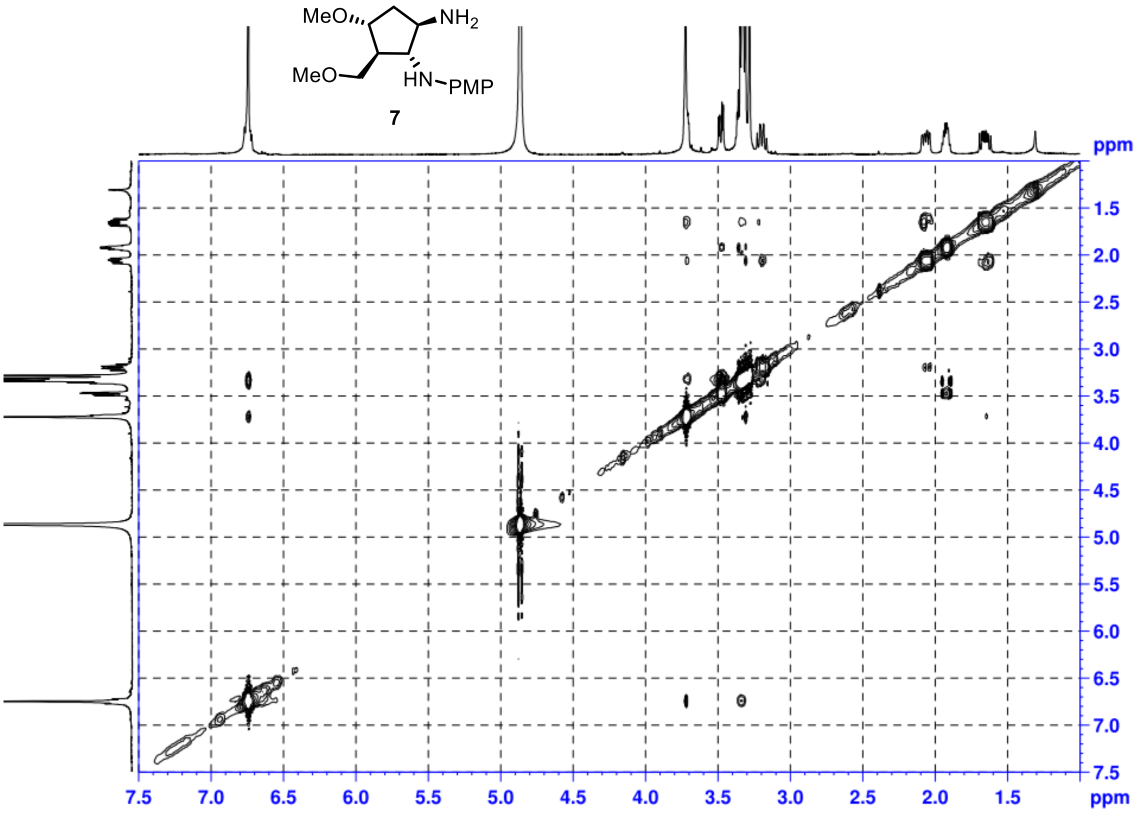
MeO
NH2
MeO
HN
PMP
7
ppm
1.5
2.0
2.5
3.0
3.5
4.0
4.5
5.0
5.5
6.0
6.5
7.0
7.5
7.5
7.0
6.5
6.0
5.5
5.0
4.5
4.0
3.5
3.0
2.5
2.0
1.5
ppm

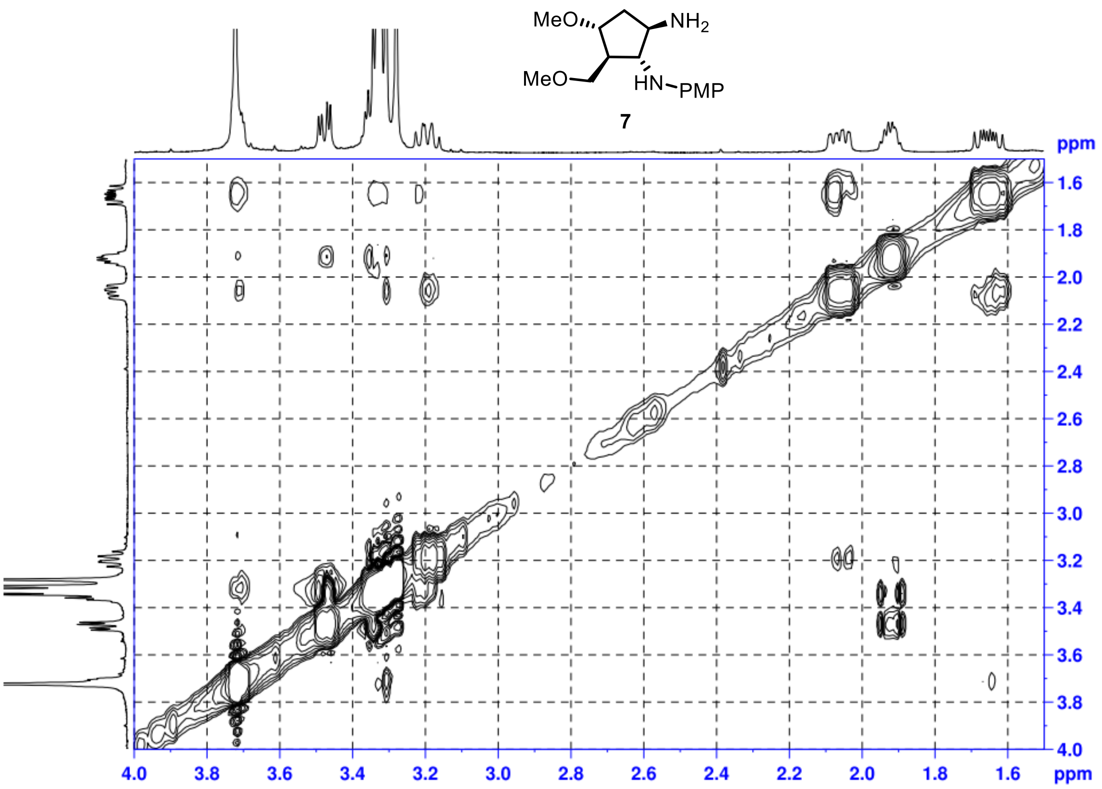
MeO
NH2
MeO
HN
PMP
7
ppm
1.6
1.8
2.0
2.2
2.4
2.6
2.8
3.0
3.2
3.4
3.6
3.8
4.0
4.0
3.8
3.6
3.4
3.2
3.0
2.8
2.6
2.4
2.2
2.0
1.8
1.6
ppm

Computational Section

All DFT calculations were performed with the Gaussian09 program package.[1] Geometry optimizations were done by employing density functional theory B3LYP with 6-311G(d+p) basis set. Frequency analysis was done at the same level of theory to verify that the optimized structures are real minima or saddale point on the potential energy surface, and to get the thermodynamic corrections. Intrinsic reaction coordinate (IRC) calculations were used to confirm that the transition states found connected and related to reactants and products. On the optimized structures, single point energy calculations were performed using 6-311G(d+p). Solvation effects were computed by the polarizable continuum model (PCM) in Gaussian 09. Acetonitrile was used as the solvent to model the reaction medium. Single point energies in solution including all computed corrections were appended with the correction to Gibbs free energy from 6-311G(d+p) frequencies computed for 1 mol/L solution at 298.15 K. to describe reaction energetics, as reflected by the relative Gibbs free energy in Figure S1.

Free energy profile for addition of silylated pyrimidine to oxyallylcation

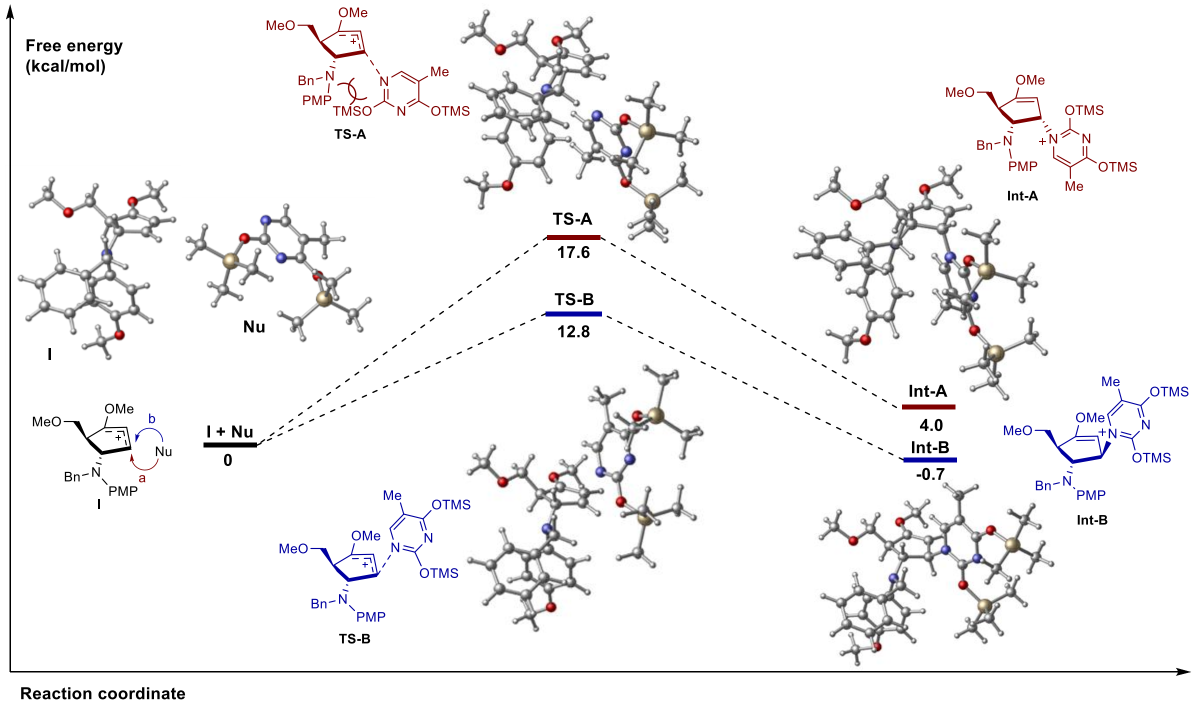

Figure S1. Free energy profile for addition of silylated pyrimidine to oxyallylcation. Energies are calculated at the B3LYP/6-311G(d,p) level of theory with acetonitrile as solvent. Nu= silylated pyrimidine.

Silylated pyrimidine could in principle approach the oxyallyl cation at C-3 from the top or bottom face. To gain more insight into this intriguing preference for formation of the observed diastereomer, a simple theoretical DFT calculation was conducted, the result of which is summarized in the energy diagram depicted in Figure S1. Attack from the bottom face (path a) would lead to pathway with higher activation energy barrier via **TS-A** and result in less energetically favourable **Int-A**, presumably due to steric reason. In stark contrast, alternative pathway in which incoming nucleophile approaches from top face (path b) proceeds through lower energy **TS-B** ($\Delta\Delta G^{\ddagger}$= 4.8 kcal/mol) and renders more stable **Int-B**.

Relevant optimized structures in Figure S1

Oxyallyl cation

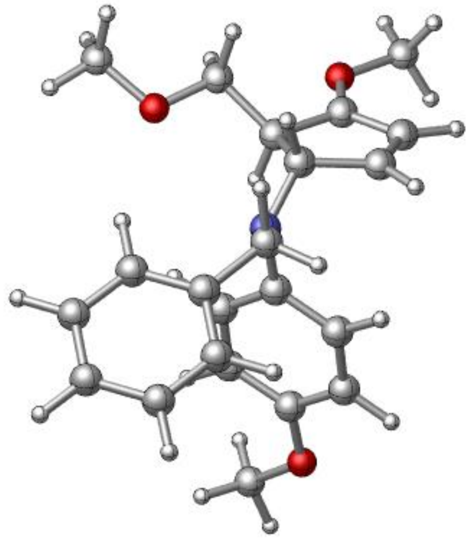

Center Number	Atomic Number	Forces (Hartrees/Bohr) X	Y	Z
1	6	-0.000009831	-0.000006170	-0.000001538
2	6	0.000011593	0.000021835	-0.000010901
3	1	0.000002852	0.000006081	0.000004071
4	1	-0.000000572	-0.000005112	0.000001524
5	6	-0.000005572	0.000020422	0.000001114
6	1	-0.000007684	0.000000381	-0.000003285
7	6	0.000009935	-0.000009087	0.000000802
8	8	-0.000013668	0.000001387	0.000001910
9	6	0.000005025	0.000010806	-0.000004179

10	1	-0.000007218	0.000006588	0.000004926
11	1	-0.000003286	0.000007098	0.000006150
12	1	-0.000004864	0.000004820	0.000001899
13	7	0.000001515	-0.000013086	0.000013248
14	6	-0.000006225	-0.000002509	-0.000011889
15	1	0.000005379	-0.000002185	0.000000548
16	1	0.000000441	0.000002155	-0.000002908
17	6	-0.000005764	-0.000000909	-0.000005559
18	6	-0.000002131	-0.000004535	-0.000003000
19	6	0.000000017	-0.000000195	0.000002976
20	6	0.000001489	0.000002860	-0.000002661
21	1	-0.000001322	0.000003209	-0.000003200
22	6	0.000000404	-0.000000932	-0.000001367
23	1	0.000000344	-0.000002001	0.000000211
24	6	-0.000004712	0.000004924	-0.000005668
25	1	-0.000002782	0.000003629	-0.000005233
26	1	-0.000002917	-0.000000132	0.000000344
27	6	-0.000000273	0.000002164	-0.000002640
28	6	0.000007024	-0.000005777	-0.000003623
29	6	0.000002823	-0.000006527	0.000000487
30	6	0.000003971	-0.000010188	0.000001109
31	1	0.000002473	-0.000003146	0.000000182
32	6	0.000005464	-0.000006151	-0.000008501
33	1	0.000001332	-0.000003485	-0.000005884
34	6	0.000001909	-0.000001880	-0.000000886
35	1	0.000004406	-0.000005509	0.000000008
36	1	0.000004438	-0.000004715	-0.000005668
37	1	0.000005118	-0.000006204	-0.000002473
38	8	-0.000001075	-0.000000224	0.000006715
39	6	-0.000001616	0.000007696	-0.000002549
40	1	-0.000001685	0.000003832	-0.000001614
41	1	-0.000000819	0.000000773	0.000001110
42	1	-0.000001895	0.000003053	0.000002499
43	6	0.000001082	-0.000000185	0.000002021
44	1	-0.000000193	0.000000556	0.000003492
45	1	-0.000000192	0.000003006	0.000004787
46	8	0.000001492	-0.000002437	0.000008705
47	6	0.000000096	-0.000003224	0.000004952
48	1	0.000001935	-0.000001656	0.000006430
49	1	0.000002225	-0.000002740	0.000006196
50	1	0.000002210	-0.000002696	0.000005764
51	6	-0.000001644	-0.000006377	0.000001460
52	1	0.000000945	0.000002699	-0.000000412

Silylated Pyrimidine

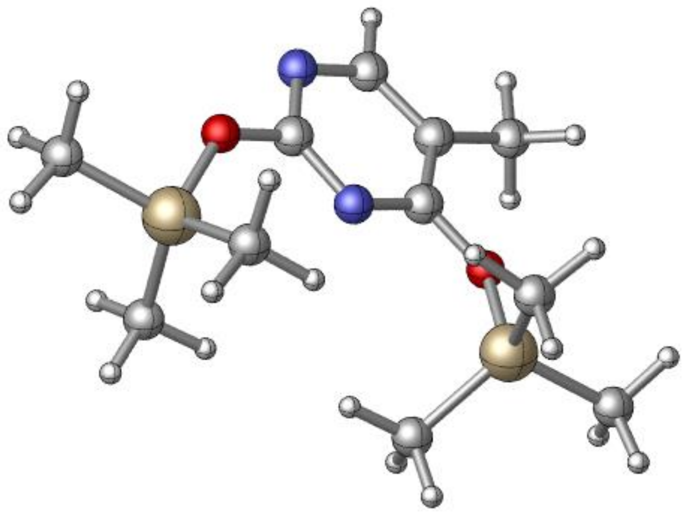

Center Number	Atomic Number	Atomic Type	Coordinates (Angstroms) X	Y	Z
1	6	0	-1.732469	-0.683540	-3.088004
2	6	0	-1.756431	-1.586909	-4.131948
3	1	0	-2.476143	0.106962	-3.033721
4	7	0	-0.827685	-0.710942	-2.098595
5	6	0	0.094055	-1.668467	-2.162291
6	7	0	0.190807	-2.591738	-3.124420
7	6	0	-0.723544	-2.552534	-4.095196
8	8	0	-0.680315	-3.445510	-5.078113
9	14	0	0.521708	-4.580987	-5.578102
10	6	0	0.778632	-5.884269	-4.259827
11	1	0	1.252281	-5.472979	-3.367645
12	1	0	-0.173083	-6.337348	-3.968285
13	1	0	1.421146	-6.679091	-4.652110
14	6	0	2.084412	-3.636776	-5.996146
15	1	0	2.861885	-4.330585	-6.330599
16	1	0	1.901486	-2.922594	-6.803880
17	1	0	2.468508	-3.088062	-5.134025
18	6	0	-0.264799	-5.319902	-7.106648
19	1	0	-1.204972	-5.822360	-6.863054
20	1	0	-0.471360	-4.551227	-7.856174
21	1	0	0.403858	-6.059542	-7.557440
22	8	0	0.982806	-1.668078	-1.165541
23	14	0	2.164642	-2.830750	-0.686235
24	6	0	3.479805	-3.005873	-2.006505
25	1	0	3.906303	-2.032222	-2.264243
26	1	0	4.291790	-3.638505	-1.634002
27	1	0	3.083545	-3.460033	-2.915271
28	6	0	2.875407	-2.045397	0.857065
29	1	0	3.320012	-1.071223	0.635218
30	1	0	2.108145	-1.905443	1.623076
31	1	0	3.658878	-2.682778	1.278296
32	6	0	1.288690	-4.439741	-0.295841

33	1	0	0.767934	-4.839614	-1.167967
34	1	0	2.011790	-5.188797	0.041219
35	1	0	0.556949	-4.296244	0.504160
36	6	0	-2.779502	-1.555557	-5.229960
37	1	0	-3.358100	-2.483083	-5.258263
38	1	0	-3.472231	-0.724475	-5.086393
39	1	0	-2.305899	-1.443873	-6.209136

TS-A

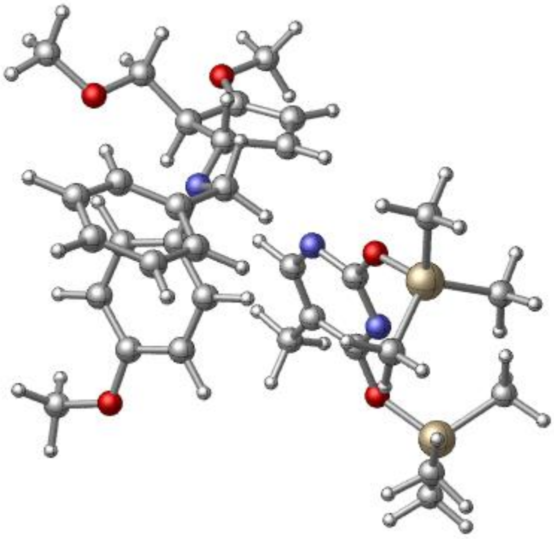

Center Number	Atomic Number	Atomic Type	Coordinates (Angstroms) X	Y	Z
1	6	0	-2.420938	2.561217	-0.225930
2	6	0	-1.438386	1.549099	0.423223
3	1	0	-3.263979	2.028241	-0.674859
4	1	0	-0.905955	2.114379	1.203720
5	6	0	-0.416333	2.415662	-1.561829
6	1	0	0.316208	2.620536	-2.327454
7	6	0	-1.597979	3.121868	-1.350529
8	8	0	-2.036951	4.174964	-1.966666
9	6	0	-1.257410	4.750264	-3.054732
10	1	0	-1.152530	4.017129	-3.853583
11	1	0	-1.833317	5.605367	-3.392799
12	1	0	-0.285158	5.065553	-2.677720
13	7	0	-1.986300	0.387983	1.105275
14	6	0	-1.057460	-0.252529	2.042650
15	1	0	-0.376005	0.528573	2.395594
16	1	0	-0.420223	-1.009467	1.566191
17	6	0	-2.968492	-0.455425	0.488983
18	6	0	-2.678330	-1.768612	0.088322
19	6	0	-4.289876	-0.015337	0.364980
20	6	0	-3.660878	-2.591992	-0.441134
21	1	0	-1.675162	-2.158937	0.194536
22	6	0	-5.280892	-0.823588	-0.195698

23	1	0	-4.554469	0.966594	0.736480
24	6	0	-4.970224	-2.122940	-0.604033
25	1	0	-3.430516	-3.606768	-0.743758
26	1	0	-6.287886	-0.437550	-0.277974
27	6	0	-1.749481	-0.859607	3.249904
28	6	0	-2.751972	-0.153669	3.924560
29	6	0	-1.357344	-2.107181	3.739862
30	6	0	-3.344472	-0.684493	5.067412
31	1	0	-3.067582	0.809232	3.538689
32	6	0	-1.946881	-2.640255	4.887068
33	1	0	-0.586160	-2.667606	3.221100
34	6	0	-2.942609	-1.929967	5.553907
35	1	0	-4.120349	-0.126927	5.580652
36	1	0	-1.632475	-3.611160	5.253676
37	1	0	-3.405535	-2.343283	6.442964
38	8	0	-5.858922	-3.003006	-1.149450
39	6	0	-7.213454	-2.584640	-1.313555
40	1	0	-7.732837	-3.429721	-1.761216
41	1	0	-7.675556	-2.343412	-0.351195
42	1	0	-7.285812	-1.719693	-1.980418
43	6	0	-2.974939	3.660165	0.688139
44	1	0	-2.149319	4.184831	1.191353
45	1	0	-3.515458	4.394136	0.077385
46	8	0	-3.843208	3.074579	1.638056
47	6	0	-4.441843	4.042021	2.492529
48	1	0	-5.037417	4.762568	1.918207
49	1	0	-5.093614	3.502535	3.178657
50	1	0	-3.683242	4.586970	3.067847
51	6	0	-1.607418	-0.426728	-3.095751
52	6	0	-1.707846	-1.371491	-4.096092
53	1	0	-2.277329	0.428690	-3.082789
54	7	0	-0.709106	-0.490182	-2.102861
55	6	0	0.138497	-1.517299	-2.121247
56	7	0	0.152501	-2.487932	-3.039536
57	6	0	-0.761917	-2.420551	-4.009323
58	8	0	-0.803463	-3.361617	-4.943989
59	14	0	0.265943	-4.650321	-5.371258
60	6	0	0.417035	-5.869900	-3.960114
61	1	0	0.968770	-5.451655	-3.117513
62	1	0	-0.569459	-6.182870	-3.606707
63	1	0	0.947075	-6.763194	-4.305820
64	6	0	1.904875	-3.901638	-5.881897
65	1	0	2.602441	-4.692643	-6.174126
66	1	0	1.775575	-3.234632	-6.738859
67	1	0	2.360771	-3.331132	-5.070255
68	6	0	-0.622774	-5.417201	-6.827823
69	1	0	-1.604012	-5.800665	-6.534974
70	1	0	-0.764547	-4.691254	-7.632884
71	1	0	-0.040889	-6.253590	-7.226921
72	8	0	1.029080	-1.536362	-1.129956
73	14	0	2.187936	-2.719353	-0.637862
74	6	0	3.431174	-3.022437	-2.003555
75	1	0	3.860143	-2.080699	-2.357457
76	1	0	4.250224	-3.640003	-1.621217
77	1	0	2.984205	-3.538433	-2.853805
78	6	0	2.997898	-1.875286	0.822585
79	1	0	3.485696	-0.944613	0.519755
80	1	0	2.269407	-1.641542	1.603300
81	1	0	3.761083	-2.526782	1.259095
82	6	0	1.269567	-4.266239	-0.118417
83	1	0	0.691722	-4.688860	-0.942591
84	1	0	1.979030	-5.026341	0.223116
85	1	0	0.583809	-4.052609	0.706187
86	6	0	-2.723619	-1.304670	-5.199100
87	1	0	-3.372928	-2.184442	-5.191612
88	1	0	-3.348716	-0.416487	-5.092989
89	1	0	-2.240882	-1.271203	-6.179597
90	6	0	-0.346738	1.395236	-0.633763

91	1	0	0.517190	0.769424	-0.474053

Int-A

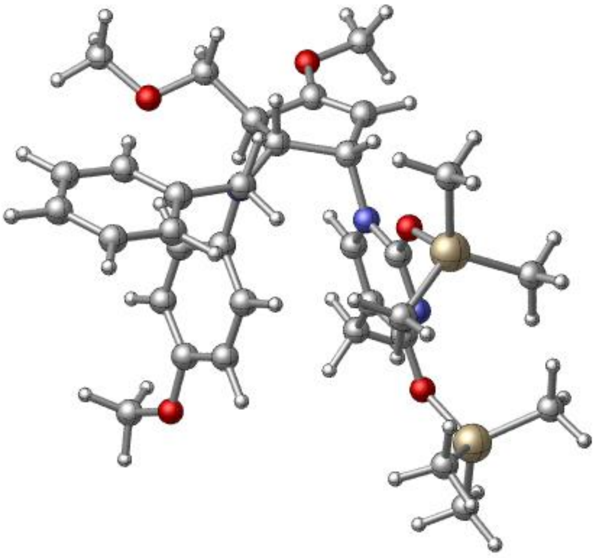

Center Number	Atomic Number	Forces (Hartrees/Bohr) X	Y	Z
1	6	-0.001153162	-0.001139121	0.001344147
2	6	0.006028966	0.007573901	0.000098438
3	1	-0.002123940	0.001498354	0.000391412
4	1	0.001085070	-0.000386511	-0.003052399
5	6	-0.008421827	-0.003660993	-0.004066102
6	1	0.001635493	0.000828768	0.001940898
7	6	0.001071011	-0.002923463	-0.002227148
8	8	-0.001072179	0.002818307	0.002512474
9	6	0.001530951	0.000659771	-0.001318951
10	1	0.000217669	0.000100247	0.000306618
11	1	-0.000036133	0.000066132	-0.000022678
12	1	-0.000505156	-0.000362607	-0.000180392
13	7	-0.003534586	-0.004374680	-0.000963584
14	6	0.002049598	-0.001100568	-0.001052037
15	1	-0.000384647	-0.001032297	-0.001060757
16	1	-0.002403617	0.003584556	0.002517826
17	6	0.000546110	-0.004451155	0.006195639
18	6	-0.021903702	-0.022999832	0.039767759
19	6	-0.004282505	-0.000592856	-0.001395540
20	6	-0.025238690	-0.021518763	0.038951889
21	1	-0.025289840	-0.013164409	0.024521002
22	6	-0.002201778	0.003979549	0.002168594
23	1	0.000015141	0.000039583	-0.000413857
24	6	0.007330479	-0.004822708	0.006624964
25	1	-0.003684050	-0.004255467	0.017090088
26	1	0.000024911	0.000362253	-0.000370566
27	6	-0.000030085	0.001947995	-0.000975386
28	6	-0.000349615	0.000364355	0.000140024
29	6	0.000347876	-0.001406435	-0.000700401
30	6	-0.000760440	0.000759735	0.000568280

31	1	0.000113086	-0.000068968	0.000095971
32	6	-0.000281836	-0.000730000	0.000462101
33	1	-0.002483065	0.000253137	0.001633027
34	6	-0.000506051	-0.000834592	0.000871234
35	1	-0.000105666	0.000150498	0.000100222
36	1	0.000140157	-0.000163109	-0.000005238
37	1	-0.000129817	-0.000063459	0.000222732
38	8	0.001279738	-0.001419786	-0.001447114
39	6	-0.003896827	0.000988402	-0.000317549
40	1	-0.000146281	0.000071010	0.000040894
41	1	0.000518539	0.000125790	-0.000571528
42	1	0.000231126	-0.000666171	0.000240734
43	6	0.003717694	0.001390317	-0.001518215
44	1	-0.000765406	-0.000992890	-0.000653900
45	1	-0.000433318	-0.000253885	0.000245601
46	8	-0.002324644	-0.001316457	0.001482041
47	6	0.000408149	0.000365469	0.000412799
48	1	0.000104668	0.000128019	-0.000076245
49	1	-0.000137607	-0.000084351	0.000284769
50	1	-0.000215340	-0.000040769	-0.000305988
51	6	0.016577150	0.020057029	-0.017995797
52	6	0.026014607	0.007520421	-0.040532985
53	1	0.001926972	-0.000906006	-0.004318121
54	7	0.008787993	0.014105742	-0.020045890
55	6	0.008178138	0.003305314	-0.004629911
56	7	0.004447446	0.000524473	-0.000860844
57	6	0.003754844	0.013629839	-0.014483588
58	8	-0.000872635	-0.007195511	-0.002940214
59	14	0.000103067	0.002390416	0.001754677
60	6	-0.000010613	-0.001012301	0.000475554
61	1	-0.000160621	0.000209516	-0.000354777
62	1	-0.000273214	0.000552923	-0.000217980
63	1	0.000270938	0.000156741	-0.000622602
64	6	0.001102231	-0.000171509	-0.001012017
65	1	-0.000326644	-0.000438347	0.000402442
66	1	-0.000669961	0.000246327	0.000233876
67	1	-0.000419882	0.000069061	0.000377443
68	6	-0.001206767	-0.000944801	-0.001283851
69	1	0.000074329	0.000252912	0.000577107
70	1	0.000235862	0.000542000	0.000081017
71	1	0.000575791	0.000110439	0.000358602
72	8	0.001411782	0.000067018	0.001731554
73	14	0.000016406	0.000709319	-0.001508779
74	6	0.000725953	-0.000334289	-0.000525556
75	1	-0.000481592	0.000547195	0.000209955
76	1	-0.000313388	-0.000063976	0.000571367
77	1	-0.000186774	0.000059765	0.000112946
78	6	0.000989509	0.000941855	0.001171775
79	1	-0.000542284	0.000277700	-0.000776587
80	1	-0.000593080	-0.000107326	-0.000255708
81	1	-0.000244145	-0.000583676	-0.000283027
82	6	0.000875533	-0.001185925	0.001099116
83	1	-0.000323754	0.000287201	-0.000237072
84	1	0.000769209	0.000214934	-0.000581147
85	1	0.003832124	-0.001117711	-0.001888458
86	6	0.002665365	0.004778477	-0.015299072
87	1	0.000447898	0.008353543	-0.013861957
88	1	0.000380523	-0.000218550	-0.000022886
89	1	0.002433898	0.001880490	-0.001084445
90	6	-0.002554219	0.004506561	-0.003664355
91	1	0.008987383	-0.005247135	0.011593592

TS-B

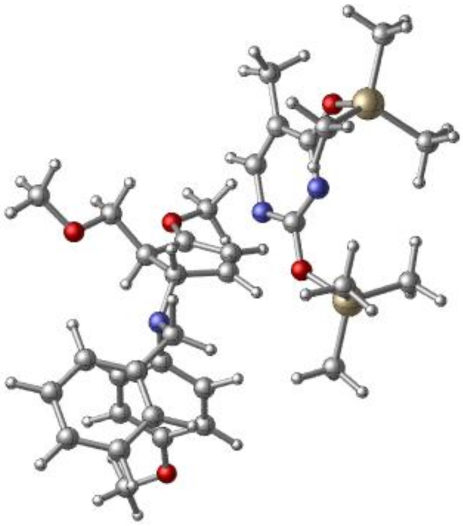

Center Number	Atomic Number	Atomic Type	Coordinates (Angstroms) X	Y	Z
1	6	0	-2.279398	2.270690	0.138660
2	6	0	-1.484623	0.935048	0.197203
3	1	0	-3.349237	2.048594	0.088403
4	1	0	-0.733634	1.004587	0.985248
5	6	0	-1.050954	2.009152	-1.917620
6	1	0	-0.695239	2.185281	-2.921340
7	6	0	-1.881450	2.851113	-1.185988
8	8	0	-2.361929	4.012566	-1.513105
9	6	0	-1.993893	4.611629	-2.788295
10	1	0	-2.355422	3.984610	-3.602602
11	1	0	-2.489986	5.576741	-2.800877
12	1	0	-0.912358	4.734641	-2.835876
13	7	0	-2.308498	-0.233499	0.509731
14	6	0	-1.578254	-1.383232	1.067376
15	1	0	-0.757476	-0.977034	1.666367
16	1	0	-1.116108	-2.011242	0.294031
17	6	0	-3.372726	-0.562491	-0.407543
18	6	0	-3.158388	-1.287811	-1.590375
19	6	0	-4.678286	-0.177795	-0.101222
20	6	0	-4.210360	-1.596208	-2.439107
21	1	0	-2.163037	-1.630216	-1.848413
22	6	0	-5.746235	-0.472096	-0.950426
23	1	0	-4.863480	0.353422	0.824894
24	6	0	-5.514090	-1.184369	-2.129929
25	1	0	-4.045614	-2.157520	-3.351091
26	1	0	-6.741973	-0.152011	-0.676445
27	6	0	-2.454320	-2.247632	1.953823
28	6	0	-3.126157	-1.685174	3.045861
29	6	0	-2.582680	-3.617589	1.718530
30	6	0	-3.904327	-2.477615	3.884704
31	1	0	-3.038352	-0.619920	3.228612
32	6	0	-3.361073	-4.416343	2.558108
33	1	0	-2.073331	-4.065005	0.871490
34	6	0	-4.024006	-3.848225	3.643208
35	1	0	-4.417481	-2.028923	4.728273

36	1	0	-3.451680	-5.478693	2.360434
37	1	0	-4.631044	-4.465445	4.296129
38	8	0	-6.474794	-1.529204	-3.030229
39	6	0	-7.825784	-1.144570	-2.767454
40	1	0	-8.407142	-1.514697	-3.609502
41	1	0	-8.195223	-1.598271	-1.842905
42	1	0	-7.924465	-0.056469	-2.707681
43	6	0	-2.063304	3.228715	1.312644
44	1	0	-0.994445	3.457790	1.433339
45	1	0	-2.589803	4.172326	1.118701
46	8	0	-2.569546	2.604860	2.476927
47	6	0	-2.391993	3.401425	3.641614
48	1	0	-2.914089	4.362258	3.549471
49	1	0	-2.812556	2.844185	4.478076
50	1	0	-1.328767	3.593073	3.834361
51	6	0	-0.763896	0.892650	-1.146653
52	1	0	-0.350252	-0.022411	-1.538573
53	6	0	1.954305	2.372116	-0.323762
54	6	0	3.278407	2.611380	-0.021647
55	1	0	1.248101	3.195799	-0.384810
56	7	0	1.459168	1.150477	-0.561655
57	6	0	2.311996	0.127841	-0.526812
58	7	0	3.611331	0.229528	-0.233525
59	6	0	4.087805	1.449776	0.024738
60	8	0	5.367977	1.603176	0.333646
61	14	0	6.611725	0.465587	0.732542
62	6	0	6.974312	-0.632174	-0.738662
63	1	0	6.142691	-1.301631	-0.962178
64	1	0	7.184446	-0.033248	-1.629324
65	1	0	7.858449	-1.243118	-0.530373
66	6	0	6.070552	-0.482796	2.253738
67	1	0	6.854166	-1.186222	2.551892
68	1	0	5.894345	0.197647	3.091539
69	1	0	5.155574	-1.051097	2.074936
70	6	0	8.056257	1.595035	1.100754
71	1	0	8.331701	2.183612	0.221373
72	1	0	7.823656	2.285138	1.916118
73	1	0	8.929276	1.005593	1.397016
74	8	0	1.794943	-1.064389	-0.810468
75	14	0	2.566523	-2.547216	-1.262064
76	6	0	3.542292	-3.234299	0.178924
77	1	0	2.913163	-3.326227	1.068715
78	1	0	3.913574	-4.232746	-0.073108
79	1	0	4.397550	-2.604017	0.424864
80	6	0	1.105780	-3.642473	-1.667828
81	1	0	0.475140	-3.807210	-0.790403
82	1	0	0.489187	-3.207607	-2.458974
83	1	0	1.457669	-4.618978	-2.014402
84	6	0	3.613589	-2.218845	-2.780308
85	1	0	4.384140	-1.468952	-2.591252
86	1	0	4.109375	-3.141542	-3.097409
87	1	0	2.991836	-1.870855	-3.610071
88	6	0	3.834938	3.978680	0.249341
89	1	0	4.632402	4.226414	-0.456514
90	1	0	3.054285	4.736485	0.165530
91	1	0	4.265546	4.036844	1.252742

Int-B

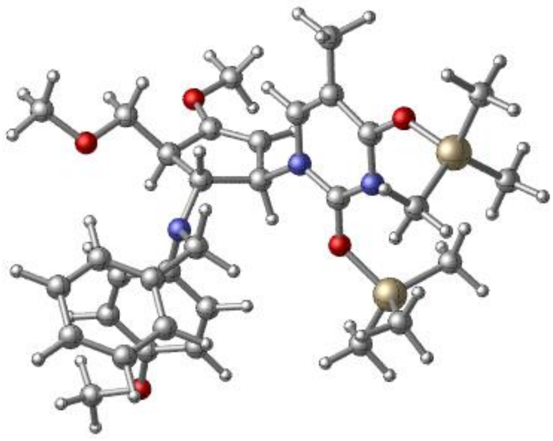

Center Number	Atomic Number	Atomic Type	Coordinates (Angstroms) X	Y	Z
1	6	0	-2.254622	2.491534	-0.308590
2	6	0	-1.240443	1.405611	0.145547
3	1	0	-3.163423	2.008570	-0.679213
4	1	0	-0.665911	1.833865	0.974576
5	6	0	-0.464190	2.503292	-1.896867
6	1	0	0.124413	2.741007	-2.772298
7	6	0	-1.568910	3.147702	-1.479131
8	8	0	-2.164706	4.235532	-1.977512
9	6	0	-1.578008	4.843140	-3.134393
10	1	0	-1.571044	4.142077	-3.973653
11	1	0	-2.203379	5.699719	-3.373224
12	1	0	-0.558358	5.175916	-2.918594
13	7	0	-1.837582	0.193012	0.659060
14	6	0	-0.983925	-0.644530	1.504165
15	1	0	-0.258411	0.024104	1.983768
16	1	0	-0.395588	-1.376678	0.927132
17	6	0	-2.811767	-0.499124	-0.142381
18	6	0	-2.446952	-1.470059	-1.086562
19	6	0	-4.168985	-0.234807	0.040771
20	6	0	-3.404869	-2.140580	-1.830381
21	1	0	-1.401447	-1.716645	-1.229375
22	6	0	-5.143631	-0.894091	-0.708078
23	1	0	-4.457907	0.497507	0.784716
24	6	0	-4.765214	-1.855392	-1.650350
25	1	0	-3.131489	-2.896084	-2.557104
26	1	0	-6.186292	-0.663413	-0.536868
27	6	0	-1.753200	-1.378908	2.586359
28	6	0	-2.667133	-0.688040	3.388564
29	6	0	-1.534319	-2.735755	2.823335
30	6	0	-3.341545	-1.345665	4.412183
31	1	0	-2.852440	0.361691	3.188211
32	6	0	-2.208206	-3.396697	3.849988
33	1	0	-0.837179	-3.285062	2.197718
34	6	0	-3.112923	-2.701954	4.647039

35	1	0	-4.049050	-0.802524	5.028882
36	1	0	-2.032079	-4.452750	4.020497
37	1	0	-3.641604	-3.213361	5.443239
38	8	0	-5.623186	-2.563755	-2.427287
39	6	0	-7.023191	-2.349332	-2.274512
40	1	0	-7.503411	-3.022655	-2.981583
41	1	0	-7.355372	-2.591824	-1.259820
42	1	0	-7.297658	-1.316607	-2.514294
43	6	0	-2.668350	3.490072	0.773454
44	1	0	-1.778513	3.998407	1.184244
45	1	0	-3.306786	4.261743	0.322999
46	8	0	-3.361650	2.808480	1.798454
47	6	0	-3.864303	3.686182	2.794113
48	1	0	-4.563090	4.416612	2.365972
49	1	0	-4.391197	3.074348	3.525488
50	1	0	-3.052677	4.228086	3.298379
51	6	0	-0.233160	1.273111	-1.076786
52	1	0	-0.410680	0.355046	-1.629557
53	6	0	1.767812	2.233053	0.032986
54	6	0	3.042200	2.215526	0.514060
55	1	0	1.129021	3.101960	0.114665
56	7	0	1.204983	1.166094	-0.610345
57	6	0	1.971224	0.053306	-0.804519
58	7	0	3.215546	-0.035026	-0.356097
59	6	0	3.754416	0.995708	0.300236
60	8	0	4.969884	0.920770	0.767372
61	14	0	6.154384	-0.367747	0.911120
62	6	0	6.605829	-0.958490	-0.803717
63	1	0	5.783842	-1.485991	-1.289971
64	1	0	6.907905	-0.124724	-1.443485
65	1	0	7.455553	-1.645937	-0.742613
66	6	0	5.393539	-1.688865	1.992687
67	1	0	6.097786	-2.516409	2.123785
68	1	0	5.159906	-1.298264	2.986838
69	1	0	4.476800	-2.097350	1.562790
70	6	0	7.567182	0.516699	1.745167
71	1	0	7.951010	1.333527	1.128495
72	1	0	7.265809	0.932285	2.710035
73	1	0	8.394044	-0.176561	1.927264
74	8	0	1.416994	-0.928436	-1.459339
75	14	0	2.116955	-2.341418	-2.253835
76	6	0	2.876501	-3.452713	-0.961373
77	1	0	2.162238	-3.688859	-0.167947
78	1	0	3.174356	-4.398993	-1.424403
79	1	0	3.761777	-3.007244	-0.506706
80	6	0	0.618645	-3.112968	-3.049965
81	1	0	-0.088289	-3.491647	-2.307710
82	1	0	0.092244	-2.407365	-3.697494
83	1	0	0.926933	-3.961269	-3.669171
84	6	0	3.335441	-1.686123	-3.510431
85	1	0	4.143558	-1.117667	-3.045857
86	1	0	3.788064	-2.517912	-4.059189
87	1	0	2.839698	-1.043779	-4.243478
88	6	0	3.673142	3.377224	1.223195
89	1	0	4.580338	3.700104	0.707068
90	1	0	2.985885	4.222212	1.279418
91	1	0	3.965495	3.099398	2.238724

Reference

1) Gaussian 09, Revision A.02.

M. J. Frisch, G. W. Trucks, H. B. Schlegel, G. E. Scuseria, M. A. Robb, J. R. Cheeseman, G. Scalmani, V. Barone, B. Mennucci, G. A. Petersson, H. Nakatsuji, M. Caricato, X. Li, H. P. Hratchian, A. F. Izmaylov, J. Bloino, G. Zheng, J. L. Sonnenberg, M. Hada, M. Ehara, K. Toyota, R. Fukuda, J. Hasegawa, M. Ishida, T. Nakajima, Y. Honda, O. Kitao, H. Nakai, T. Vreven, J. A. Montgomery, Jr., J. E. Peralta, F. Ogliaro, M. Bearpark, J. J. Heyd, E. Brothers, K. N. Kudin, V. N. Staroverov, R. Kobayashi, J. Normand, K. Raghavachari, A. Rendell, J. C. Burant, S. S. Iyengar, J. Tomasi, M. Cossi, N. Rega, J. M. Millam, M. Klene, J. E. Knox, J. B. Cross, V. Bakken, C. Adamo, J. Jaramillo, R. Gomperts, R. E. Stratmann, O. Yazyev, A. J. Austin, R. Cammi, C. Pomelli, J. W. Ochterski, R. L. Martin, K. Morokuma, V. G. Zakrzewski, G. A. Voth, P. Salvador, J. J. Dannenberg, S. Dapprich, A. D. Daniels, O. Farkas, J. B. Foresman, J. V. Ortiz, J. Cioslowski, and D. J. Fox, Gaussian, Inc., Wallingford CT, 2009.
